# A conserved VPS34-PIKfyve-TRPML1-myosin II axis regulates the speed of amoeboid cell migration

**DOI:** 10.1038/s44319-026-00861-x

**Published:** 2026-07-07

**Authors:** Philippe Dehio, Céline Michard, Juan Carlos Yam-Puc, Adrià-Arnau Martí i Líndez, Anett Jandke, Gunhild Unterstab, Lucien Fabre, Loïc Sauteur, Marc Artinger, Daniel F Legler, Michael Sixt, Thorsten Schaefer, Matthias P Wymann, Klaus Okkenhaug, Thierry Soldati, Matthias Mehling, Christoph Hess

**Affiliations:** 1 Department of Biomedicine, Immunobiology, University and University Hospital of Basel, Basel, Switzerland; 2 Institute of Science and Technology Austria, Klosterneuburg, Austria; 3 Department of Biochemistry, Faculty of Science, University of Geneva, Geneva, Switzerland; 4 MRC Toxicology Unit, University of Cambridge, Cambridge, UK; 5 Department of Medicine, CITIID, University of Cambridge, Cambridge, UK; 6 Microscopy Core Facility, Department of Biomedicine, University of Basel, Basel, Switzerland; 7 Institute of Cell Biology and Immunology Thurgau, University of Konstanz, Kreuzlingen, Switzerland; 8 Theodor Kocher Institute, University of Bern, Bern, Switzerland; 9 Faculty of Biology, University of Konstanz, Konstanz, Germany; 10 Department of Biomedicine, Cancer- and Immunobiology, University of Basel, Basel, Switzerland; 11 Department of Pathology, University of Cambridge, Cambridge, UK; 12 Department of Biomedicine, Translational Neuroimmunology, University of Basel and University Hospital Basel, Basel, Switzerland

**Keywords:** Cell Adhesion, Polarity & Cytoskeleton, Immunology, Organelles

## Abstract

Amoeboid cell migration is key to efficient T cell immunity. Spatial polarization of organelles within cells, including endo-lysosomes, is a prerequisite of migration. However, how ultrastructural polarization is linked to the signaling requirements governing T cell migration remains unknown. Here we show that signaling molecules generated by endo-lysosome-localized kinases regulate velocity of amoeboid migration. Specifically, imaging of T cells identifies accumulation of endo-lysosomes decorated with the lipid kinases VPS34–PIKfyve at the uropod of polarized cells. Activity of VPS34 and PIKfyve regulates speed, but not directedness, of migrating T cells. Mechanistically, PI(3,5)P_2_ generated by the sequential action of VPS34 and PIKfyve, mediates Ca^2+^ efflux from lysosomes via the mucolipin TRP cation channel 1 (TRPML1), thus controlling activity of myosin IIA and hence the generation of propulsive force through retrograde actin flow. The VPS34–PIKfyve kinases also regulate velocity of myeloid cells, as well as of the amoeba *Dictyostelium discoideum* – establishing the axis as an evolutionarily conserved speed control system of amoeboid cell migration.

## Synopsis

The VPS34–PIKfyve pathway regulates amoeboid migration speed through endo-lysosomal Ca^2+^ release to promote myosin IIA activity. The conservation of this mechanism from *Dictyostelium discoideum* to human immune cells reveals an evolutionarily ancient role for this endo-lysosomal signaling axis in controlling cell motility.

- The VPS34–PIKfyve axis is active on endo-lysosomes at the uropod of migrating T cells.
- VPS34 and PIKfyve drive myosin IIA activation and retrograde actin flow.
- Amoeboid cell migration speed is controlled by VPS34 and PIKfyve via TRPML1.
- Regulation of amoeboid migration speed by the VPS34–PIKfyve axis is evolutionarily conserved.

## Abstract (web summary)

The VPS34–PIKfyve pathway regulates amoeboid migration speed through endo-lysosomal Ca^2+^ release to promote myosin IIA activity. The conservation of this mechanism from *Dictyostelium discoideum* to human immune cells reveals an evolutionarily ancient role for this endo-lysosomal signaling axis in controlling cell motility.

## Graphical abstract

**Figure:**
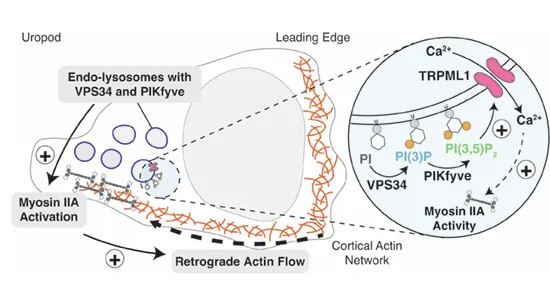

**Figure:**
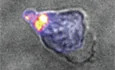

## Introduction

Cell migration is critically involved not only in immunity, but also early embryonic development, tissue homeostasis and regeneration. Across the eukaryotic domain, crawling-like, or amoeboid, movement is the most prevalent mode of cellular motility, with origins predating the emergence of metazoans (Brunet et al, 2021). Amoeboid locomotion is characterized by bleb- or pseudopod-based membrane protrusion at the leading edge and retraction of the uropod – thereby establishing the direction of motion (Tyson et al, 2014; Schick and Raz, 2022; Tsai et al, 2019; Liu et al, 2015). At the molecular level, actomyosin contractility at the uropod, and actin polymerization at the leading edge, drive a retrograde actin flow, which propels cells forward when transmitted to the extracellular space (Hons et al, 2018; Renkawitz et al, 2009; Jacobelli et al, 2004; Goor et al, 2012; Lin et al, 1996). This directed motion of F-actin is regulated via phosphorylation of the non-muscle myosin IIA regulatory light chain (RLC) (Yam et al, 2007). RLC is phosphorylated, and hence activated, by myosin light chain kinase (MLCK) in a calmodulin and cytosolic calcium (Ca^2+^) dependent process (Solanes et al, 2015; Isotani et al, 2004).

Generation of propulsive force in amoeboid migration is accompanied by, and relies on, polarization of the cellular ultrastructure. In T cells, for instance, chemokine-induced polarization and migration requires translocation of mitochondria towards the uropod to locally provide the necessary energy for myosin IIA activation (Campello et al, 2006; Morlino et al, 2014). In migrating dendritic cells (DCs), endo-lysosomes positioned at the uropod were demonstrated to support activation of myosin IIA by facilitating lysosomal Ca^2+^ release through the Ca^2+^-channel TRPML1 (Vestre et al, 2021; Bretou et al, 2017). Binding of chemokines to their respective receptors triggers engagement of Rac1/2 and RhoA GTPase activity, which is affecting cell polarity and actin polymerization (Faroudi et al, 2010; Sánchez-Madrid and Serrador, 2009). The intersection of cellular ultrastructure and related downstream signaling pathways in polarized, amoeboid migrating cells remains underexplored. Given the role of lysosomes described in migrating DCs, we hypothesized that localization of these organelles in migrating T cells serves an integrated role, physically focusing relevant signaling activity, and thus orchestrating the release of Ca^2+^ to engage myosin IIA. To test the broader relevance of key findings, non-related cell systems were explored, including the non-metazoan organism *Dictyostelium discoideum* (*D. discoideum*).

## Results

### Endo-lysosomes re-locate together with VPS34–PIKfyve to the uropod of migrating T cells

**Figure 1:**
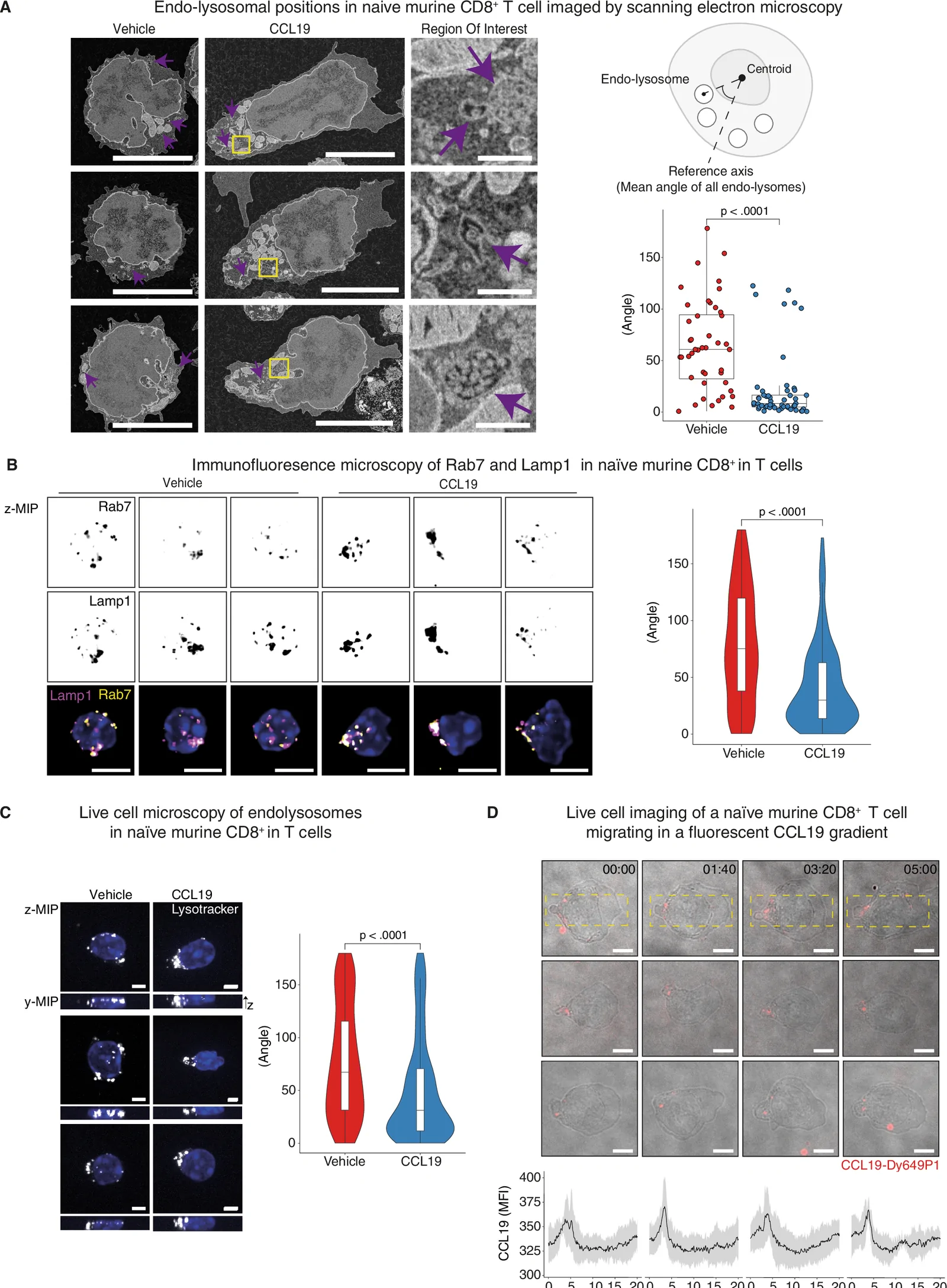
Endo-lysosomes localize to the uropod of polarized T cells. (**A**) FIB-SEM images of naive murine CD8^+^ T cells treated with vehicle control or 0.5 µg/ml CCL19 (left and middle panels; scale bar = 5 µm). Yellow boxes indicate magnified regions of interest depicted in the right panels (scale bar = 100 nm). Purple arrows mark endo-lysosomes. Top right: schematic of endo-lysosome localization analysis. Bottom right: quantification of endo-lysosomal localization (vehicle: *n* = 33; CCL19: *n* = 53; points indicate single endo-lysosomes from 7 cells). (**B**) Immunofluorescence images of endo-lysosomes in naive murine CD8^+^ T cells treated with vehicle control or 0.5 µg/ml CCL19. Left panels: representative images stained with Rab7 (top row), Lamp1 (middle row), and their overlay (bottom row; DAPI  = blue, Rab7 = purple, Lamp1 = yellow). Right panel: quantification of endo-lysosomal localization (vehicle: *n* = 582 from 18 cells; CCL19: *n* = 628 from 23 cells). Scale bar  = 5 µm. (**C**) Live-cell imaging of naive CD8^+^ T cells under agarose, stained with Hoechst and LysoTracker, and treated with vehicle control or 0.5 µg/ml CCL19. Left panels: representative merged images (Hoechst = blue, LysoTracker = white) shown as *z*- and *y* axis maximum intensity projections (MIPs). Right panel: quantification of endo-lysosomal localization (vehicle: *n* = 540 endo-lysosomes from 80 cells; CCL19: *n* = 579 from 89 cells). Scale bar = 5 µm. (**D**) Top panels: bright-field images merged with CCL19-Dy649P1 signal (red) in representative naive murine CD8^+^ T cells. Bottom panels: quantification of CCL19 signal mean fluorescence intensity (MFI; black line) and standard deviation (grey band) along the *x* axis of the dashed box in the corresponding cell images. Time stamps on the top right indicated minutes and seconds. Scale bar = 5 µm. (**B**, **C**) Representative data from ≥3 independent experiments. (**D**) Representative data from two independent experiments. Wilcoxon rank-sum test (**A**–**C**). Box plots indicate the median (central line), first and third quartiles (box bounds; 25th and 75th percentiles), and the minimum and maximum values (whiskers). Source data are available online for this figure.

**Figure EV1:**
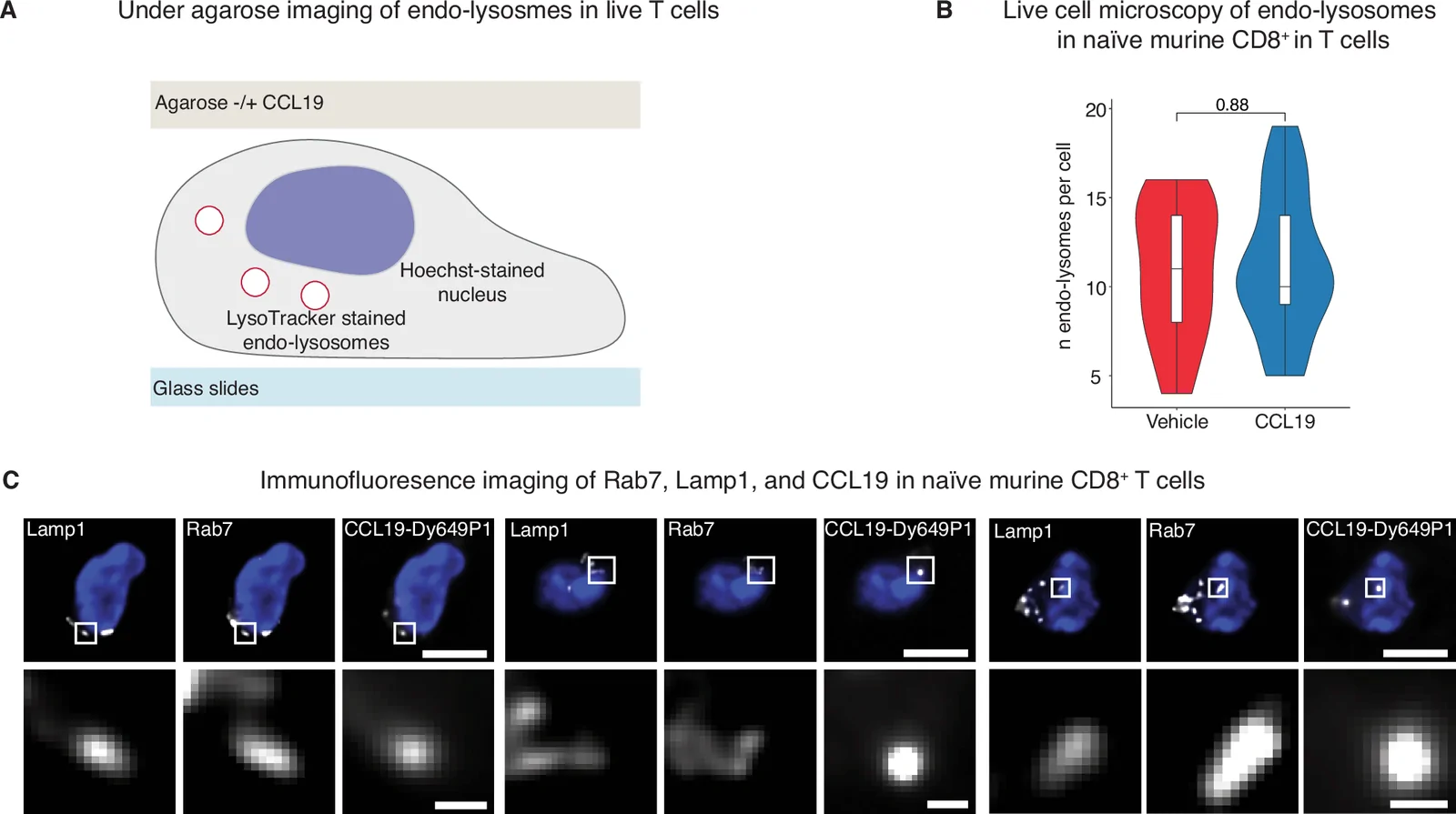
Endo-lysosomes localize to the uropod of polarized T cells. (**A**) Schematic of the under-agarose assay used to image endo-lysosomal polarization in live T cells. (**B**) Quantification of endo-lysosome numbers in naive murine CD8^+^ T cells treated with vehicle (*n* = 16) or CCL19 (*n* = 25). (**C**) Representative fluorescence images of a naive murine CD8^+^ T cell stained for Lamp1, Rab7 and exposed to CCL19-Dy649P1 (white). Top panels: merge with DAPI (blue; scale bars = 5 µm); box indicates region of interest magnified in bottom panels (scale bar = 500 nm). (**B**) Representative results of three independent experiments. Wilcoxon rank-sum test (**A**, **B**). Box plots indicate the median (central line), first and third quartiles (box bounds; 25th and 75th percentiles), and the minimum and maximum values (whiskers).

Chemokines induce polarization and migration of naive T cells, thereby offering a cellular system to study the ultrastructure of resting and migrating amoeboid cells (Garcia-Seyda et al, 2023; Ruef et al, 2023). To assess the localization of organelles among resting *vs*. chemokine-activated naive murine CD8^+^ T cells, focused ion beam scanning electron microscopy (FIB-SEM) imaging was performed. Non-stimulated T cells were roughly circular in shape, and organelles distributed around the nucleus. Activation with the chemokine CCL19 induced cells to elongate and organelles to group towards one side of the cell (= polarization), including those with morphologic characteristics of endo-lysosomes (Fig. 1A, left panels). Quantification of the angular distribution of endo-lysosomes around the centroid of the cells confirmed this observation (Fig. 1A, right panels). Immunofluorescence imaging of endo-lysosomes, using antibodies targeting Rab7 (late endosome and lysosomal marker) and Lamp1 (lysosomal marker), confirmed endo-lysosomal polarization towards one side of CCL19 activated naive murine CD8^+^ T cells (Fig. 1B). To quantify the positions of endo-lysosomes in live cells, naive murine CD8^+^ T cells stained with a lysosomal dye were confined under agarose and imaged with a spinning disk microscope (Fig. EV1A). Upon CCL19 exposure, endo-lysosomes polarized within cells, establishing that endo-lysosomes accumulated at the uropod (Fig. 1C). The total number of endo-lysosomes remained unchanged upon CCL19 exposure (Fig. EV1B). Lastly, imaging naive murine CD8^+^ T cells migrating in fluorescently tagged CCL19 (CCL19-Dy649P1) revealed focal accumulation of labelled CCL19 in intracellular vesicular structures that maintained their position throughout an observation period of up to five minutes (Fig. 1D). Immunofluorescence imaging confirmed accumulation of CCL19 in Rab7^+^ Lamp1^+^ positive regions, i.e., endo-lysosomes, in polarized naive murine CD8^+^ T cells (Fig. EV1C). In all, these imaging studies demonstrated that chemokine exposure of naive CD8^+^ T cells induced endo-lysosomal polarization to the uropod.

### T cell velocity, but not directedness, is controlled by VPS34 and PIKfyve

**Figure 2:**
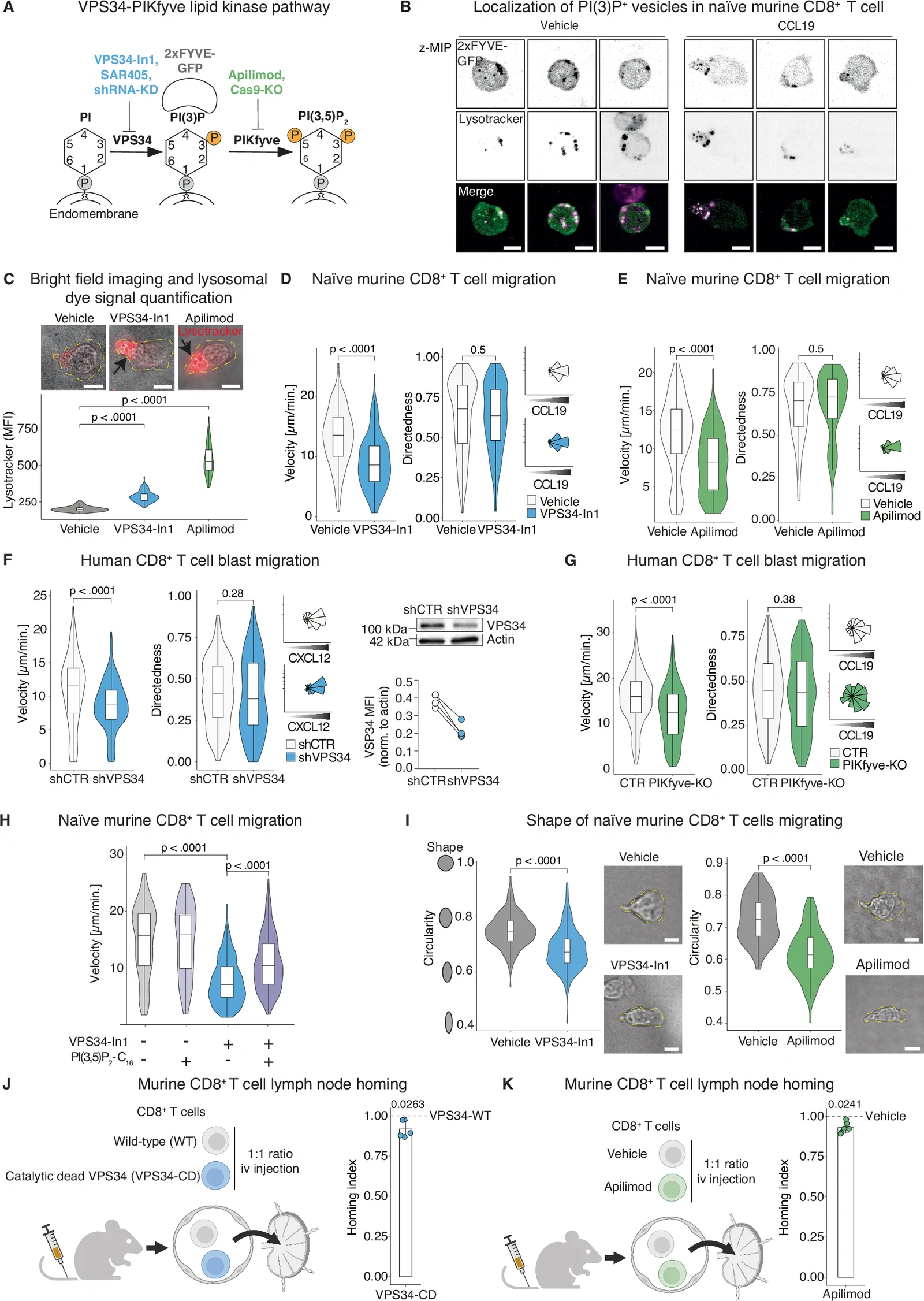
T cell velocity, but not directedness, is controlled by VPS34 and PIKfyve on endo-lysosomes. (**A**) Diagram of the VPS34–PIKfyve pathway showing inhibitors (in color), reporter (in grey), and respective targets. VPS34 and PIKfyve phosphorylate the inositol ring of phosphatidylinositol (PI) at the third and fifth positions, respectively (orange circle marked with “P”). (**B**) Representative images of naive murine CD8^+^ T cells transfected with the PI(3)P reporter 2xFYVE–eGFP (top panels), stained with LysoTracker (middle panels), and merged (2xFYVE–eGFP = green, LysoTracker = magenta*)*. Scale bar = 5 µm. (**C**) Upper panels: bright-field images overlaid with LysoTracker signal (red) of naive murine CD8^+^ T cells migrating in a CCL19 gradient, treated with vehicle control, VPS34-In1, or Apilimod. Arrows indicate large vesicles. Lower panel: quantification of lysosomal dye MFI per cell (vehicle: *n* = 60; VPS34-In1: *n* = 106; Apilimod: *n* = 73). Yellow dashed lines indicate cell outline. Scale bar = 5 µm. (**D**) Migration metrics (velocity, directedness, angle of migration) for naive murine CD8^+^ T cells treated with vehicle (velocity and angles *n* = 362, directedness *n* = 308) or VPS34-In1 (velocity and angles *n* = 193, directedness *n* = 164). (**E**) Migration metrics for naive murine CD8^+^ T cells treated with vehicle (velocity and angles *n* = 125 single cells, directedness *n* = 71) or Apilimod (velocity and angles *n* = 121, directedness *n* = 95). (**F**) Left panels: migration metrics of human CD8^+^ T cell blasts transduced with scrambled control shRNA (shCTR; velocity and angles *n* = 292, directedness *n* = 145) or VPS34-targeting shRNA (shVPS34; *n* = 255, directedness *n* = 151), placed in a CXCL12 gradient. Right panel: VPS34 knockdown efficiency as tested by Western blotting; connected dots represent samples from the same biological replicate. (**G**) Migration metrics of wild-type (velocity and angles *n* = 639, *n* = 306 for directedness) and PIKfyve-KO (velocity and angles *n* = 473, *n* = 278 for directedness) human CD8^+^ T cell blasts placed in a CCL19 gradient. (**H**) Velocity of naive murine CD8^+^ T cells in a CCL19 gradient treated with vehicle (*n* = 175), PI(3,5)P₂–C16 (*n* = 149), VPS34-In1 (*n* = 466), or PI(3,5)P₂-C16 plus VPS34-In1 (*n* = 636). (**I**) Circularity analysis and bright-field images of naive murine CD8^+^ T cells in a CCL19 gradient. Left panel: vehicle (*n* = 161) *vs*. VPS34-In1 (*n* = 157). Right panel: vehicle (*n* = 40) *vs*. Apilimod (*n* = 102). Yellow dashed lines indicate cell outline. Scale bar =  5 µm. (**J**) Schematic and results of T cell homing analysis, comparing co-transferred of wild-type *vs*. catalytically inactive VPS34 CD8^+^ T cells in a 1:1 ratio into recipient animals (*n* = 5). (**K**) Comparison of vehicle *vs*. Apilimod-treated CD8^+^ T cells in homing experiments (co-transferred cells in a 1:1 ratio into *n* = 6 recipient animals). Created with BioRender.com. (**B**–**I**) Representative data from ≥3 independent experiments. Wilcoxon rank-sum test (**C**–**G**, **I**), paired *t* test (**J**, **K**) aligned rank transform (ART) ANOVA (**F**). (**J**, **K**) Representative data from two independent experiments, Error bars = s.d. Box plots indicate the median (central line), first and third quartiles (box bounds; 25th and 75th percentiles), and the minimum and maximum values (whiskers). Source data are available online for this figure.

Endo-lysosomal membranes are signaling hubs for various pathways including the lipid-kinases VPS34 and PIKfyve, which phosphorylate phosphatidylinositol (PI) to PI(3)P and PI(3,5)P_2_, respectively (Fig. 2A) (Marat and Haucke, 2016). These kinases impact endo-lysosomal maturation, receptor recycling and Rac1 activity, features that have been associated with regulation of cell motility (Marat and Haucke, 2016; Dayam et al, 2017; Giridharan et al, 2022). This prompted us to consider their role in T cell migration. A fluorescent probe consisting of two PI(3)P-binding FYVE domains fused to an eGFP (2xFYVE–eGFP) enables subcellular localization of PI(3)P, i.e., the substrate of PIKfyve, by fluorescence imaging (Gillooly et al, 2000). 2xFYVE–eGFP accumulated on endo-lysosomal structures at the uropod of CCL19-polarized naive murine CD8^+^ T cells (Fig. 2B). Endo-lysosomal VPS34 activity thus opened the possibility for involvement of the VPS34–PIKfyve axis in regulating amoeboid cell migration. This notion was further supported by a recently published CRISPR screen that identified *PIK3C3*, the gene encoding VPS34, as a regulator of T cell migration into the central nervous system (Kendirli et al, 2023).

**Figure EV2:**
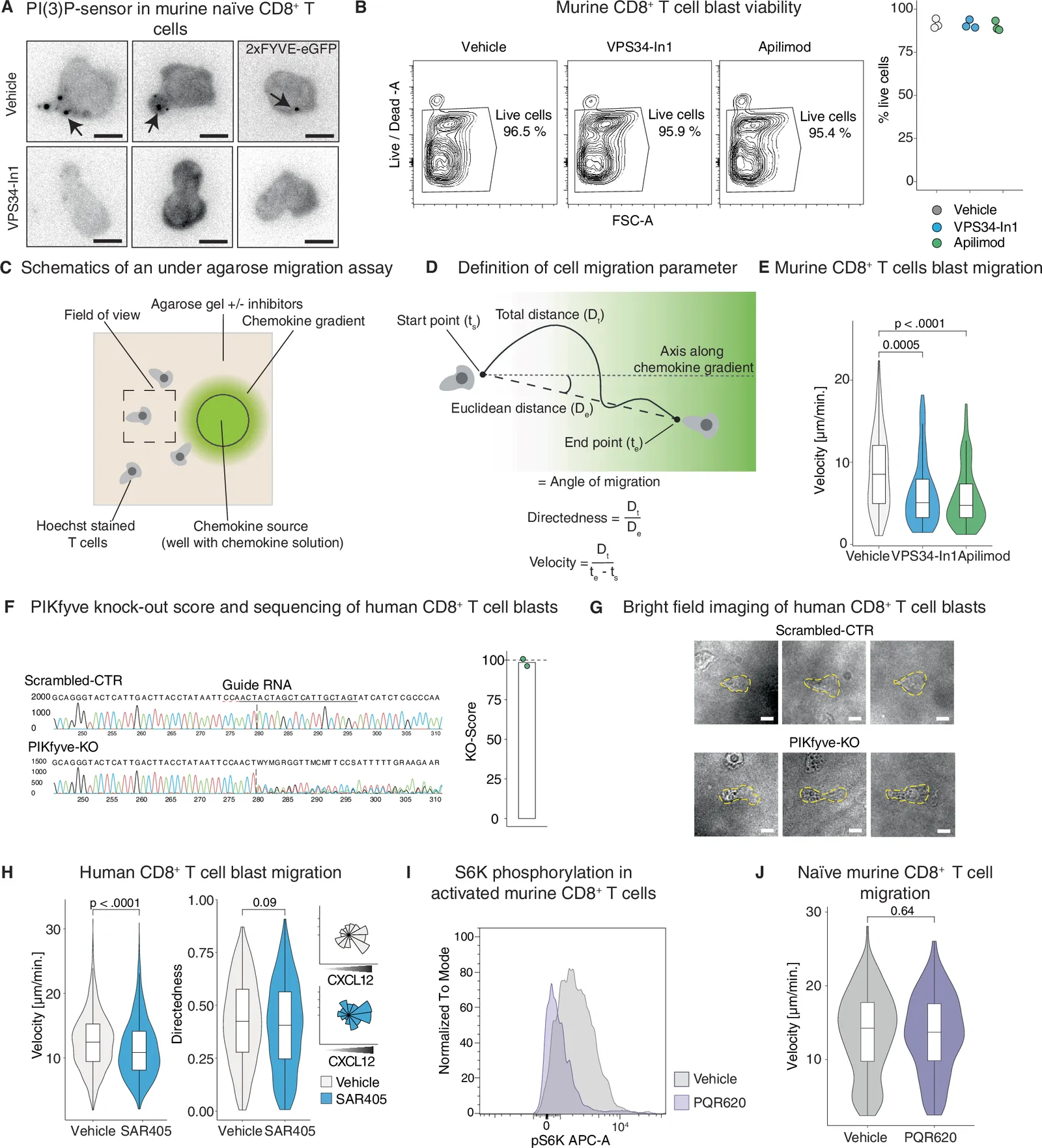
T cell velocity, but not directedness, is controlled by VPS34 and PIKfyve. (**A**) Naive murine CD8^+^ T cells transfected with the 2xFYVE–eGFP PI(3)P sensor, placed in a CCL19 gradient, and treated with vehicle or VPS34-In1. Arrows indicate PI(3)P⁺ vesicles. Scale bar = 5 µm. (**B**) Left panel: representative live/dead staining plots (flow cytometry) of murine CD8^+^ T cell blasts treated with vehicle, VPS34-In1, or Apilimod for 6 h. Right panel: percentage of live cells from three biological replicates. (**C**) Schematic (top view) of a T cell under-agarose migration assay. (**D**) Definitions of cell migration metrics used in this study. (**E**) Migration speed of murine CD8^+^ T cell blasts in a CCL19 gradient, treated with vehicle control (*n* = 66), VPS34-In1 (*n* = 81), or Apilimod (*n* = 79). (**F**) Left: representative bulk Sanger sequencing of control *vs*. PIKfyve-KO human CD8^+^ T cell blasts of the region flanking the first guide RNA (gRNA). The gRNA sequence is underlined, the cutting side indicated by a dashed vertical line. Right: knockout score (*n* = 2). (**G**) Bright-field images of control and PIKfyve-KO human CD8^+^ T cells placed in a CCL19 gradient. Yellow dashed lines indicate cell outline. Scale bar = 10 µm. (**H**) Velocity and directedness of human CD8^+^ T cell blasts migrating in a CXCL12 gradient, treated with the VPS34 inhibitor SAR405 (velocity and angles: *n* = 1838, directedness: *n* = 694) or vehicle control (velocity and angles: *n* = 1400, directedness: *n* = 594). (**I**) Representative histogram (flow cytometry) of pS6K expression among murine CD8^+^ T cells activated for 24 h with CD3 and CD28 targeting antibodies and treated with the mTOR inhibitor PQR620 (purple) *vs*. vehicle control (grey). (**J**) Velocity of naive murine CD8^+^ T cells in a CCL19 gradient, following treatment with vehicle (*n* = 272) or the mTOR inhibitor PQR602 (*n* = 232). (**A**, **B**, **G**–**J**) Representative of ≥3 independent experiments; (**I**) representative of two independent experiments. Wilcoxon rank-sum test (**E**, **H**, **J**). Box plots indicate the median (central line), first and third quartiles (box bounds; 25th and 75th percentiles), and the minimum and maximum values (whiskers).

Using inhibitors of VPS34 and PIKfyve, we first explored how activity of these kinases related to accumulation of endo-lysosomes in polarized T cells. Inhibition of VPS34 in our system, using the potent and selective inhibitor VPS34-In1, was ascertained by imaging studies (Fig. EV2A) (Bago et al, 2014). Apilimod is a highly selective PIKfyve inhibitor in clinical development (Cai et al, 2013; Gayle et al, 2017). Importantly, both inhibitors do not affect other lipid kinases, such as class 1 PI3K (e.g., PI3Kγ), which have been implicated in T cell migration (Nombela-Arrieta et al, 2007). At 2 µM, i.e., the concentration used throughout, neither VPS34-In1 nor Apilimod affected the viability of murine CD8^+^ T cell blasts after 6 h of treatment (Fig. EV2B). With these tools in hand, we blocked VPS34 or PIKfyve in CCL19 stimulated naive murine CD8^+^ T cells and examined cells using brightfield imaging. Inhibiting either kinase resulted in the appearance of large vesicles at the uropod of polarized cells (Fig. 2C, upper panel). The fluorescent signal from a lysosomal dye was also increased in VPS34-In1 as well as Apilimod-treated naive murine CD8^+^ T cells, indicative of an increase in overall endo-lysosomal volume (Fig. 2C, lower panel). These findings align with the known role of the VPS34–PIKfyve system in endo-lysosomal homeostasis (Marat and Haucke, 2016).

We then went on and tested how inhibiting VPS34 or PIKfyve impacted T cell motility, using an under-agarose migration assay (Fig. EV2C). Inhibition of VPS34 and PIKfyve both reduced the migration velocity of naive murine CD8^+^ T cells, while not altering directedness and angle of migration towards the source of chemokine (Figs. 2D,E and EV2D). VPS34-In1 and Apilimod also reduced the speed of murine CD8^+^ T cell blasts in a CCL19 gradient (Fig. EV2E). shRNA-mediated silencing of VPS34 in human CD8^+^ T cell blasts likewise decreased their speed, while preserving directedness and angles of migration (Fig. 2F). A CRISPR-Cas9 knock-out of PIKfyve in human CD8^+^ T cells (Fig. EV2F) phenocopied inhibition of PIKfyve, reducing migration speed and inducing aberrant vesiculation at the uropod (Figs. 2G and EV2G). Finally, the structurally non-related, selective VPS34 inhibitor SAR405 also phenocopied migration deficits induced by inhibition of VPS34 and PIKfyve inhibition in human CD8^+^ T cell blasts migrating in a CXCL12 gradient (Fig. EV2H) (Ronan et al, 2014). To test whether the product of the VPS34–PIKfyve axis, i.e., PI(3,5)P_2_, was underpinning the observed phenotype, cell culture medium was supplemented with dipalmitoyl-PI(3,5)P_2_. Evidencing the importance of PI(3,5)P_2_, reduced migration velocity imposed by inhibition of VPS34 in naive murine CD8^+^ T cells was partially rescued by addition of this synthetic PI(3,5)P_2_ (Fig. 2H).

VPS34 activity has previously been reported to be controlled by the mechanistic target of rapamycin (mTOR), specifically in the context of cell starvation (Munson et al, 2015; Yuan et al, 2013). mTOR, in conjunction with the Ragulator–Rag complex, integrates environmental signals (e.g., nutrients and cytokine signaling) on lysosomes (Betz and Hall, 2013). mTOR has been suggested to be activated by chemokine signaling, and regulate T cell migration (Haas et al, 2015; Munk et al, 2011; Murooka et al, 2008). We thus reasoned that mTOR signaling might regulate VPS34 and PIKfyve activity on endo-lysosomes at the uropod, and in turn regulate T cell migration. Using the selective mTOR1/2 inhibitor PQR620, we blocked mTOR activity in murine naive CD8^+^ T cells migrating in a CCL19 gradient (Rageot et al, 2018). The mTOR blocking activity of PQR620 was confirmed by assessing phosphorylation of the mTOR target S6K by flow cytometry in activated CD8^+^ T cells (Fig. EV2I). Inhibiting mTOR in these short-term exposure experiments did not affect velocity of naive murine CD8^+^ T cells in (Fig. EV2J), possibly due to low mTOR activity in naive T cells (Haas et al, 2015; Munk et al, 2011; Murooka et al, 2008; Chi, 2012).

**Figure EV3:**
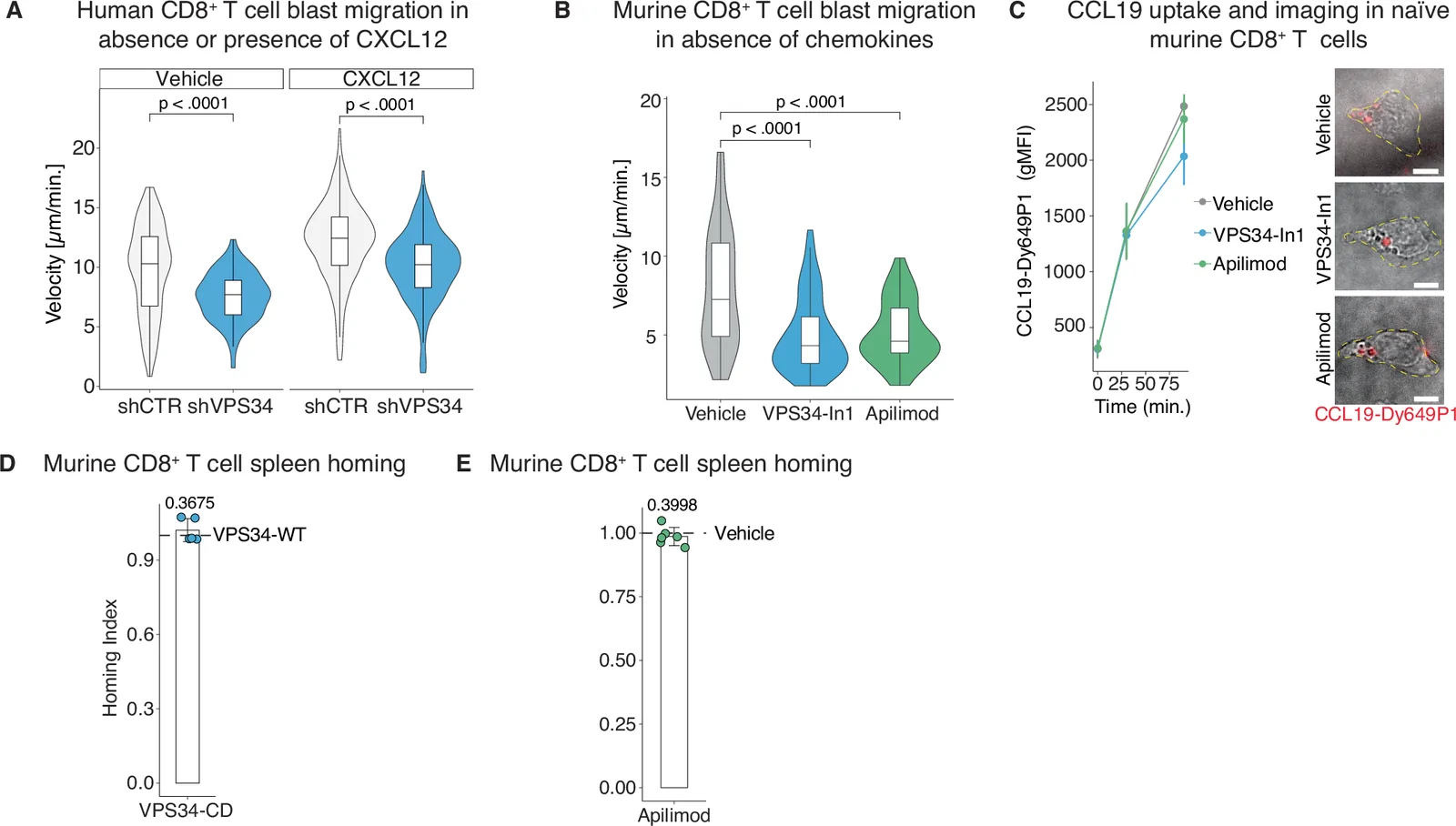
Chemokine signaling is dispensable for VPS34–PIKfyve-mediated regulation of T cell migration speed. (**A**) Migration speed in the absence or presence of a CXCL12 gradient, of human CD8^+^ T cell blasts expressing scrambled control shRNA (shCTR; vehicle: *n* = 196; CXCL12: *n* = 326) or VPS34 knockdown (shVPS34; vehicle: *n* = 62; CXCL12: *n* = 210). (**B**) Migration speed of murine CD8^+^ T cell blasts treated with vehicle (*n* = 90), VPS34-In1 (*n* = 76), or Apilimod (*n* = 46) in the absence of chemokines. (**C**) Left panel: Time course analysis of CCL19-Dy649P1 uptake in naive murine CD8^+^ T cells (flow cytometry, geometric mean intensity (gMFI) and standard deviation of three biological replicates). Right panel: Representative bright field images merged with CCL19-Dy649P1 signal (red) of naive murine CD8^+^ T cells migrating in a fluorescent CCL19 gradient, treated with vehicle control, VPS34-In1, or Apilimod. Yellow dashed lines indicate cell outline. Scale bar = 5 µm. (**D**) Spleen homing index (normalized to each internal control) of murine CD8^+^ T cells per recipient mice (*n* = 5) expressing the wild-type or catalytic dead (CD) VPS34, and (**E**) spleen homing index (normalized to each internal control, *n* = 6 recipient mice) of vehicle or Apilimod-treated cells. (**A**–**C**) Representative of three experiments; (**D**, **E**) representative of two experiments. Wilcoxon rank-sum test (**A**, **B**) and paired *t* test (**D**, **E**). (**D**, **E**) Error bars = s.d. Box plots indicate the median (central line), first and third quartiles (box bounds; 25th and 75th percentiles), and the minimum and maximum values (whiskers).

T cell blasts polarize and migrate in absence of chemokines and thus allow exploring whether chemokine signalling is required for the here observed VPS34–PIKfyve dependent migration phenotype (Galeano Niño et al, 2020). First, we compared migration of human CD8^+^ T cell blasts with a VPS34 knock-down *vs*. their respective control, in absence or presence of the chemokine CXCL12. VPS34 knock-down reduced cell migration speed irrespective of CXCL12 exposure, while chemokine exposure increased overall migration speed (Fig. EV3A). Likewise, migration speed of murine CD8^+^ T cell blasts in absence of chemokines was also lowered by inhibition of VPS34 or PIKfyve (Fig. EV3B). To test whether uptake and vesicular accumulation of chemokines at the uropod was affected by inhibition of VPS34 or PIKfyve, naive murine CD8^+^ T cells were activated using fluorescent CCL19 in presence or absence of VPS34-In1 or Apilimod, respectively. Neither inhibition with VPS34-In1 nor Apilimod affected uptake of CCL19, as assessed by (i) flow cytometry after 30 or 90 min (Fig. EV3C, left panel), and (ii) accumulation of fluorescence in vesicular structures at the uropod (Fig. EV3C, right panel). This suggested that neither uptake of chemokine nor cell polarization were affected by inhibition of the VPS34–PIKfyve axis. Together, these data indicate that chemokine signalling is dispensable for regulating T cell speed by the VPS34–PIKfyve axis.

Rapidly migrating T cells tend to be elongated and less circular (Hons et al, 2018). Notably, inhibition of VPS34 and PIKfyve, thus slowing their migration, also decreased the average circularity of naive murine CD8^+^ T cells migrating in a CCL19 gradient (Fig. 2I). This phenotypic constellation (decreased migration speed among less circular cells) was compatible with insufficient myosin IIA activity in VPS34—PIKfyve inhibited cells—a notion further explored below (Morin et al, 2008; Smith et al, 2003; Tsai et al, 2019). To test whether migration of CD8^+^ T cells was impacted by VPS34 and PIKfyve also in vivo, adoptive transfer experiments were performed. Specifically, murine CD8^+^ T cells expressing either wild-type or a catalytic inactive form of VPS34 were injected in a 1:1 ratio into the tail vein of recipient mice, and their homing into lymph nodes (inguinal, axillary, cervical) was quantified (Fig. 2J, left panel) (Courreges et al, 2024). In these experiments, the number of wild-type CD8^+^ T cells migrating into lymph nodes was modestly but consistently higher than that of cells expressing catalytic inactive VPS34 (Fig. 2J, right panel). Likewise, pre-treatment of murine CD8^+^ T cells with Apilimod reduced their lymph node homing relative to vehicle-treated control cells (Fig. 2K). Frequencies of transferred CD8^+^ T cells in the spleen were similar to input, irrespective of VPS34 and PIKfyve sufficiency *vs*. insufficiency (Fig. EV3D,E)—aligning with passive accumulation in the red pulp and marginal zone compartment (Tadayon et al, 2019; Chauveau et al, 2020).

Together, these experiments identified lipid kinase activity along the PI→PI(3)P→PI(3,5)P_2_ pathway, governed by VPS34 and PIKfyve at the uropod of T cells, to determine endo-lysosomal volume and velocity of migrating CD8^+^ T cells, while not affecting chemotactic directedness.

### VPS34 and PIKfyve regulate retrograde actin flow and myosin IIA activation

**Figure 3:**
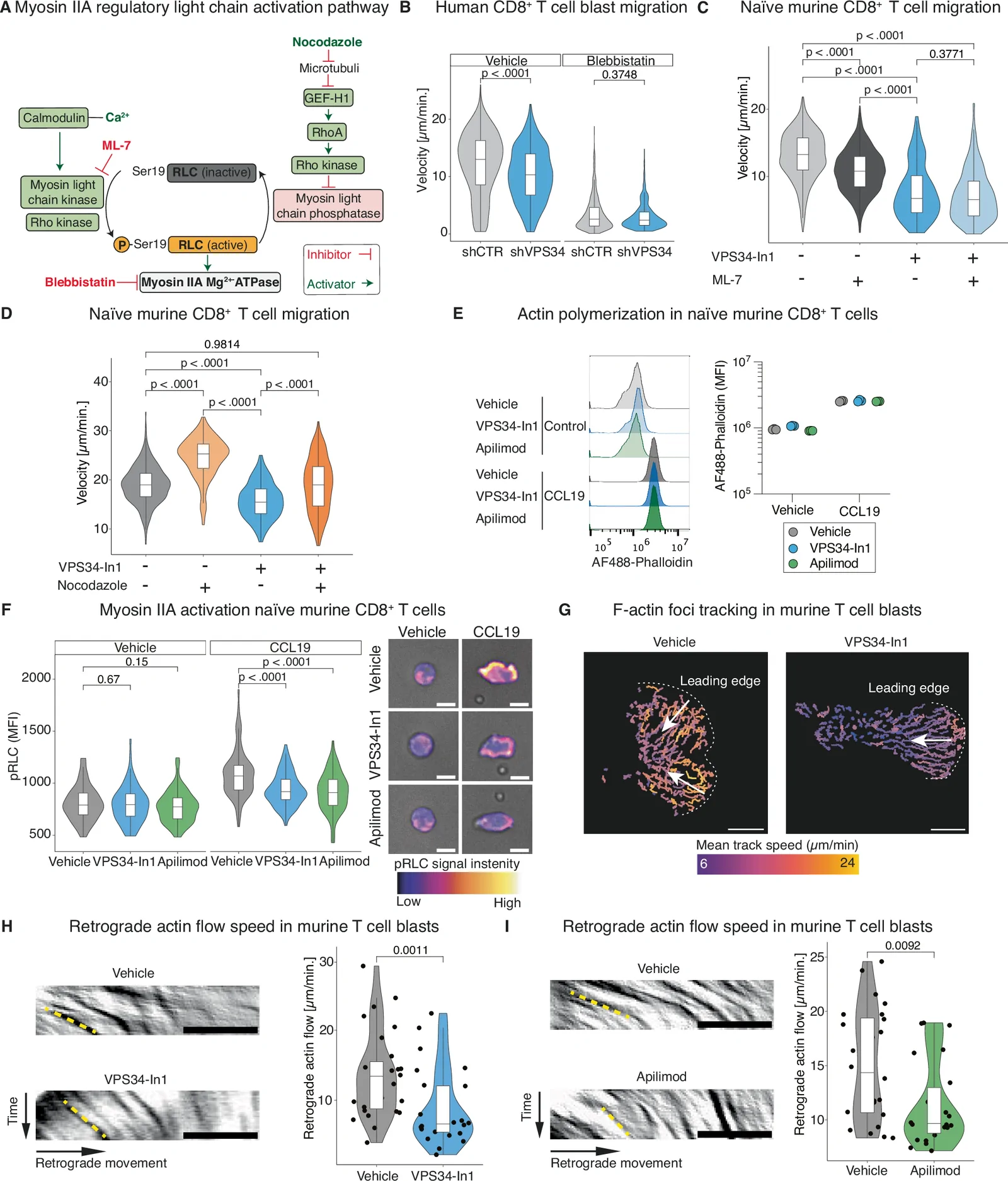
VPS34 and PIKfyve regulate retrograde actin flow and myosin IIA activation. (**A**) Schematic of the myosin IIA regulatory light chain activation pathway. Inhibitors or inhibitory interactions are shown in red, activators or activating interactions in green. (**B**) Velocity of human CD8^+^ T cell blasts transduced with control shRNA (*n* = 1037 for vehicle control; *n* = 759 for Blebbistatin) or VPS34-shRNA (*n* = 534 for vehicle control; *n* = 594 for Blebbistatin), placed in a CXCL12 gradient in presence *vs*. absence of Blebbistatin. (**C**) Velocity of naive murine CD8^+^ T cells in a CCL19 gradient treated with vehicle control (*n* = 815), ML-7 (*n* = 1019), VPS34-In1 (*n* = 199), and ML-7 & VPS34-In1 (*n* = 238). (**D**) Velocity of naive murine CD8^+^ T cells in a CCL19 gradient treated with vehicle control (*n* = 439), Nocodazole (*n* = 185), VPS34-In1 (*n* = 243), and Nocodazole & VPS34-In1 (*n* = 336). (**E**) Left panel: Representative flow cytometry histograms of F-actin staining (AF488-Phalloidin) in naive murine CD8^+^ T cells assessed one minute after treatment with vehicle control or CCL19. Right panel: Phalloidin mean fluorescence intensity (MFI, flow cytometry) of three technical replicates. (**F**) Left panel: Per cell MFI signal (fluorescence microscopy) among naive murine CD8^+^ T cells stained for myosin IIA RLC phosphorylated at serine 19 (pRLC) and treated with vehicle control (no CCL19: *n* = 81; CCL19: *n* = 75), VPS34-In1 (no CCL19: *n* = 79; CCL19: *n* = 88), and Apilimod (no CCL19: *n* = 139; CCL19: *n* = 64). Right panels: Representative bright-field images of cells overlaid with pseudo-colored pRLC signal. Scale bar = 5 µm. (**G**) Representative tracks of F-actin foci derived from actin-reporter T cell blasts with or without VPS34-In1, pseudo-colored according to speed. Dashed lines denote the leading edge; arrows indicate the direction of actin flow. Scale bar = 5 µm. (**H**) Left panel: Representative kymograph of F-actin reporter signal along the cell’s axis over a 30 sec period. The yellow dashed line indicates an exemplary actin trace used for speed analysis. Scale bar =  5 µm. Right panel: Kymograph-based speed analysis of retrograde actin flow in actin-reporter T cell blasts treated with vehicle control (*n* = 27) or VPS34-In1 (*n* = 24). (**I**) Left panel: Representative kymograph of F-actin reporter signal along the cell’s axis over a 30 s period. The yellow dashed line indicates an exemplary actin trace used for speed analysis. Right panel: Kymograph-based speed analysis of retrograde actin flow in actin-reporter T cell blasts treated with vehicle control (*n* = 25) or Apilimod (*n* = 26). Scale bar = 5 µm. (**B**–**I**) Represent results from ≥3 independent experiments. Wilcoxon rank-sum test (**F**, **H**, **I**), ART ANOVA (**B**–**D**). Box plots indicate the median (central line), first and third quartiles (box bounds; 25th and 75th percentiles), and the minimum and maximum values (whiskers). Source data are available online for this figure.

**Figure EV4:**
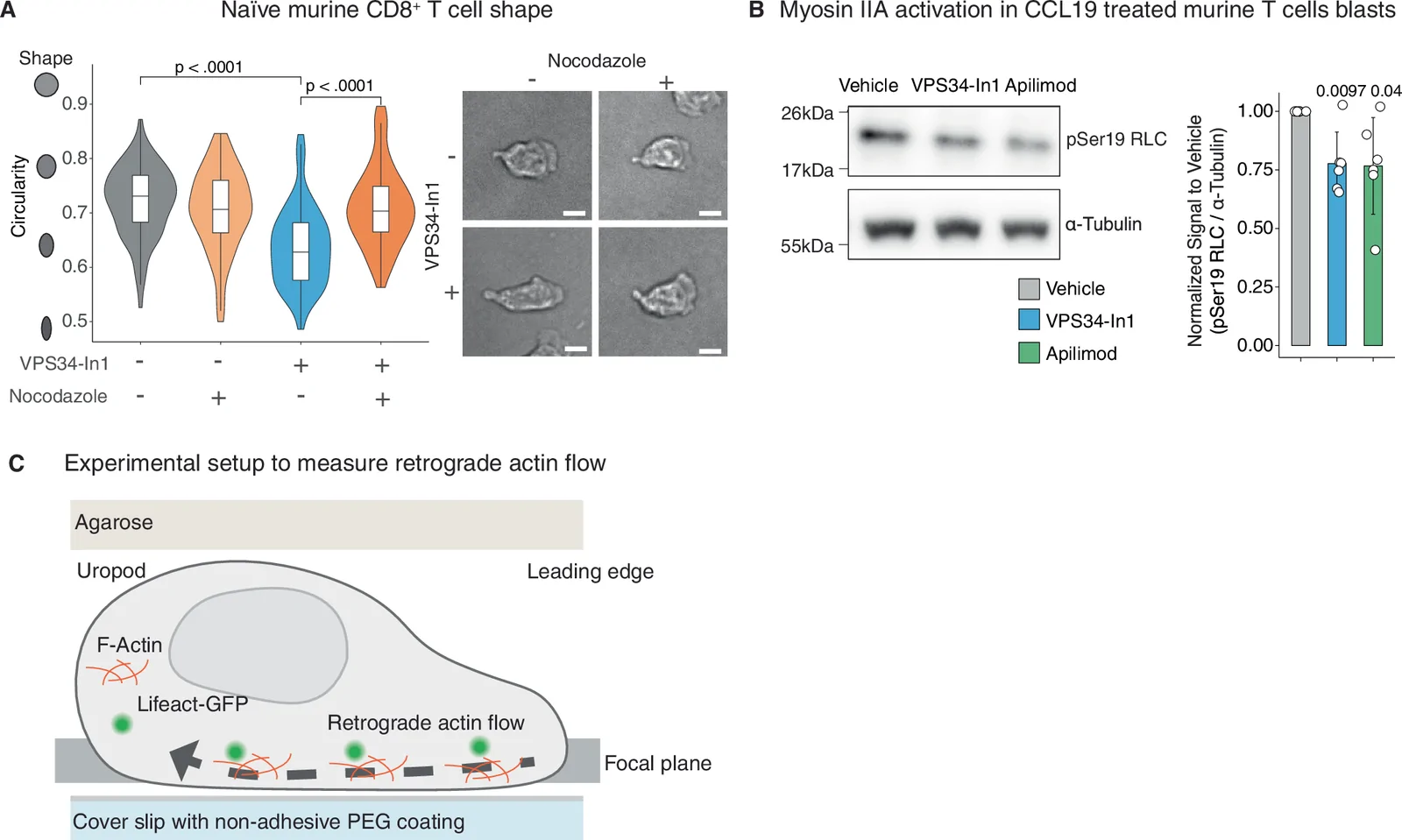
VPS34 and PIKfyve regulate retrograde actin flow and myosin IIA activation. (**A**) Left panel: Circularity of naive CD8^+^ T cells placed in a CCL19 gradient treated with vehicle control (*n* = 81), Nocodazole (*n* = 70), VPS34-In1 (*n* = 85), and VPS34-In1 plus Nocodazole (*n* = 56). Right panels: representative bright field images. Scale bar: 5 µm. (**B**) Left panels: representative Western blots probing for RLC Ser19 phosphorylation and α-tubulin (loading control) in T cell blasts treated with CCL19 and vehicle, VPS34-In1, or Apilimod. Right panel: quantification of six biological replicates, normalized to α-tubulin and vehicle. (**C**) Experimental setup for assessing retrograde actin flow in murine T cell blasts expressing the Lifeact-GFP reporter (to visualize F-actin dynamics). (**A**) Displays representative data of three independent experiments. ART ANOVA for (**A**). Paired *t* test for (**B**). (**B**) Error bars = s.d. Box plots indicate the median (central line), first and third quartiles (box bounds; 25th and 75th percentiles), and the minimum and maximum values (whiskers).

The flow of actin—i.e., the propulsion force underlying amoeboid cell migration—relies on synchronized actin polymerization at the leading edge, and myosin IIA-induced F-actin traction at the uropod (Hons et al, 2018; Renkawitz et al, 2009; Goor et al, 2012; Lin et al, 1996). The importance of myosin IIA activity in governing cell migration was specifically verified for T cells by using two distinct approaches. First, direct (Blebbistatin), as well as indirect (ML-7), inhibition of myosin IIA activity reduced migration speed of human CD8^+^ T cell blasts as well as human naive CD8^+^ T cells, irrespective of upstream VPS34 activity (Fig. 3A–C). Second, Nocodazole, which increases myosin IIA activity, increased velocity of migration both in presence and absence of VPS34 blockade, and also restored decreased circularity induced by inhibition of VPS34 to baseline levels (Figs. 3A,D and  EV4A) (Chang et al, 2008; Kolodney and Elson, 1995; Liu et al, 1998; Kopf et al, 2020; Hui and Upadhyaya, 2017). Given these unambiguous data, we hypothesized that the VPS34–PIKfyve system impacted migration via regulating the activity of myosin IIA—an alternative possibility being an impact of the kinase system on actin polymerization. The latter idea was refuted by the finding that actin polymerization in CCL19-treated naive murine CD8^+^ T cells was unaffected upon inhibition of either VPS34 or PIKfyve (Fig. 3E). By contrast, in this same system, myosin IIA activity—read out by the phosphorylation status of RLC—was diminished upon blocking of either kinase (Figs. 3F and EV4B), also aligning with the elongation observed among T cells migrating in presence of VPS34 or PIKfyve inhibitors (Fig. 2I).

To dynamically visualize dependency of actin flow on the VPS34–PIKfyve system, T cell blasts from Lifeact-GFP expressing mice were confined by serum-free agarose on a non-adhesive coating and activated by CCL19 contained in the polymer (Fig. EV4C) (Riedl et al, 2008). Indeed, inhibition of either VPS34 or PIKfyve reduced the speed of cortical F-actin foci moving within cells (Fig. 3G-I).

Collectively, these findings established that VPS34 and PIKfyve regulated retrograde actin flow—the main propulsion mechanism for T cell migration—via tuning activity of myosin IIA.

### VPS34 and PIKfyve regulate migration velocity of T cells via lysosomal Ca^2+^

**Figure 4:**
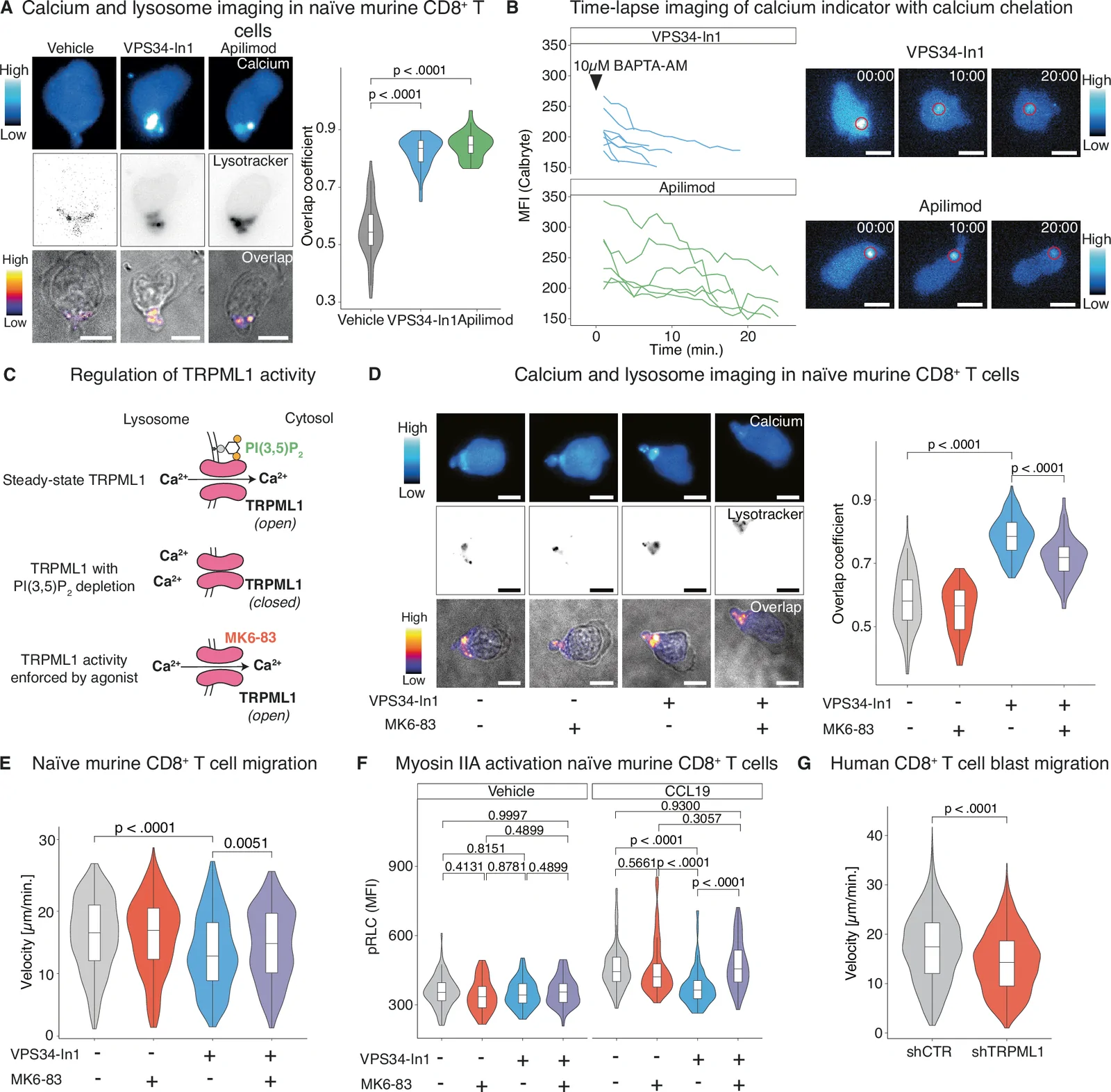
VPS34 and PIKfyve regulate migration velocity of T cells via lysosomal Ca^2+^. (**A**) Left panels: Fluorescence images of naive murine CD8^+^ T cells stained with a calcium indicator dye (Calbryte) and a lysosomal dye (LysoTracker). Bright field images were merged with a pseudo color map indicating the overlap of calcium and lysosomal signals (Pearson correlation coefficient). Right panel: Overlap coefficient ( = colocalization) of calcium and lysosomes in cells treated with vehicle control (*n* = 45), VPS34-In1 (*n* = 44), or Apilimod (*n* = 37). Scale bar = 5 µm. (**B**) Left panel: Time-course analysis of calcium indicator signal (MFI) in large vesicles induced by VPS34-In1 (*n* = 8) or Apilimod (*n* = 6) after BAPTA-AM addition in naive murine CD8^+^ T cells. Right panels: Representative calcium indicator images with tracked vesicle*s*, marked by a red circle. Scale bar = 5 µm. (**C**) Schematic of TRPML1 activation by the TRPML1 agonist PI(3,5)P_2_ and MK6-83. (**D**) Left panels: Fluorescence images of naive murine CD8^+^ T cells stained with a calcium indicator dye (Calbryte) and a lysosomal dye (LysoTracker). Bright field images were merged with a pseudo color map indicating the overlap of calcium and lysosomal signals (Pearson correlation coefficient). Scale bar = 5 µm. Right panel: Overlap coefficient ( = colocalization) of calcium and lysosomes in cells treated with vehicle (*n* = 94), VPS34-In1 (*n* = 120), MK6-83 (*n* = 65) or VPS34-In1 & MK6-83 (*n* = 122). (**E**) Velocity of naive murine CD8^+^ T cells migrating in a CCL19 gradient, treated with vehicle control (*n* = 560), VPS34-In1 (*n* = 408), MK6-83 (*n* = 418), and a combination of VPS34-In1 plus MK6-83 (*n* = 580). (**F**) Per cell MFI signal (fluorescence microscopy) among naive murine CD8^+^ T cells stained for pRLC and treated with vehicle control (no CCL19: *n* = 83; CCL19: *n* = 110), VPS34-In1 (no CCL19: *n* = 72; CCL19: *n* = 113), MK6-83 (no CCL19: *n* = 47; CCL19: *n* = 72), and VPS34-In1 plus MK6-83 (no CCL19: *n* = 71; CCL19: *n* = 74). (**G**) Migration velocities of human CD8^+^ T cell blasts treated with scrambled control shRNA (shCTR; *n* = 1972) or TRPML1 knockdown (shTRPML1; *n* = 1550) in a CCL19 gradient. (**A**, **B**, **D**–**G**) Representative results of ≥3 independent experiments. Wilcoxon rank-sum test (**A**, **G**); ART ANOVA (**D**–**F**). Box plots indicate the median (central line), first and third quartiles (box bounds; 25th and 75th percentiles), and the minimum and maximum values (whiskers). Source data are available online for this figure.

**Figure EV5:**
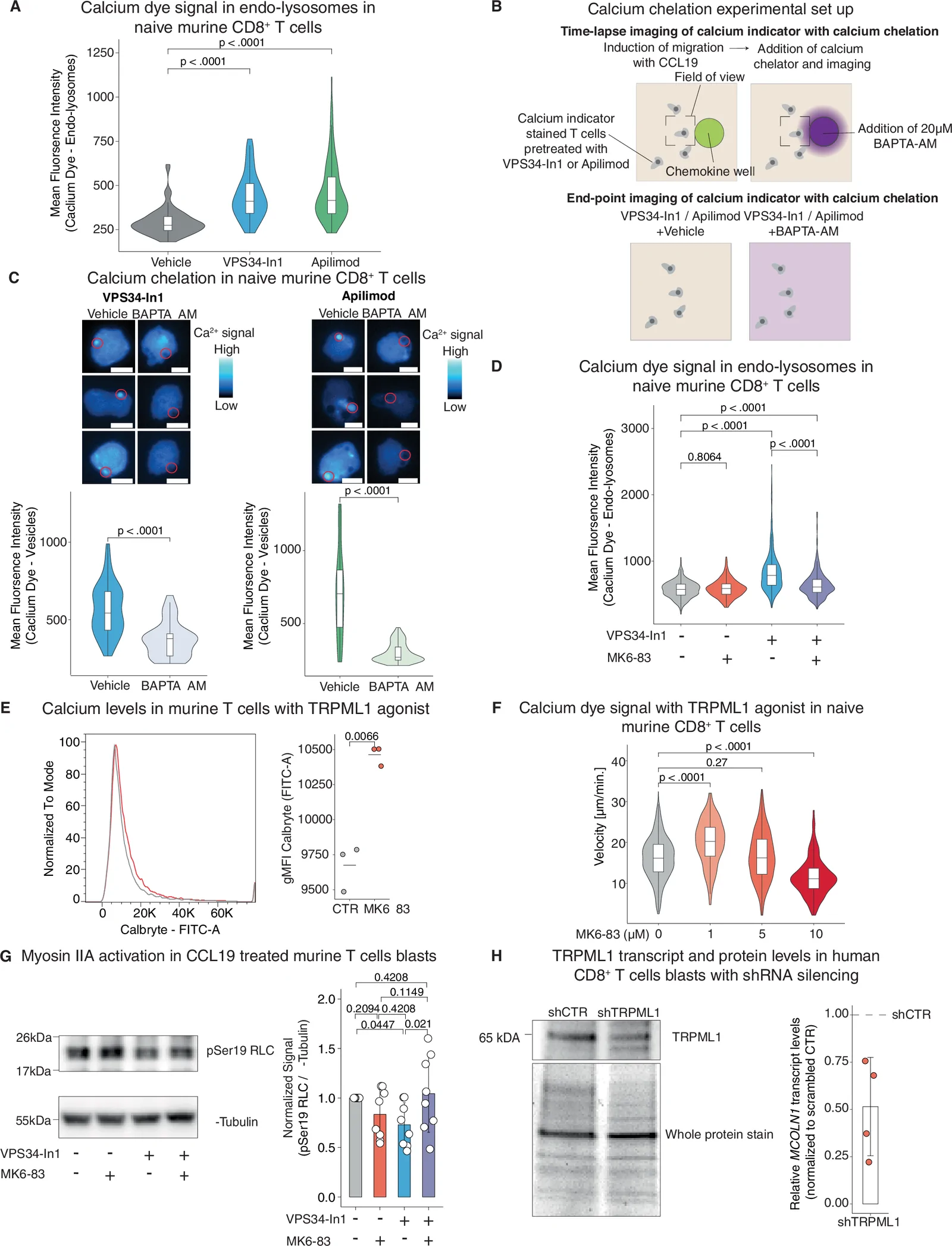
VPS34 and PIKfyve regulate migration velocity of T cells via lysosomal Ca^2+^. (**A**) Quantification of calcium dye MFI per endo-lysosome in naive murine CD8^+^ T cells migrating in a CCL19 gradient, treated with vehicle (*n* = 82 vesicles in 28 cells), VPS34-In1 (*n* = 75 vesicles in 19 cells), or Apilimod (*n* = 152 vesicles in 39 cells). (**B**) Experimental setup for calcium chelation: naive murine CD8^+^ T cells stained with Calbryte were treated with VPS34-In1 or Apilimod and placed under agarose. For time-lapse imaging, cells migrated in a CCL19 gradient, then medium was replaced with BAPTA-AM-containing medium. For endpoint imaging, cells were incubated for 30 min at 37 °C with or without VPS34-In1, Apilimod, and/or BAPTA-AM. (**C**) Upper panels: representative images of Calbryte-stained naive murine CD8^+^ T cells with or without VPS34-In1, Apilimod, and/or BAPTA-AM. Lower panels: quantification of calcium dye MFI per endo-lysosome for VPS34-In1 (*n* = 72 vesicles in 32 cells), VPS34-In1 + BAPTA-AM (*n* = 41 vesicles in 18 cells), Apilimod (*n* = 25 vesicles in 10 cells), or Apilimod plus BAPTA-AM (*n* = 64 vesicles in 23 cells). Representative quantified vesicles are marked by a red circle. Scale bar = 5 µm. (**D**) Quantification of calcium dye MFI per endo-lysosome in naive murine CD8^+^ T cells migrating in a CCL19 gradient, treated with vehicle (*n* = 335 vesicles in 93 cells), MK6-83 (*n* = 194 vesicles in 65 cells), VPS34-In1 (*n* = 518 vesicles in 108 cells), or VPS34-In1 plus MK6-83 (*n* = 450 vesicles in 121 cells). (**E**) Left panel: Representative histogram of cytosolic calcium levels as captured by flow cytometry in murine T cells stained with Ca²⁺ dye and treated with MK6-83 (5 µM) or vehicle. Right panel: Summary of *n* = 3 technical replicates. (**F**) Migration velocity of naive murine CD8^+^ T cells placed in a CCL19 gradient, treated with vehicle (*n* = 646), or increasing concentrations of MK6-83 (1 µM: *n* = 173; 5 µM: *n* = 487; 10 µM: *n* = 262). (**G**) Left panel: representative Western blots probing for RLC Ser19 phosphorylation (top) and α-tubulin (bottom; loading control) in T cell blasts treated with CCL19 and vehicle, VPS34-In1, and/or MK6-83. Right panel: quantification of eight biological replicates normalized to α-tubulin and vehicle. (**H**) Left panel: representative Western blots probing for TRPML1 (*top*) and whole protein stain (bottom) in human CD8^+^ T cells transduced with scrambled control shRNA (shCTR) or TRPML1-targeting shRNA (shTRPML1). Right panel: relative *MCOLN1* transcript levels from four biological replicates, normalized to SDHA and scrambled control. (**A**, **C**, **D**, **F**) Representative of ≥3 independent experiments. Two-way ANOVA with Fisher’s least-squares test (**G**), *t* test (**E**), Wilcoxon rank-sum test (**A**, **C**), ART ANOVA (**D**). (**G**, **H**) Error bars = s.d. Box plots indicate the median (central line), first and third quartiles (box bounds; 25th and 75th percentiles), and the minimum and maximum values (whiskers).

We next aimed to explore how activity of the VPS34–PIKfyve axis, myosin IIA activity, and T cell migration were mechanistically interlinked. Endo-lysosomes function as Ca^2+^ stores, and cytosolic Ca^2+^ promotes activation of myosin IIA (Fig. 3A) (Vestre et al, 2021; Bretou et al, 2017). We therefore imaged intracellular Ca^2+^ in naive murine CD8^+^ T cells migrating in a CCL19 gradient in presence or absence of inhibitors for VPS34 and PIKfyve, respectively. Inhibition of either kinase resulted in a notable increase in lysosomal Ca^2+^ (Figs. 4A and EV5A). Substantiating specificity of this signal, chelating intracellular Ca^2+^ with BAPTA-AM caused progressive fading of the calcium indicator in lysosomes and reduced signal in end-point measurements (Figs. 4B and EV5B,C).

The flux of Ca^2+^ from lysosomes to the cytoplasm is gated by TRPML1, a process regulated by PI(3,5)P_2_ (Fig. 4C) (Gan et al, 2022; Dong et al, 2010; Feng et al, 2014). We thus hypothesized that inhibition of VPS34 or PIKfyve, and thus depletion of PI(3,5)P_2_, hinders lysosomal Ca^2+^ efflux – leading to accumulation of lysosomal Ca^2+^ in migrating naive murine CD8^+^ T cells. Functional engagement of TRPML1 should thus restore lysosomal Ca^2+^ efflux and reverse VPS34-dependent migration deficits in T cells. To test this idea, we monitored migration of naive murine CD8^+^ T cells in presence or absence of VPS34-In1 and/or the specific and potent TRPML1 agonist MK6-83 (Fig. 4C) (Chen et al, 2014). Indeed, 5 µM MK6-83 reduced lysosomal Ca^2+^ sequestration and partially rescued the speed deficit imposed by inhibition of VPS34 (Figs. 4D,E and EV5D). The activity of 5 µM MK6-83 was confirmed by flow cytometry in murine T cells stained with a Ca^2+^ dye (Fig. EV5E). MK6-83 titration experiments revealed a dose dependent regulation of T cell speed, with low levels of TRPML1 activation increasing and high doses dampening T cell migration velocity (Fig. EV5F). TRPML1 activation also reverted reduced myosin IIA activation imposed by inhibition of VPS34 (Figs. 4F and EV5G). Lastly, a knock-down of TRPML1 with shRNA in human CD8^+^ T cell blasts reduced the migratory speed compared to a scrambled control (Fig. 4G). The knock-down was confirmed by Western blot and qPCR (Fig. EV5H).

From these experiments, a molecular framework emerged, where VPS34–PIKfyve derived PI(3,5)P_2_ was regulating TRPML1 mediated lysosomal Ca^2+^-efflux, thereby triggering the calmodulin–MLCK pathway and hence activation of myosin IIA.

### Regulation of amoeboid migration is a conserved function of the VPS34–PIKfyve axis

**Figure 5:**
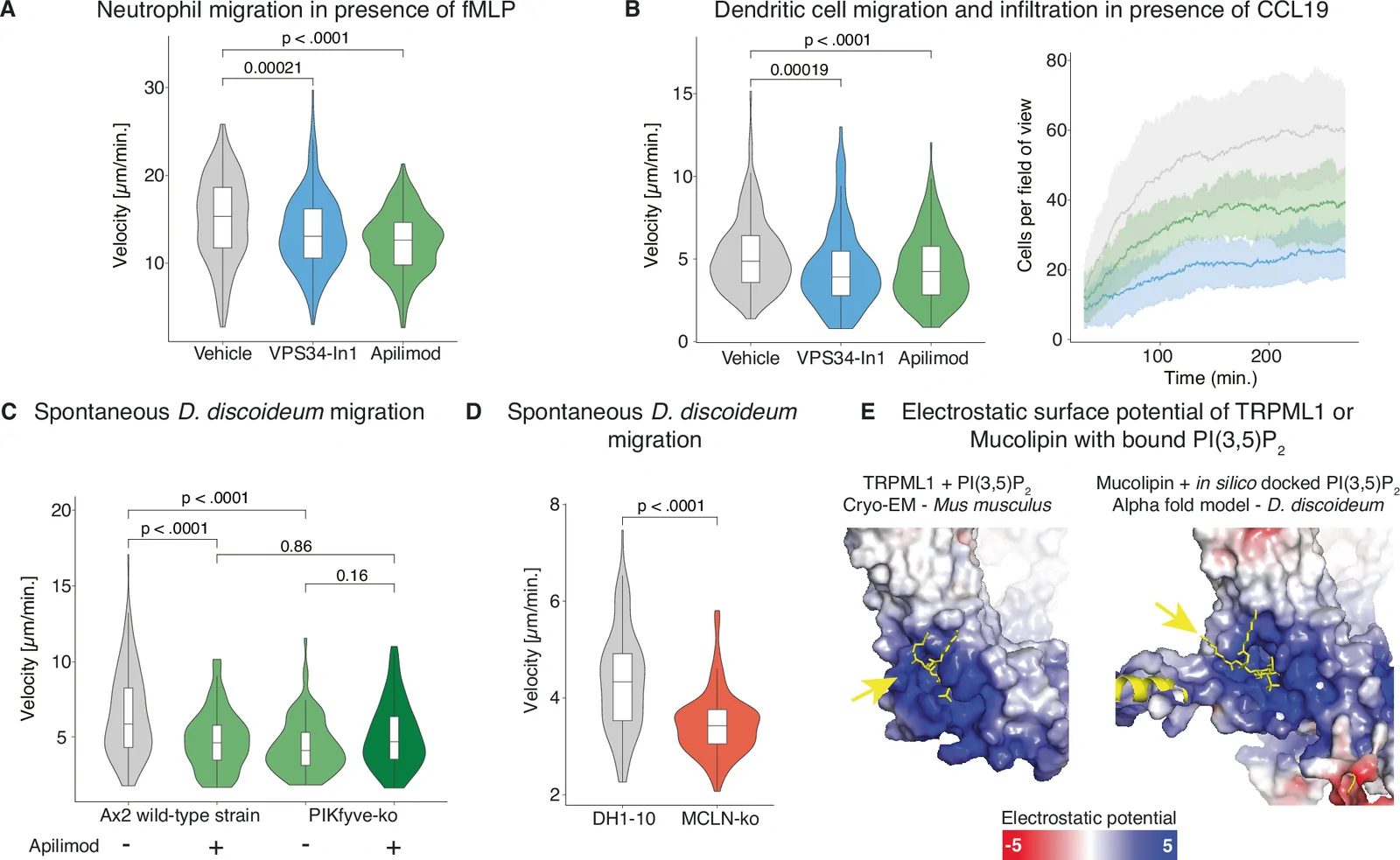
Regulation of amoeboid migration is a conserved function of the VPS34–PIKfyve axis. (**A**) Velocity of HL-60 derived neutrophils treated with vehicle control (*n* = 214), VPS34-In1 (*n* = 243), and Apilimod (*n* = 194), migrating in presence of the chemokine fMLP. (**B**) Left panel: Velocity of DCs migrating in CCL19, exposed to vehicle control (*n* = 412), VPS34-In1 (*n* = 104), Apilimod (*n* = 205). Right panel: Mean and standard deviation of number of cells detected per fields of view (*n* = 6 per condition) infiltrating under the agarose matrix. (**C**) Velocity of *D. discoideum* wild-type (Ax2) cells treated with vehicle control (*n* = 124) or Apilimod (*n* = 87), and PIKfyve-orthologue ko cells treated with vehicle control (*n* = 91) or Apilimod (*n* = 74). (**D**) Velocity of DH1-10 (wild-type strain, *n* = 113) and mucolipin-ko (MCLP-ko, *n* = 114) *D. discoideum* cells. (**E**) Left panel: Experimentally determined structure of TRPML1 (public domain) with bound PI(3,5)P_2_ published by Gan et al, 2022; Right panel: Predicted structure (public domain) of Mucolipin with PI(3,5)P_2_ docked in silico. The structures are aligned and superimposed with electrostatic potential. (**A**–**D**) Representative results of ≥3 independent experiments. Wilcoxon rank-sum test (**A**, **B**, **D**), ART ANOVA (**C**). Box plots indicate the median (central line), first and third quartiles (box bounds; 25th and 75th percentiles), and the minimum and maximum values (whiskers). Source data are available online for this figure.

**Figure EV6:**
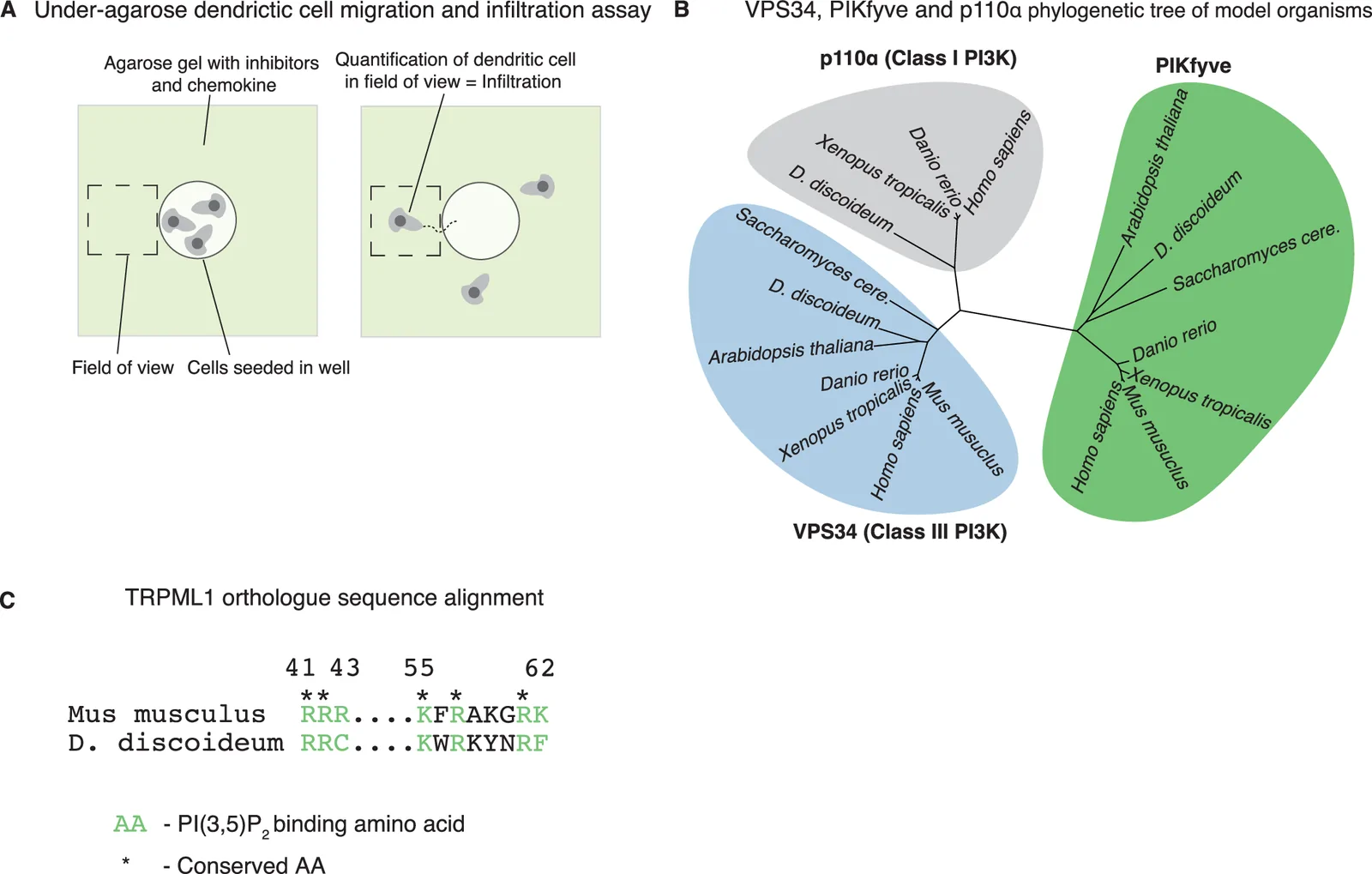
Regulation of amoeboid migration is a conserved function of the VPS34–PIKfyve axis. (**A**) Schematics of the migration and infiltration assay of DCs: Cells were added to the well in the agarose (containing inhibitors and CCL19, left panel). DCs crawled under the gel ( = infiltration) and the number of cells in the field of view was quantified (right panel). (**B**) Phylogenetic analysis of the amino acid (AA) sequences of VPS34, PIKFYVE, and p110α (as an example for Class I PI3K). (**C**) Alignment of the PI(3,5)P_2_-binding protein region from *Mus musculus* TRPML1 and the *D. discoideum* orthologue. PI(3,5)P_2_-binding AAs are highlighted in green, conserved AAs indicated by an asterisk.

To explore whether the VPS34–PIKfyve system is involved in regulating amoeboid migration also in other cell types, HL-60-derived neutrophils and murine bone marrow-derived DCs were used. Both, neutrophils in the presence of fMLP and DCs exposed to CCL19 migrated slower when blocking either VPS34 or PIKfyve (Fig. 5A,B, left panel). Infiltration of DCs under an agarose matrix was likewise reduced when blocking VPS34 or PIKfyve (Figs. EV6A and  5B, right panel). These findings expanded the role of the VPS34–PIKfyve system in regulating velocity of amoeboid cell migration beyond T cells. Of note, VPS34 and PIKfyve orthologues are widely expressed across all eukaryotic kingdoms (Fig. EV6B) (Philippon et al, 2015). We thus considered the idea that the VPS34–PIKfyve → TRPML1 pathway could be a broadly conserved biological module regulating velocity of amoeboid cell migration. To test this notion, we used the social amoeba and professional phagocyte *D. discoideum*, a non-metazoan model organism that separated from the animals and fungi a billion years ago (Douzery et al, 2004). *D. discoideum* exhibit similar vesicular alterations upon PIKfyve inhibition as metazoan cells, and its migration is myosin II-dependent (Buckley et al, 2019; Yoshida and Soldati, 2006). Further, absence of the *D. discoideum* TRPML1 orthologue Mucolipin (MCLN) impairs Ca^2+^-release from endo-lysosomes (Lima et al, 2012). Indeed, spontaneous amoeboid migration of PIKfyve knock-out (ko) and Apilimod-treated wild-type (Ax2) *D. discoideum* cells was slower than that of respective control cells (Fig. 5C). Inhibition of PIKfyve with Apilimod did not alter the speed of migration among PIKfyve ko cells, arguing for the specificity of the inhibitor (Fig. 5C). Moreover, MCLN-deficient *D. discoideum* phenocopied PIKfyve deficiency (Fig. 5D). Supporting the notion that MCLN was the target of PI(3,5)P_2_ also in *D. discoideum* cells, the alpha-fold model of MCLN displayed a positively charged pocket similar to the PI(3,5)P_2_ binding pocket in the N-terminus of TRPML1 (Fig. 5E) (Gan et al, 2022; Dong et al, 2010). In line, 5 out of 7 amino acids in this poly-basic domain are conserved between TRPML1 and MCLN (Fig. EV6C), and in silico docking analyses suggested potential binding of PI(3,5)P_2_ to this pocket in MCLN (Fig. 5E). It hence is plausible that regulation of migration speed is an ancient function of the VP34–PIKfyve axis.

In all, these findings suggested an evolutionarily conserved mechanism in which PI(3,5)P_2_, generated by the VPS34–PIKfyve axis, regulates TRPML1 activity, thereby controlling the release of Ca^2+^ from lysosomes at the uropod of migrating cells—and hence velocity of amoeboid cell migration.

## Discussion

The VPS34–PIKfyve axis, in association with endo-lysosomes, was observed to localize to the uropod of migrating T cells. This lipid kinase pathway regulates retrograde actin flow by enabling Ca^2+^ efflux from endo-lysosomes through TRPML1 and thereby shaping myosin IIA activity. Controlling cell migration velocity via this molecular axis applies to various cell types, including *D. discoideum*, i.e., unicellular eukaryotic cells.

Precise positioning of lysosomes has previously been shown to be important for amoeboid migration, ensuring site-specific availability of Ca^2+^ (Bretou et al, 2017; Vestre et al, 2021). We now show that the VPS34–PIKfyve axis links Ca^2+^ efflux from endo-lysosomes via TRPML1 to activation of myosin IIA. It should be noted, however, that PI(3)P levels and VPS34 activity have broad cellular functions including vesicular homeostasis, autophagy, and energy metabolism (Courreges et al, 2024; Jaber et al, 2016; Yang et al, 2021; Yuan et al, 2013). It is thus conceivable that, beyond the herein proposed signaling axis, yet to defined effects arising from disruption of PI(3)P–PI(3,5)P_2_ homeostasis further impact amoeboid cell migration.

Irrespective, our data highlight the importance of interrogating ultrastructural dynamics and associated signaling pathways in regulating cell migration. Microtubules and the microtubule-organizing center play a critical role in shaping the uropod and overall ultrastructure of amoeboid migrating cells. In T cells, for example, mitochondria are coupled to microtubules via Miro-1, which enables their relocation to the uropod upon chemokine signaling (Morlino et al, 2014). In an analogous manner, endo-lysosomes connected to microtubules by Arl8B or RILP/Rab7 have been shown to be re-positioned within cells depending on nutrient availability (Korolchuk et al, 2011; Scerra et al, 2022). Rab7b emerges as a potential co-modifier of the VPS34–PIKfyve axis in regulating amoeboid cell migration by tethering myosin II to lysosomes, thereby enabling its activation through TRPML1 (Vestre et al, 2021; Borg et al, 2014). Manipulating these adaptor proteins will help clarify how microtubule-dependent organelle positioning confines signaling to the uropod, and how this spatial regulation relates to amoeboid migration.

At the uropod, mitochondria, endo-lysosomes, and the ER reside in close vicinity. Intriguingly, membrane contact sites physically connecting these organelles jointly regulate Ca^2+^ dynamics and provide microdomains for local signaling (Burgoyne et al, 2015; Atakpa et al, 2018; Peng et al, 2020; Bartok et al, 2019). Whether such contact sites selectively form as an ultrastructural adaptation in migrating cells and, if so, their biologic roles, remains to be investigated.

In our experiments with T cells, chemokine-induced F-actin formation was unaffected by VPS34–PIKfyve activity. Notably, in neutrophils and cancer cells, PIKfyve has been shown to stimulate activity of the GTPases Rac1/2, which control actin polymerization (Faroudi et al, 2010; Dayam et al, 2017; Oppelt et al, 2014). It will be interesting to explore whether VPS34 and PIKfyve also control Rac1/2 in T cells and, if so, what the molecular and functional consequences of Rac1/2 activity are.

VPS34 and PIKfyve orthologues are universally expressed in eukaryotes and their appearance is known to predate the emergence of amoeboid cell migration (Philippon et al, 2015; Shisheva, 2008). Roles previously assigned to the VPS34–PIKfyve axis are regulation of endocytic trafficking and of autophagy (Burke et al, 2023). The herein described role in governing the speed of amoeboid cell migration not only in vertebrate immune cells but also in *D. discoideum*, indicates early functional assimilation of the VPS34–PIKfyve kinase system to also support this cellular process. Across taxa, F-actin polymerization and cortical actin-network contraction by myosin IIA lie at the center of amoeboid locomotion (Fritz-Laylin et al, 2017; Fritz-Laylin and Titus, 2023). Our study thus implicates regulation of myosin IIA activation by lysosomal Ca^2+^ via the VPS34–PIKfyve–TRPML1 axis as an evolutionary conserved regulator of this basic cellular process.

In all, our work identified a conserved regulatory node linking generation of lipid-mediators on endo-lysosomes with the efflux of Ca^2+^ from these organelles, thereby governing the speed of amoeboid cell migration.

## Methods

**Table:** Reagents and tools table

Reagent/resource	Reference or source	Identifier or catalog number
**Experimental models**		
C57BL/6NCrl	Bred in a specific-pathogen-free facility at the University of Basel	MGI:2683688
Peripheral Blood Mononuclear Cells from healthy, consenting donors	Blood Donor Center, University of Basel	
HL-60 cells	DSMZ	ACC 3
*D. discoideum* Ax2 WT strain	Soldati Laboratory, University of Geneva; Buckley et al, 2019	
*D. discoideum* Ax2 PIKfyve-ko strain	Soldati Laboratory, University of Geneva; Buckley et al, 2019	
*D. discoideum* DH1-10 WT strain	Cosson Laboratory, University of Geneva; Lima et al, 2012	
*D. discoideum* DH1-10 Mucolipin-ko strain	Cosson Laboratory, University of Geneva; Lima et al, 2012	
CD8 iCre: C57BL/6-Tg(Cd8a-cre)1Itan/J	Bred in a specific-pathogen-free facility at the University of Cambridge	MGI:6726258
Catalytically dead VPS34 flox: Pik3c3tm1Bvan	Bred in a specific-pathogen-free facility at the University of Cambridge; Courreges et al, 2024	MGI:6780732
**Recombinant DNA**		
pEGFP-2xFYVE plasmid	Addgene	140047
**Antibodies**		
Ultra-LEAF™ Purified anti-mouse CD3ε Antibody	BioLegend	100331
Ultra-LEAF™ Purified anti-mouse CD28 Antibody	BioLegend	1002116
Anti-Rab7	Abcam	ab137029
Anti-Lamp1	Abcam	ab25245
Anti-Phospho Myosin Light Chain 2 (Ser19	Cell Signaling Technology	3671
Anti-PI3 Kinase Class III	Cell Signaling Technology	4263
Anti-β-Actin	Cell Signaling Technology	3700
Anti-Rabbit IgG HRP	Cell Signaling Technology	7074
Anti-Mouse IgG HRP	Cell Signaling Technology	7076
Anti-Phospho-p70 S6 Kinase (Thr389)	Flow cytometry	9234
Anti-TRPML1	Alamone Labs	ACC-081
Anti-Rabbit IgG	Bio-Rad	170-6515
Anti-Rat IgG	Bio-Rad	5204-2504
Anti-α-Tubulin	Bio-Rad	MCA777G
Anti-Rabbit IgG AF647	Invitrogen	A-21244
Anti-Rat IgG AF488	Invitrogen	A-11006
**Oligonucleotides and other sequence-based reagents**		
*PIKfyve* targeting guide RNAs	Synthego	Sequences listed in “Methods”
*MCOLN1* and *SHDA* primers	Microsynth	Sequences listed in Table 6
**Chemicals, enzymes and other reagents**		
RPMI1640 (containing 25 mM HEPES and L-Glut)	Gibco	52400-025
Heat-inactivated fetal calf serum	Gibco	10270
GlutaMax	Gibco	35050-038
β-Mercaptoethanol	Gibco	31350-010
Penicillin and Streptomycin	Gibco	15140-122
EasySep™ Mouse Naive CD8^+^ T Cell Isolation Kit	Stemcell	19858
CD8a^+^ T Cell Isolation Kit, mouse	Miltenyi Biotec	130-104-075
Recombinant murine IL-7	Peprotech	217-17
Recombinant human IL-2	Bio-Techne Sales Corp.	202-IL-050
Recombinant murine GM-CSF	BioLegend	576306
LPS	Lucerna Chem	abx082480
CD8 MicroBeads, human	Miltenyi Biotec	130-045-201
ImmunoCult™ Human CD3/CD28 T Cell Activator	Stemcell	10971
Polybren	Sigma-Aldrich	TR-1003-G
shRNA (3 + 1) lentivirus targeting VPS34	VectorBuilder	Sequences listed in Materials and Methods
shRNA (3 + 1) lentivirus targeting TRPML1	VectorBuilder	Sequences listed in Materials and Methods
Recombinant Cas9	IDT	1081058
P3 Primary Cell 96-well Nucleofector® Kit	Lonza	V4SP-3096
Recombinant human IL-7	Peprotech	200-07
Recombinant human IL-15	Peprotech	200-15
PI(3,5)P_2_-diC_16_	Echelon Biosciences	P-3516
fatty-acid-free BSA	Sigma-Aldrich	A7030
PQR620	Wymann Laboratory, University of Basel	
VPS34-In1	Selleckchem	S7980
SAR405	EMD Millipore	533063
Apilimod	MedChem Express	HY-14644A
S(-4)’-nitro-Blebbistatin	Cayman Chemical	24171
ML-7	MedChem Express	HY-15417
Nocodazole	Sigma-Aldrich	M1404
MK6-83	Sigma-Aldrich	SLM1509
BAPTA-AM	MedChem Express	HY-100545
NucBlue™ Live ReadyProbes™ Reagent (Hoechst 33342)	ThermoFisher	R37605
LysoTracker Deep Red	Invitrogen	L12492
Calbryte 520 AM	AAT Bioquest	20650
µ-Slide 8-well high Glass Bottom slides	Ibidi	80807
Human plasma fibronectin	EMD Millipore	FC010
Ultra-pure agarose	Thermo Scientific	16500100
Phenol-red free RPMI	Gibco	11835-063
10x HBSS	Gibco	14065056
Recombinant human CXCL12	Preprotech	300-28 A
Recombinant murine CCL19	Preprotech	250-27B
CCL19-Dy649P1	Legler Laboratory, Institute of Cell Biology and Immunology Thurgau	
fMLP	Sigma-Aldrich	F3506
Falcon™ Bacteriological Petri Dishes with Lid	Falcon	10720052
HL5	Formedium	HLG0101
µ-Slide 4-well	Ibidi	80426
poly(L-lysine)-PEG	SuSoS	PLL(20)-g[3.5]- PEG(2)
8-well removable cell culture dishes	ibidi	80841
Poly D-Lysine	Milipore	A-003-E
#1.5H coverslip	ibidi	10811
ProLong Gold Antifade with DNA Stain DAPI	Invitrogen	P36931
RIPA buffer	ThermoFisher	89901
Protease inhibitors	Roche	05892970001
Phosphatase inhibitors	Roche	04906845001
BCA Protein Assay Kit	ThermoFisher	23225
4x Laemmli buffer	Bio-Rad	1610747
4-20% precast gradient gels	Bio-Rad	1704158
Protein page ruler	ThermoFisher	26620
0.2 µm nitrocellulose membranes	Bio-Rad	1704158
Chemiluminescent substrate	ThermoFisher	34580
4–12% precast gradient gel	Invitrogen	NP0321/NP0323
Nitrocellulose membranes	Invitrogen	IB301002
RNeasy Mini Kit	Qiagen	74104
GoScript™ Reverse Transcriptase Kit	Promega	A5003
QIAamp DNA Kit	Qiagen	56304
QIAquick PCR Purification Kit	Qiagen	28104
AF-488 phalloidin	Invitrogen	R37110
Zombie Aqua™ Fixable Viability Kit	BioLegend	423101
Transcription Factor Staining Buffer Set	Invitrogen	00-5523-00
Glutaraldehyde	Sigma-Aldrich	G5882
Paraformaldehyde	Science Services	E15710
PIPES buffer	Sigma-Aldrich	P6757
Green malachite	Sigma-Aldrich	32745
Low melt agarose	Roth	6351.1
Cacodylate buffer	Electron Microscopy Sciences	12300
Osmium-ferricyanide	Electron Microscopy Sciences	19100
K3Fe(III)(CN)6	Electron Microscopy Sciences	20150
Thiocarbohydrazide	Sigma-Aldrich	223220
Uranyl acetate	Electron Microscopy Sciences	22400
Durcupan	Electron Microscopy Sciences	14040
CellTrace Violet	Invitrogen	C34557
CellTrace Far Red	Invitrogen	C34564
**Software**		
Sanger Sequence Assembly Software	DNASTAR	https://www.dnastar.com/workflows/sanger-sequence-assembly/
ICE Analysis	Synthego	https://ice.editco.bio/#/
Ilastik	Berg et al	https://www.ilastik.org
FIJI	Schindelin et al, 2012	https://fiji.sc
TrackMate	Ershov et al, 2022	
Huygens	Scientific Volume Imaging	
Python		https://www.python.org
Jupyter Notebook		https://jupyter.org
AICSImageIO	GitHub	https://github.com/AllenCellModeling/aicsimageio
Napari	Sofroniew et al, 2025	
scikit-image	van der Walt et al, 2014	
NumPy	Harris et al, 2020	
FlowJo	BD	
Mega (Version 11.0.13)	Tamura et al, 2021	
PyMol	Schrödinger	https://www.pymol.org
Adaptive Poisson-Boltzmann Solver (APBS) plug-in	Jurrus et al, 2018	
HADDOCK 2.4	Honorato et al, 2021; van Zundert et al, 2016	
R (Version 4.1.2)		https://www.r-project.org
R-Studio	Poist	https://posit.co/download/rstudio-desktop/
BioRender.com	BioRender	https://biorender.com
**Other**		
4D-Nucleofector	Lonza	AFF-1002B
Nikon Ti microscope	Nikon	
Nikon Ti2 microscope	Nikon	
LED SpectraIII	Lumencor	
CSU-W1 spinning disk	Yokogawa	
LightHUBplus	Omicron	
Crest V3 spinning disk	CrestOptics	
Celesta solid-state laser	Lumencor	
CFI Plan Apo Lambda 20x air (NA: 0.75)	Nikon	
CFI Plan Apo Lambda 40x air (NA: 0.95)	Nikon	
CFI Plan Apo VC 60x water (NA: 0.95)	Nikon	
CFI Plan Apo Lambda 100x oil (NA: 1.45)	Nikon	
Stage-top incubator	LIS, Switzerland or OkoLab, Italy	
Prime 95B	Photometrics	
ImagEM X2	Hamamatsu	
DMI8 AF6000LX	LEICA	
Evolve 512 EMCCD	Teledyne Photometrics	
NeoPlan L 40X Corr CS (NA: 0.45)	LEICA	
Trans-blot turbo system	Bio-Rad	
Gel Doc system	Bio-Rad	
iBlot 1 dry blotting system	Invitrogen	
CytoFlex	Beckman Coulter	
FEI Helios NanoLab 650	FEI	
ZEISS Crossbeam 550 serial block face	Zeiss	
Aurora flow cytometer	Cytek	

### Methods and protocols

#### Cell isolation and culture

Cells and cell lines were cultured in RPMI1640 (containing 25 mM HEPES and L-Glut; Gibco) + 10% heat-inactivated fetal calf serum (hiFCS; Gibco) + GlutaMax (diluted 100×; Gibco) + 50 µM β-Mercaptoethanol (Gibco) + 100 U/ml penicillin and streptomycin (Gibco), referred to throughout the text as *complete medium*.

Naive murine CD8^+^ T cells were isolated from the spleens and lymph nodes (inguinal, axillary, and cervical) from C57BL/6NCrl mice with a negative magnetic isolation kit, according to the manufacturer’s instructions (Stemcell, Miltenyi Biotec). Mice of the age 6–25 weeks of both genders were used for the experiments. They were bred and housed in a specific-pathogen-free facility at the University of Basel. All animal experiments adhered to the guidelines of local veterinary authorities and were approved under the license numbers 2915_25054, 36623_3231 (Switzerland) and PP2364616 (UK). Isolated murine T cells were cultured in 10 ng/ml recombinant murine IL-7 (Peprotech) in complete medium and used up to 5 days after isolation. Murine T cells blasts were generated by plating naive T cells on anti-CD3 (coated at 5 µg/ml for 14 h at 4 °C or 4 h at 37 °C in PBS; BioLegend) with 1 µg/ml soluble anti-CD28 (BioLegend) and 100 U/ml of IL-2 (Bio-Techne Sales Corp.). After 1 day of activation, cells were daily resuspended in fresh 100 U/ml of IL-2 medium. If the medium turned bright yellow after 24 h, the cells were split 1:2.

HL-60 cells (a gift from Jürg Schwaller, University of Basel; DSMZ) were maintained in complete medium at a density of 2 × 10^5^–1.5 × 10^6^ cells/ml, split every 2–3 days and tested for Mycoplasma. Neutrophil differentiation was induced by adding 1.3% (v/v) DMSO into complete medium. Differentiated cells were used 5–7 days after DMSO addition. The cell line was not authenticated.

For DC differentiation, bone marrow was harvested from femurs and tibias of C57BL/6NCrl mice, and 2 × 10^6^ cells were plated in 10 cm Petri-dishes with 10 ml of complete medium containing 10 ng/ml GM-CSF (BioLegend). At day 3 and 6 fresh medium with GM-CSF was added. On day 8, the floating fraction of cells (immature DCs) was harvested and frozen in 90% hiFCS + 10% DMSO and stored in liquid nitrogen. Frozen cells were thawed in a water bath at 37 °C, added to prewarmed complete medium, and centrifuged at 200 × *g* for 5 min. The thawed cells were resuspended in complete medium and 10 ng/ml GM-CSF and 100 ng/ml LPS (Lucerna Chem) and matured for 14 h for migration experiments.

#### Human CD8^+^ T cell isolation, culture, shRNA-mediated gene silencing, and CRISPR-Cas9 gene-editing

Peripheral Blood Mononuclear Cells (PBMCs) were isolated by density centrifugation from buffy coats of healthy, consenting donors (Blood Donor Center, University of Basel, male and female, 18–65 years old). CD8^+^ T cells were isolated using anti-CD8 microbeads, following the manufacturer’s instructions (Miltenyi Biotec). In total, 2.5 × 10^6^ T cells were activated for 2 days with CD3/CD28-complexes (25 µl/ml, Stemcell) and 100 U/ml of IL-2. The cells were then split into two 500 µl samples and treated with 100 µl of lentivirus solution. Each solution contained 30 µl of scrambled or 10 µl VPS34-targeting shRNA per target sequence to a total of 30 µl with 6 µg/ml Polybren (Sigma-Aldrich) in complete medium. The cells were spinoculated for 60 min at 1000 × *g* at 30 °C. On day 3 after activation, the medium was replaced with complete medium containing 300 U/ml of IL-2. On day 6 post-activation, we sorted the cells for GFP^+^ expression using a FACSMelody Cell Sorter (BD). VPS34-kd and scrambled control cells were expanded and maintained in complete medium containing 300 U/ml of IL-2 for up to two weeks before use in experiments. TRPML1-knock down and scrambled control cells were expanded for 6 days in IL-2 after sorting and then further cultured in 10 ng/ml IL-7 and 10 ng/ml IL-15. The day before migration experiments cells were transferred into R10 without any cytokines. The high-titer shRNA lentivirus against human VPS34 (shRNA 3 + 1 with EGFP; target sequences: GAGGCAAATATCCAGTTATAT, CCACGAGAGATCAGTTAAATA, GCTGGATAGATTGACATTTAG) or human TRPML1 (target sequences: TCGCTGCTGGTGAATTGATTC, GCCTGTTCTCGCTCATCAATG, CGCCGTCGTCTCAAATACTTT), along with a scrambled control was procured from VectorBuilder.

CRISPR-Cas9 knockout of PIKfyve, three guide RNAs (gRNA; AUGGGAGUCACUAUGGAUCG, ACUAGCAAUGAGCUAGUAGU, CGGAAUGUAAAAACUGACAC) with standard modifications targeting exon 40 in the catalytic domain were procured from Synthego. Separately, 50 pM of gRNA was mixed with 30 pM of Cas9 (IDT) to a volume of 2.6 µl and incubated for 10 min at RT. The Cas9-gRNA complexes were then mixed to a volume of 7.8 µl, combined with 5  × 10^6^ freshly isolated human CD8^+^ T cells in 20 µl of P3 electroporation buffer (Lonza) and electroporated with the EH115 program in a 4D-Nucleofector unit (Lonza). The cells were then transferred into prewarmed medium R10 containing 10 ng/ml IL-7 (Peprotech) and 10 ng/ml IL-15 (Peprotech) in a 48-well dish. One day post nucleofection, cells were activated as described above. 6 d after activation cells, were again cultured in R10 with 10 ng/ml IL-7 and 10 ng/ml IL-15. The day before migration experiments cells were transferred into R10 without any cytokines.

#### Treatment with inhibitors/agonists and dipalmitoyl PI(3,5)P_2_ supplementation

All inhibitor stock solutions were prepared in DMSO, with the same DMSO concentration applied for vehicle controls. Inhibitor concentrations and manufacturers are listed in Table 1. The compound PQR620 was synthesized in-house at the University of Basel’s Wymann Laboratory, following a published protocol (Rageot et al, 2018). Prior to experiments, cells were treated with inhibitors or vehicle control for 2 h. Exceptions were MK6-83 and Nocodazole, which were exclusively incorporated into the agarose gel for migration assays.

**Table 1:** Pharmacological inhibitors: targets, sources, and concentrations.

Name	Target	Manufacturer	Concentration
PQR620	mTORC1/2	Wymann Lab (University of Basel)	2 µM
VPS34-In1	VPS34	Selleckchem	2 µM
SAR405	VPS34	EMD Millipore	4 µM
Apilimod	PIKFYVE	MedChem Express	2 µM
S(-4)’-nitro-Blebbistatin	Non-muscle myosin II ATPases	Cayman Chemical	25 µM
ML-7	MLCK	MedChem Express	10 µM
Nocodazole	Microtubules	Sigma-Aldrich	1 µM
MK6-83	TRPML1	Sigma-Aldrich	1, 5, 10 µM
BAPTA-AM	Calcium	MedChem Express	20 µM

Pharmacological inhibitors used to perturb signaling pathways and cytoskeletal processes as indicated.

PI(3,5)P_2_-diC_16_ (Echelon Biosciences) was conjugated to fatty-acid-free BSA (Sigma-Aldrich) by incubating at 37 °C for 1 h. Subsequently, phenol-red free RMPI1640, preheated to 37 °C, was added to the fatty-free BSA to attain a concentration of 100 mg/ml, yielding a final stock concentration of 500 µM. Aliquots of this stock were stored at −80 °C.

#### Staining of live cells with fluorescent dyes

Nuclear staining was performed using 1 drop Hoechst 33342 (ThermoFisher; one drop per two ml of medium) per 2 ml of complete medium for 30 min. at 37 °C or if applicable in parallel with the drug treatment. For endo-lysosomal localization naive murine CD8^+^ T cells were additionally stained with 1 µM LysoTracker Deep Red (Invitrogen) and Hoechst 33342. For calcium imaging, T cells were stained with 2.5 µM Calbryte 520 AM (AAT Bioquest) in complete medium at 37 °C for 30 min and washed once in complete medium. Subsequently, a 2 h drug treatment was carried out in the presence of 1 µM LysoTracker Deep Red.

#### Under-agarose cell migration assay of live cells stained with fluorescent dyes

The under-agarose migration assay was adapted from a previously published method (Heit and Kubes, 2003). Ibidi µ-Slide 8-well high Glass Bottom slides (Ibidi) were coated with 150 µl of human plasma fibronectin (10 µg/ml; EMD Millipore) in PBS and incubated at 37 °C for 2 h. The slides were then rinsed twice with 400 µl PBS per well. To cast the 1.2% agarose gels, 0.48 g of ultra-pure agarose (Thermo Scientific) was mixed in 10 ml deionized water. Separately, 4 ml filtered hiFCS, 16 ml phenol-red free RPMI (Gibco), and 10 ml 2× HBSS (pH 7.2, from 10× HBSS stock; Gibco) were combined and heated to 60 °C. The agarose was heated to a boil for three times in a microwave, interspersed with mixing. The medium, HBSS, and FCS mixture were then added to the molten agarose solution, followed by gentle mixing. Each well received 400 µl of the agarose mixture and was left to solidify with a closed lid. When inhibitors were used, 100 µl of a 5× inhibitor solution in complete medium was added to the gel and incubated overnight at RT, sealed with parafilm. Before the migration assay, the medium solution was removed, and a well was created with a 3 mm biopsy puncher. Cells pretreated with inhibitors and stained with Hoechst 33342 (see above) were centrifuged at 200× *g* for 5 min, and resuspended in complete, phenol-red free, inhibitor-containing medium at a concentration of 1 × 10^7^ cells/ml. In total, 5.5 µl of the cell suspension was injected 2–3 mm away from the punched well using a micropipette with a gel-loading tip. All fluid was aspirated from the well, which was then filled with 20 µl of complete medium containing chemokines. For human CD8^+^ T cell blast migration assays, 2 µg/ml CXCL12 (Preprotech) was added, whereas for murine T cell migration assays, 2 µg/ml CCL19 (Peprotech) or 20 µM CCL19-Dy649P1 in complete medium was added in the punched well (Artinger et al, 2021). The dish was then transferred to the microscope. For DC experiments, the agarose gel was overlaid with 5× inhibitor/vehicle solution and 5× CCL19 solution (final concentration: 0.25 µg/ml) and cells were added to the well. For neutrophil migration assays, gels were overlaid with 5× inhibitor/vehicle solution and 5× fMLP (Sigma-Aldrich, final concentration at 10 nM). For endo-lysosomal localization in stained naive murine CD8^+^ T cells, images were acquired on a Nikon Ti equipped CSU-W1 spinning disk unit (see Tables 2 and 3) and a Hamamatsu ImagEM X2 camera at ×100 magnification. The excitation light intensity was set to 100% for all wavelengths. The camera gain was adjusted to keep a very short exposure time (50 ms), while avoiding saturation. Z-stack of chosen cells were acquired in steps of 120 nm.

**Table 2:** Microscopy modalities, systems, and experimental applications.

Experiment	Microscope modality	System	Objective	Assay/application	Sampling rate
Immunofluorescence of endo-lysosomes	Widefield	Nikon Ti2	×100	Fixed and mounted cells	Single images
Immunofluorescence of RLC phosphorylation			×40		
Assessment of retrograde actin flow	Spinning disk confocal	Nikon Ti or Ti2	×60, ×100	Modified under-agarose (see below)	1 fps
Live cell migration with inhibitors	Nikon Ti2	Nikon Ti2	×20	Under-agarose	20 or 30 s
Live cell imaging: T cell morphology	Widefield	Nikon Ti2	×40 and 1.5 optical zoom, ×100	Under-agarose	Single images
Live cell imaging: T cell with Lysosomal stains/CCL19-Dy649P1/2xFYVE-eGFP probe/calcium indicator	Widefield/Spinning disk confocal	Nikon Ti2	×100	Under-agarose	10 fps or single images

Overview of imaging modalities, microscope systems, and experimental conditions used for fixed and live-cell imaging.

**Table 3:** Microscopy system configurations and components.

Microscope	Spinning disk unit	Objective(s)	Camera	Light source	Dichroic mirrors and filters
Nikon Ti2	n/a	CFI Plan Apo Lambda ×20 (NA 0.75) CFI Plan Apo Lambda ×40 (NA 0.95) CFI Plan Apo Lambda ×100 (NA 1.45)	Photometrics Prime 95B	Spectra III (Lumencor) Excitation at 395, 475, 555, 635 and 730 nm	Filter cube: DM 408/504/581/667/762 EM 440/40, 520/21, 606/34, 694/34, 809/81
Nikon Ti	CSU-W1 (50 µm pinhole)	CFI Plan Apo VC ×60 WI (NA 1.2) CFI Plan Apo Lambda ×100 (NA 1.45)	Photometrics Prime 95B or Hamamatsu ImagEM X2	LightHUBplus (Omicron)	DM 405/488/568/647 EM 460/50 EM 525/50 EM 630/75 EM 700/75
Nikon Ti2	Crest V3 (50 µm pinholes with 250 µm spacing)	CFI Plan Apo VC ×60 WI (NA 1.2) CFI Plan Apo Lambda ×100 (NA 1.45)	Photometrics Prime 95B	Celesta (Lumencor) Excitation at 405, 477, 546, 640 and 749 nm	DM 421/491/567/659/776 EM 438/24 EM 511/20 EM 595/31 EM 685/40

Detailed specifications of microscope setups, including objectives, detectors, illumination sources, and filter sets.

#### Microscopy imaging modalities and analysis

Most imaging experiments (except *D. discoideum* migration assay) were conducted using Nikon Ti or Ti2 microscopes (Tables 2 and 3), equipped with a Prime 95B (25 mm, Photometrics) camera. The Nikon Ti2 widefield microscope employed an LED (SpectraIII, Lumencor), the Nikon Ti CSU-W1 spinning disk (Yokogawa) utilized a diode-pumped solid-state laser (LightHUBplus, Omicron) and the Nikon Ti2 Crest V3 spinning disk (CrestOptics) used a solid-state laser as a light source (Celesta, Lumencor). CFI Plan Apo Lambda objectives were used, with magnifications of ×20 air (NA: 0.75), ×40 air (NA: 0.95), ×100 oil (NA: 1.45), and ×60 water (NA: 1.2, Plan Apo VC). For live cell imaging, stage-top incubators (LIS, Switzerland, or OkoLab, Italy) maintained a constant temperature of 37 °C, 100% humidity (20 l/h airflow), and 5% CO_2_.

Cell morphology and fluorescence intensities were measured using FIJI (Schindelin et al, 2012). Regions of interest (ROI) were manually drawn in FIJI to obtain mean fluorescence intensities and shape descriptors. Each experiment involved analyzing at least three different fields of view per condition. Cell tracking, either for nuclei or brightfield images, was conducted using either Ilastik (with Pixel Classification followed by Tracking) or TrackMate in FIJI (Log detector; Simple LAP tracker with linking max distance: 20 microns, gap-closing max distance: 20 microns, gap-closing max frame gap: 2) (Berg et al, 2019; Ershov et al, 2022). Colocalization analysis was performed on cropped images containing only one single cell using Huygens (Scientific Volume Imaging).

#### Quantification of vesicle localisation

We developed a custom analysis workflow in Python for vesicle distribution analysis. This workflow depends on AICSImageIO, napari, pandas, scikit-image, Jupyter Notebook, and NumPy (Brown et al, 2025; Sofroniew et al, 2025; The pandas development team, 2024; van der Walt et al, 2014; Harris et al, 2020).

Briefly, before running the Python workflow, crops of individual cells were created and saved using Fiji. A Jupyter notebook was used to batch run the Python analysis workflow. Nuclei were segmented in the DAPI channel by Gaussian blurring (sigma = 3 in X, Y, and scaled for Z), Otsu thresholding and a fill holes operation. The vesicle channel was segmented by first median filtering of the channel (ball with 3 voxels diameter) and either Otsu or Yen thresholding (if not specified in the workflow parameters, the lower threshold of both was chosen). Clustered vesicles were then separated by a watershed transform and objects smaller than 0.015 µm^3^ were excluded. The output csv file of the workflow reports various object measurements (for nucleus and vesicles). In particular, vesicles have additional measurements: “2D area”, the vesicle’s maximum intensity projection area; “distance-NucSurface”, the shortest distance from the vesicle’s centroid to the nucleus surface; “distance-NucCentroid”, the distance between vesicle and nucleus centroids; “min_ves-ves-dist” and “max_ves-ves-dist”, the minimal and maximal distance between any vesicle centroid in the image; “angle”, the 2D projected angle between nucleus and vesicle centroids relative to the image *x* axis; “angleNorm2Mean”, the angle normalised to the average of all angles in the image; “angleNorm2Middle”, the angle normalised to half the minimum and maximum angle in the image.

#### *Dictyostelium discoideum* culture and migration assay

The *D. discoideum* strains used in this study are listed in Table 4. Cells were axenically grown in 10-cm Petri dishes (Falcon) at 22 °C in HL5 or HL5c medium (Formedium) supplemented with 100 U/ml penicillin and streptomycin (Gibco). At least 24 h before the migration experiments, all the cell lines were grown in HL5 medium. In total, 6 × 10^3^ cells were seeded in each well of a 4-well µ-slide (Ibidi) in HL5 medium and incubated 1 h at 22 °C. When necessary, 5 µM Apilimod (Table 1) or DMSO was added to the cells before the incubation. Time-lapse movies of 30 min with images taken at 30 s intervals were recorded with a widefield inverted LEICA DMi8 microscope equipped with an Evolve 512 EMCCD camera (Teledyne Photometrics), using a ×40 air objective with a 120 W mercury lamp (short arc lamp). Images were processed with the Trackmate Fiji plug-in.

**Table 4:** *Dictyostelium discoideum* strains.

Genotype	Background strain	Source/reference
Wild-type	Ax2	Buckley et al, 2019
PIKfyve-ko		
Wild-type	DH1-10	Gift from Pierre Cosson; Lima et al, 2012
Mucolipin-ko		

*Dictyostelium discoideum* strains used in this study, including genetic background and source information.

#### Assessment of retrograde actin flow

The assessment of retrograde actin flow was carried out using a modified under-agarose assay (as described above). The slides were first plasma cleaned and PEG-coated at 4 °C overnight with 200 µg/ml poly(L-lysine)-PEG (SuSoS) in PBS. The slide was washed three times with PBS and 400 µL of 1.2% agarose solution (without any serum). The gels were then treated overnight with 100 µL of 5× solutions of either the vehicle, VPS34-In1, or Apilimod in serum- and phenol-red free RPMI1640 containing 1.25 µg/ml CCL19. Subsequently, Lifeact T cell blasts were treated with either VPS34-In1 or Apilimod for 2 h and then injected under the agarose gel (as described above).

Actin flow videos were acquired at 1 frame per second (fps) for 30 s in the GFP channel (Ex.: 488 nm, Em.: 525; exposure time 50–100 ms) using an inverted spinning disk confocal (details above). The acquired images were then analyzed with FIJI: First, cells with visible actin spots were cropped and loaded into the KymographClear plugin with the average intensity projection (Mangeol et al, 2016). A line was then drawn through the cells, along the F-actin foci traces from the uropod to the leading edge, to generate a kymograph (with a line width of 5 pixels). The angle of a representative F-actin trace was analyzed, from which the speed was calculated by taking the tangent of the angle and scaling the result to µm/min. The tracking of F-actin foci was performed using Trackmate and the integrated LoG detector.

#### Immunofluorescence staining

Eight-well removable cell culture dishes (ibidi) were coated with 100 µg/ml Poly D-Lysine (Milipore) in PBS for 2 h at 37 °C and washed three times with PBS. Then, 1 × 10^5^ naive CD8^+^ T cells were plated per well, centrifuged for 30 s at 300×*g*, and treated with 0.5 µg/ml CCL19 and inhibitors according to the protocol. At the end of the treatment, prewarmed EM-grade paraformaldehyde was added directly to the medium to achieve a final concentration of 4%. The plate was then incubated for 15 min at 37 °C and washed once with PBS. Cells were permeabilized with 0.2% Triton-X in PBS for 15 min and blocked with 5% goat serum in PBS-T (blocking buffer) for 1 h. Primary antibodies were incubated overnight at 4 °C and secondary antibodies for 1 h in the dark at RT, with three intermittent washes with PBS-T. The antibodies were diluted in blocking buffer (Table 5). Finally, the slides were washed in PBS, mounted with a coverslip (#1.5H; ibidi) and ProLong Gold Antifade with DNA Stain DAPI (Invitrogen) and sealed the next day with nail polish.

**Table 5:** Antibodies: targets, sources, dilutions, and applications.

Antibody target	Manufacturer	Dilution	Application
Anti-Rab7	Abcam	1:1000	Immunofluorescence/western blot
Anti-Lamp1			
Anti-Phospho Myosin Light Chain 2 (Ser19)	Cell Signaling Technology	1:100	
Anti-PI3 Kinase Class III		1:1000	Western blot
Anti-β-actin		1:10,000	
Anti-rabbit IgG HRP			
Anti-mouse IgG HRP			
Anti-Phospho-p70 S6 Kinase (Thr389)		1:500	Flow cytometry
Anti-TRPML1	Alamone Labs	1:1000	Western blot
Anti-rabbit IgG	Bio-Rad	1:10,000	Western blot
Anti-rat IgG			
Anti-α-tubulin	Bio-Rad	1:10,000	Western blot
Anti-rabbit IgG AF647	Invitrogen	1:1000	Immunofluorescence/flow cytometry
Anti-rat IgG AF488			Immunofluorescence

Primary and secondary antibodies used for immunofluorescence, western blotting, and flow cytometry.

#### Immunoblotting

Human CD8^+^ T cell blasts were harvested, washed with ice-cold PBS, and then centrifuged at 500×*g* for 5 min. Cells were lysed in RIPA buffer (ThermoFisher) supplemented with protease inhibitors (Roche) and phosphatase inhibitors (Roche). Cell lysates were stored at −20 °C and cleared by centrifugation at 15,000× *g* for 10 min at 4 °C prior to use. Protein concentrations were determined with a BCA Protein Assay Kit (ThermoFisher). For each sample, 5 µg of protein per replicate was combined with 4× Laemmli buffer (Bio-Rad) and 10% 2-mercaptoethanol, and then denatured at 95 °C for 5 min. Protein samples were loaded into 4–20% precast gradient gels (Bio-Rad) alongside a page ruler (ThermoFisher), and then subjected to electrophoresis for 5 min at 80 mV, followed by 55 min at 100 mV. The separated proteins were transferred to 0.2 µm nitrocellulose membranes (Bio-Rad) using a Trans-blot turbo system (Bio-Rad) and blocked with 5% BSA in TBST for 1 h at room temperature (RT). Membranes were sliced at the 70 kDa mark and incubated overnight at 4 °C with either anti-VPS34 or anti-Actin antibodies (Table 5). Following three washes with TBST, membranes were incubated for 1 h at RT with horseradish peroxidase (HRP) conjugated secondary antibodies (Table 5). Membranes were then washed, incubated for 5 min with chemiluminescent substrate (ThermoFisher), and imaged on a gel doc system (Bio-Rad).

For detection of pRLC by Western blotting, murine T cell blasts were treated for 2 h with the inhibitors (VPS34-In1, Apilimod or vehicle) and then stimulated with 0.5 µg/ml CCL19 for 30 min. If appropriate, the chemokine stimulation coincided with 5 µM MK6-83 treatment. The cells were washed once in ice-cold PBS, resuspended in RIPA buffer, and immediately snap-frozen on dry ice. For each replicate, the same amount of protein was loaded onto a 4–12% precast gradient gel (Invitrogen) and transferred onto nitrocellulose membranes (Invitrogen) using an iBlot 1 dry blotting system (Invitrogen). Membranes were blocked as described above, sliced at the 30 kDa mark, and incubated for 3 days at 4 °C with either anti-phospho-Ser19 myosin light chain 2 or anti-α-tubulin antibodies (Table 5).

#### *MCOLN1* qPCR

Total RNA from LV-transduced, sorted cells was isolated following the manufacturer’s instructions using the RNeasy Mini Kit (Qiagen). Reverse transcription was performed with 100 ng of total RNA using the GoScript™ Reverse Transcriptase Kit (Promega) with random primers in a 20 µl reaction volume, and the cDNA was diluted 1:2 in nuclease-free H₂O. qPCR reactions were prepared using 1 µl of diluted cDNA, SYBR Green qPCR master mix, and the *MCOLN1* and *SDHA* primers listed in Table 6. The relative *MCOLN1* transcript levels in knockdown T cells were calculated using the 2^–ΔΔCt^ method, with SDHA as the housekeeping gene.

**Table 6:** PCR primer sequences.

Primer name	Sequence (5’–3’)
Fwd-*MCOLN1*	CGG ACT GCT ATA CCT TCA GCG T
Rev-*MCOLN1*	GGT GCT TAC ACT CCT GGA TGT G
Fwd-*SDHA*	GCA TTT GGC CTT TCT GAG GC
Rev-*SDHA*	CTC CAT GTT CCC CAG AGC AG
Fwd-*PIKfyve*	GGC CAG TGT ATT TCG GAA ACG
Rev-*PIKfyve*	GCT AAC AAT GTG CCA AGT GCT G

Primer sequences used for amplification of indicated genes.

#### PIKfyve sequencing

Genomic DNA was isolated from PIKfyve-KO and control cells using the QIAamp DNA Kit (Qiagen) 7 days after electroporation. The genomic region surrounding the gRNA binding sites was amplified using 150 ng gDNA as template and the PIKfyve primers listed in Table 6. The PCR product was run on an agarose gel and purified using the QIAquick PCR Purification Kit (Qiagen). A total of 180 ng of the purified PCR product was then premixed with primers and sent to Microsynth for sequencing. The sequencing results were analyzed using the Sanger Sequence Assembly Software (DNASTAR) and ICE Tool (Synthego) to determine the KO score.

#### Electroporation of 2xFYVE–eGFP plasmid into T cells

The pEGFP-2xFYVE plasmid, (a kind gift from Harald Stenmark; Addgene) electroporation was performed using a 4D-Nucleofector (Lonza) with the P3 Nucleofector kit (Lonza). Naive murine CD8^+^ T cells (2 × 10^6^) were washed twice with PBS and resuspended in 20 µl of P3 electroporation buffer containing 1 ng of the plasmid. The cell suspension was then added to an electroporation cuvette strip. Immediately after electroporation, 130 µl of prewarmed complete medium with 20 ng/ml IL-7 was added to the cuvette, and the cells were allowed to rest for 10 min at 37 °C. The cell suspension was mixed, centrifuged at 200×*g* for 5 min, and resuspended in 1 ml of prewarmed complete medium with 20 ng/ml IL-7. The cells were used for imaging 6 h after electroporation until the following day.

#### Flow cytometric analysis of F-actin polymerization, live/dead staining, S6K phosphorylation, calcium levels, and CCL19 uptake

For F-actin staining, 7.5 × 10^4^ naive murine CD8^+^ T cells were treated with either a vehicle, VPS34-In1, or Apilimod and then stimulated for 1 min with 0.5 µg/ml CCL19. The cells were fixed directly in the medium with 4% PFA for 15 min, washed in PBS, and permeabilized with 0.2% Triton-X in PBS for 15 min. F-actin was stained with AF-488 phalloidin (1 drop in 4 ml FACS buffer; Invitrogen) for 30 min, washed thrice, and then acquired in FACS buffer (PBS + 5% BSA).

To assess the effect of VPS34 or PIKFYVE inhibition on murine CD8^+^ T cell blast viability, cells were treated with inhibitors for 6 h and then stained with Zombie Aqua (BioLegend) following the manufacturer’s instructions.

S6K phosphorylation was measured by intracellular staining in murine CD8^+^ T cells activated for 24 h with plate-bound anti-mouse CD3 (coated at 5 µg/ml for 14 h at 4 °C in PBS; BioLegend) and 1 µg/ml soluble anti-CD28 (BioLegend). Cells were stained for live/dead cells, fixed, and permeabilized for 15 min, followed by overnight staining with a primary anti-pS6K antibody at 4 °C and one hour of secondary antibody staining at RT (Table 5). This staining procedure was carried out with the Transcription Factor Staining Buffer Set (Invitrogen).

Calcium levels were measured in Calbryte stained T cells resuspended in complete medium treated with 5 µM MK6-83 or vehicle control for 5 min at 37 °C. At least three technical replicates were acquired directly in the medium for 60 s at a flow rate 30 µl/min.

CCL19 uptake in naive CD8^+^ T cells was measured by adding 20 nM of CCL19-Dy649P1 to the cell culture medium for 0, 30, or 90 min. The cells were then subjected to live/dead staining and washed in FACS buffer. All samples were acquired with a CytoFlex (Beckman Coulter) and analyzed using Flowjo (BD).

#### Preparation and acquisition of electron microscopy samples

A total of 1 × 10^7^ cells were fixed by adding a 2× concentrated EM fixative (comprising a final solution of 2.5% glutaraldehyde (Sigma-Aldrich) and 2.0% paraformaldehyde (Science Services) in 100 mM PIPES buffer (pH 7.2; Sigma-Aldrich) to the cell culture medium (1:1) for 20 min at RT. After removing the fixative, it was replaced with a 1× fixative containing 0.01% green malachite (Sigma-Aldrich) for 2 h on ice. The cells were rinsed six times with PIPES buffer before being embedded in a 2% low melt agarose (Roth). Embedded cell pellets were washed five times for 3 min each in cacodylate buffer (0.15 M cacodylate with 4 mM CaCl2; Electron Microscopy Sciences) before being post-fixed with osmium-ferricyanide (2% OsO_4_, Electron Microscopy Sciences; 1% K3Fe(III)(CN)6, Electron Microscopy Sciences; in cacodylate buffer) for 30 min on ice. After five washes with ddH_2_O for 3 min each, cell pellets were immersed in a thiocarbohydrazide (TCH, Sigma-Aldrich) solution for 30 min. Subsequently, cell pellets were treated with 1% OsO_4_ in water for 30 min at RT, washed five times, and immersed in 1% uranyl acetate (Electron Microscopy Sciences) overnight at 4 °C. On the following day, cells were washed five times for 3 min. each in ddH_2_0 and treated with Walton’s lead aspartate (0.66% lead nitrate in 0.4% aspartic acid solution) for 1 h. After another wash, the cells underwent dehydration using an ethanol series (25%, 50%, 75%, 90%, and 2×100%) for 5 min each. The cell pellets were then infiltrated with Durcupan (Electron Microscopy Sciences) using an ethanol-Durcupan gradient (20%, 50%, 70%, 90%, and 2×100%) for 3 min each in the microwave. This was followed by a 30 min immersion in a 1:1 Ethanol:Durcupan resin and a subsequent overnight immersion in 100% Durcupan solution. The next day, cells were immersed in freshly prepared resin for 1 h. Resin blocks were polymerized at 60 °C for 3 days. The polymerized samples were mounted on SEM stubs and imaged on either an FEI Helios NanoLab 650 (courtesy of NanoImaging Lab Basel; acquired pixel size: 8 × 8 ×  16 nm) or a ZEISS Crossbeam 550 serial block face (courtesy of the Electron Microscopy Core Facility of EMBL Heidelberg; acquired pixel size: 8 × 8 × 40 nm). Image stacks were aligned using the SIFT module available on FIJI for analysis (Lowe, 2004).

#### In vivo T cell homing assay

For adoptive T cell transfer, isolated murine CD8^+^ T cells were initially rested overnight and then divided into two groups. One group was labeled with 250 nM CellTrace Violet (CTV), while the other group was labeled with 1000 nM CellTrace Far Red (CTFR) (Invitrogen) for 15 min in PBS at 37 °C. Subsequently, the stained cells were separately treated with either 1:1000 DMSO or 2 µM Apilimod for 2 h at 37 °C, followed by washing. The cells were then combined in a 1:1 ratio in PBS. CD8^+^ T cells with wild-type VPS34 (isolated from CD8a-cre^+/−^, VPS34-Exon21^wt/wt^ animals) were labeled with CTV, while those with catalytic-dead VPS34 (CD8a-cre^+/−^, VPS34-Exon21^flox/flox^ animals) were labeled with CTFR, and both were mixed in a 1:1 ratio. Generation and characterization of the VPS34-Exon21 flox model was described previously (Courreges et al, 2024). CD8a-cre and VPS34-Exon21-flox mice were bred and housed in a specific-pathogen-free facility at the University of Cambridge.

The labels were switched for each respective repetition experiment. For injection, a total of 3 × 10^6^ cells (1.5 × 10^6^ per condition) were administered into the tail vein of recipient mice. After 1 h spleens and lymph nodes (inguinal, axillary, and cervical) were harvested, and single-cell suspensions were obtained by passing the organs through a 70 µm cell strainer. Samples collected from spleens and organs were subjected to red blood cell lysis, followed by a single wash in FCS buffer and fixation with 4% PFA. The ratio of transferred cells was quantified using a CytoFlex (Beckman Coulter) or Aurora flow cytometer (Cytek). Subsequently, the homing index was calculated by determining the ratio of CTV-labeled cells to CTFR-labeled cells in comparison to the input ratio. The relative abundance of Apilimod-treated cells or cd-VPS34 T cells was normalized to their internal controls per recipient mouse.

#### Sequence alignment and phylogenetic analysis

The amino acid sequences were accessed through the public domain (UniProt; Table 7) (The UniProt Consortium, 2023). The Mega (Version 11.0.13) software was used to perform alignments with the MUSCLE algorithm and the phylogenetic analysis with the maximum likelihood method (Tamura et al, 2021).

**Table 7:** Protein sequences: organisms and accession numbers.

Organism	Gene/*orthologue*	Accession #
*Arabidopsis thaliana*	VPS34	P42339
	FAB1A/*PIKfyve*	Q0WUR5
*Danio rerio*	PIKfyve	B2KTE3
	p110α	F1QAD7
	VPS34	F1Q9F3
*D. discoideum*	PI5K3/*PIKfyve*	B0G126
	pikA/*p110α*	P54673
	pikE/*VPS34*	P54676
	Mucolipin/*TRPML1*	Q54Y0
*Homo sapiens*	PIKfyve	Q9Y2I7
	p110α	P42336
	VPS34	Q8NEB9
*Mus musculus*	Pikfyve/*PIKfyve*	Q9Z1T6
	p110α	P42337
	VPS34	Q6PF93
	TRPML1	Q99J21
*Saccharomyces cerevisiae* (Saccharomyces cere.)	VPS34	P22543
	Fab1/*PIKfyve*	P34756
*Xenopus tropicalis*	PIKfyve	A0A6I8Q3Y5
	p110α	B0BLW6
	VPS34	F6ZM84

Proteins and their orthologues across species, with corresponding accession numbers used for phylogenetic sequence analysis.

#### Protein structure analysis and in silico docking

Protein structures for TRPML1 bound to PI(3,5)P_2_ (*Mus musculus; experimentally determined*; UniProt Entry: Q99J21, Structure identifier: 7sq7) and Mucolipin (*D. discoideum; Alphafold model*; UniProt Entry: Q54EY0, Structure identifier: AF-Q54EY0) were sourced through the public domain (UniProt, AlphaFold) (The UniProt Consortium, 2023; Jumper et al, 2021; Varadi et al, 2022). Structures were aligned in PyMol and electrostatic potential was computed with the Adaptive Poisson-Boltzmann Solver (APBS) plug-in (Jurrus et al, 2018). The in silico docking was performed with high ambiguity-driven protein-protein docking (HADDOCK 2.4) (van Zundert et al, 2016; Honorato et al, 2021). The predicted mucolipin and PI(3,5)P_2_ structures were inputted and the PI(3,5)P_2_ binding AAs were added as interacting residues. The output model was then loaded into PyMol and aligned with the TRPML1 structure.

#### Statistical analysis

Data processing, statistical analyses, and visualization were conducted in R (version 4.1.2) using the packages tidyverse, ARTool, and ggpubr (Wickham et al, 2019; Kay et al, 2025; Kassambara et al, 2025). Cell migration parameters (velocity, directedness) were calculated from the tracking output (described) using a custom-written script in R. Short tracks and non-moving tracks were excluded from the analysis. For directedness analysis cell tracks were analyzed for the same time span (> 10 min). The same filters were used for all conditions across the experiments. Error bars represent standard deviation. Violin plots depict the distribution of individual data points, with overlaid box plots indicating the median (central line), first and third quartiles (box edges), and data range (whiskers). Pairwise comparisons were conducted using two-tailed *t* tests or Wilcoxon rank-sum tests, selected based on data normality (Shapiro–Wilk test) and sample size. Two-factor analyses were performed using two-way ANOVA followed by Fisher’s Least Significant Difference (LSD) test, or aligned rank transform ANOVA. The applied statistical test is specified in each figure legend. No sample size estimate or blinding was used in this study.

#### Graphics

Some of the graphics were created with BioRender.com.

## Supplementary information

Peer Review File

Source data Fig. 1

Source data Fig. 2

Source data Fig. 3

Source data Fig. 4

Source data Fig. 5

Expanded View Figures

## Data Availability

The analysis workflow to quantify vesicle localization can be accessed on GitHub (https://github.com/loicsauteur/vesicle-analysis, version 0.1.1). The source data of this paper are collected in the following database record: biostudies:S-SCDT-10_1038-S44319-026-00861-x.
