# Supplementary material for: A conserved VPS34-PIKfyve-TRPML1-myosin II axis regulates the speed of amoeboid cell migration
Source: EMBO Rep. 2026 Jul 7;27(16):4721–50. doi: 10.1038/s44319-026-00861-x (PMC13503823; doi:10.1038/s44319-026-00861-x)
Supplement: Supplementary file 1 [file 44319_2026_861_MOESM1_ESM.pdf]

EMBO reports

A conserved VPS34-PIKfyve-TRPML1-Myosin II axis regulates the speed of amoeboid cell migration

Philippe Dehio, Céline Michard, Juan Carlos Yam-Puc, Adrià-Arnau Martí i Líndez, Anett Jandke, Gunhild Unterstab, Lucien Fabre, Loic Sauteur, Marc Artinger, Daniel Legler, Michael Sixt, Thorsten Schaefer, Matthias Wymann, Klaus Okkenhaug, Thierry Soldati, Matthias Mehling, and Christoph Hess

Corresponding author(s): Christoph Hess (ch818@cam.ac.uk)

Review Timeline:

Submission Date:	1st Dec 25
Editorial Decision:	27th Mar 26
Revision Received:	29th Apr 26
Editorial Decision:	13th May 26
Revision Received:	29th May 26
Accepted:	12th Jun 26

Editor: Martina Rembold

Transaction Report: This manuscript was submitted to EMBO Reports after peer review at another journal.

(Note: With the exception of the correction of typographical or spelling errors that could be a source of ambiguity, letters and reports are not edited. Depending on transfer agreements, referee reports obtained elsewhere may or may not be included in this compilation. Referee reports are anonymous unless the Referee chooses to sign their reports.)

General remarks

We thank the Reviewers for their thorough and constructive evaluation of our manuscript. In response to the concerns raised, we have substantially expanded the study experimentally. Specifically, we worked on three key aspects: (*i*) robust quantification of endo-lysosomal polarization, (*ii*) additional genetic validation of our findings, and (*iii*) the dependency of VPS34–PIKfyve regulated cell migration on chemokine signaling.

Ad (*i*) To strengthen the quantitative basis of our study, we now provide additional electron microscopy and 3D live-cell spinning-disk imaging data, analyzed using a purpose developed, automated pipeline for unbiased quantification of endo-lysosomal polarization.

Ad (*ii*) To corroborate key results obtained using pharmacological inhibitors we complemented these using genetic approaches. Specifically, we performed TRPML1 knockdown and PIKfyve knockout experiments in primary T cells. Insight gained from these experiments is consistent with our previous inhibitor-based data and aligns with published work that examined the function of TRPML1 in other cell types.

Ad (*iii*) We addressed the reviewers' question regarding a possible requirement of chemokine signaling for VPS34-PIkfyve regulated cell migration by analyzing polarized T cell blasts in the presence *vs.* absence of chemokines. Notably, VPS34–PIKfyve inhibition reduced the speed of migration also in absence of chemokine signaling.

In addition to these key issues, we have incorporated, as recommended, additional control experiments; refined statistical analyses, and improved data presentation and amended the discussion of key points.

We feel that, thanks to the additional experiments, refined analyses and textual changes proposed by our Reviewers, the manuscript has substantially improved.

Below we address each concern in a *Point-by-point* manner.

Point-by-point reply

Reviewer #1: The work "An evolutionary-conserved VPS34-PIKfyve-TRPML1-Myosin II axis regulates the speed of amoeboid cell migration" by Dehio et al. builds up on previous knowledge about the role of TRPML1 in amoeboid cell migration and on its activation by PI(3,5)P2. Dehio et al. investigate now whether the VPS34-PIKfyve axis is a conserved mechanism for the production of PI(3,5)P2 necessary to activate TRPML1, and therefore for controlling amoeboid cell migration. The work is generally well performed and shows an interesting role of the VPS34-PIKfyve axis in the activation of TRPML1 for amoeboid cell motility. However, there are fundamental issues with data presentation and interpretation. Hence, the data in the current form are not always convincing or not able to support the authors' claims and conclusions. Specific points are listed below:

> We are pleased that this Reviewer feels our work is describing an interesting role for the VPS34-PIKfyve axis in activating of TRPML1 for amoeboid cell motility.

Major points:
1) Data should be presented to show experimental variability. Therefore, data should not be pooled and statistical analysis should be performed between independent experiments (e.g. S. J. Lord , K. B. Velle, R. D. Mullins, L. K. Fritz-Laylin. J Cell Biol 2020). The number of mice used (and number of cells from each animal) should also be indicated. In addition:
- The description of statistical analysis is missing in the methods.
- Explanation about the error bars is missing in almost all figure legends.
- The y-axis on bar charts should start with zero to avoid misleading.

> As suggested, all data that previously were pooled have now been replaced to **show the results from individual experiments**, and the y-axes of the bar charts were adjusted accordingly (please see **new Figure 2J-K and Figure S3D-E**). Importantly, the results obtained from pooled and non-pooled analyses are consistent.
>
> In addition, error bars in all figures now indicate standard deviation, and this is explicitly stated in the figure legends and relevant sections of the text. All box plots with the whisker's indicating the data range (overlayed on violin plots) are standard format and are described in the description of the statical analyses. **A detailed description of the statistical analyses employed has now been added** in the revised *Methods* section.

2) The introduction section is poorly structured and it is not clear what led the authors to investigate the role of lysosomes as signaling hubs in controlling amoeboid migration of T cells. It is also unclear why the result section starts by assessing the localization of organelles in resting vs. chemokine-activated naïve murine CD8+ T cells, without giving any background on the rationale behind this experiment. The introduction should be re-written to clearly explain the background to the study and hypothesis tested by the authors.

> Following this advice, we partially re-wrote and re-structured the introduction. We start by providing the background to the work performed, and follow this by first stating the knowledge gap identified and then frame our overarching hypothesis. We close with a brief statement rationalizing expansion of the work to other cell types. We hope these

changes are satisfactory. However, should the reviewers or editors recommend additional amendments or changes, we are happy to further refine based on their input.

3) It is stated in Fig. 1 legend that" Endo-lysosomes decorated with VPS34-PIKfyve relocate to the uropod of polarized T cells". However, it is unclear how the analysis of the microscopy images has been performed to reach that conclusion. Are the analyses shown in and 1F performed on single stacks only? An important aspect to consider in this type of analysis is that endo-lysosomes can be distributed differently in different focal planes, especially in non-flat cells as T cells. Also, it is difficult to conclude from the micrographs shown in Figure 1A that only the stimulated cell is polarized (=organelles grouped at one side of the cell) as also in the non-stimulated cell most of the organelles seem to be concentrated at one side.

To address this important point, we **substantially revised Figure 1** by adding new datasets and re-analyzing existing data as follows:

- **New Figure 1A:** We analyzed FIB-SEM z-stacks of seven cells per condition (vehicle and CCL19), assessing the distribution of endo-lysosomes throughout the cell. Representative z-plane images from three individual cells are now displayed:

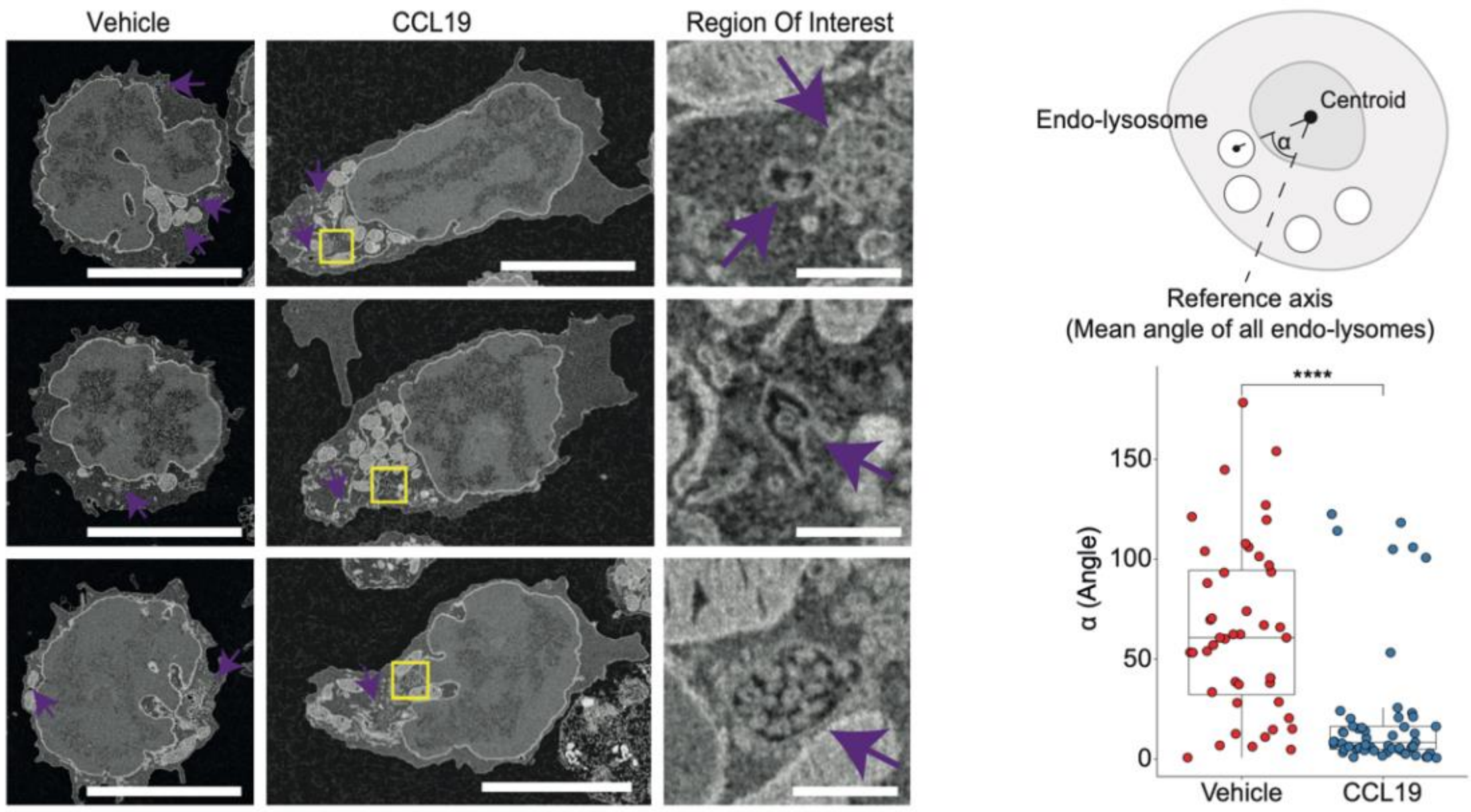

- **New Figure 1B:** We now present z-maximum-intensity projections, and quantify endo-lysosomal polarization using a purpose-developed automated analysis pipeline (described in the Methods section in detail):

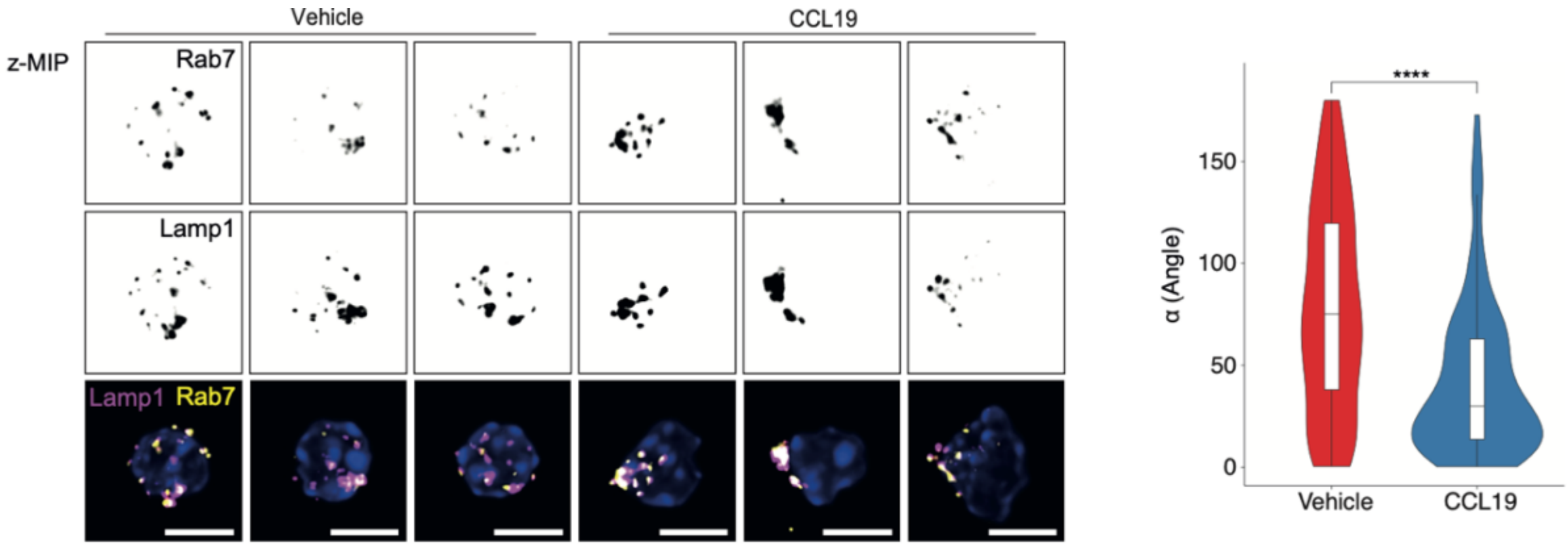

- **New Figure 1C:** We replaced the previous Figure 1C (as well as Figure S1B) with new data. Lysotracker- and Hoechst-stained naïve CD8$^+$ T cells were imaged in 3D using spinning-disk microscopy, and endo-lysosomal polarization was quantified automatically. z- and z/y-maximum-intensity projections are displayed to illustrate the spatial distribution of endo-lysosomes:

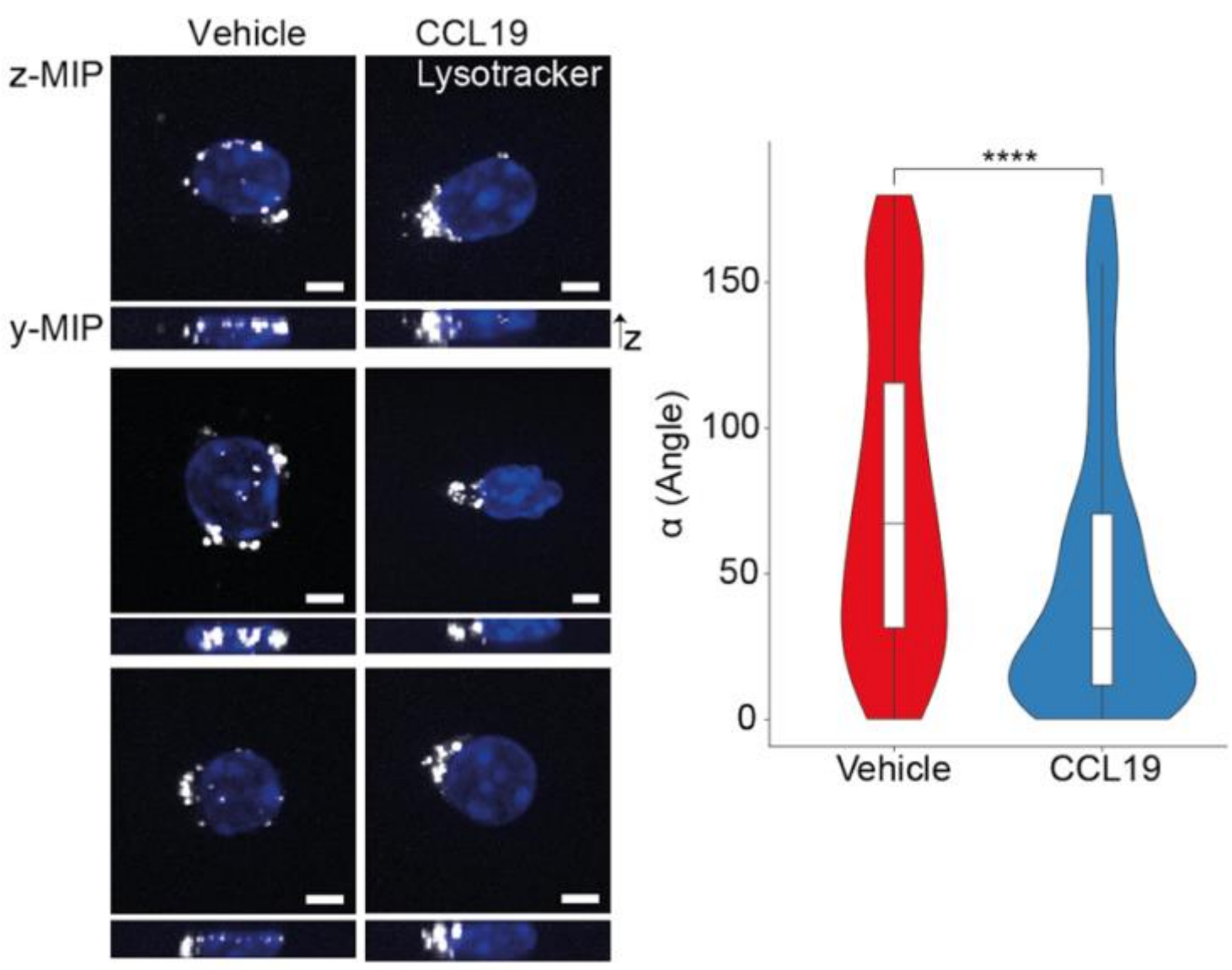

- **Old Figure 1F**: The former Figure 1F has been replaced with updated data, **now presented as Figure 2B**. Here, we imaged T cells expressing 2xFYVE-eGFP and stained with lysotracker using 3D spinning-disk microscopy:

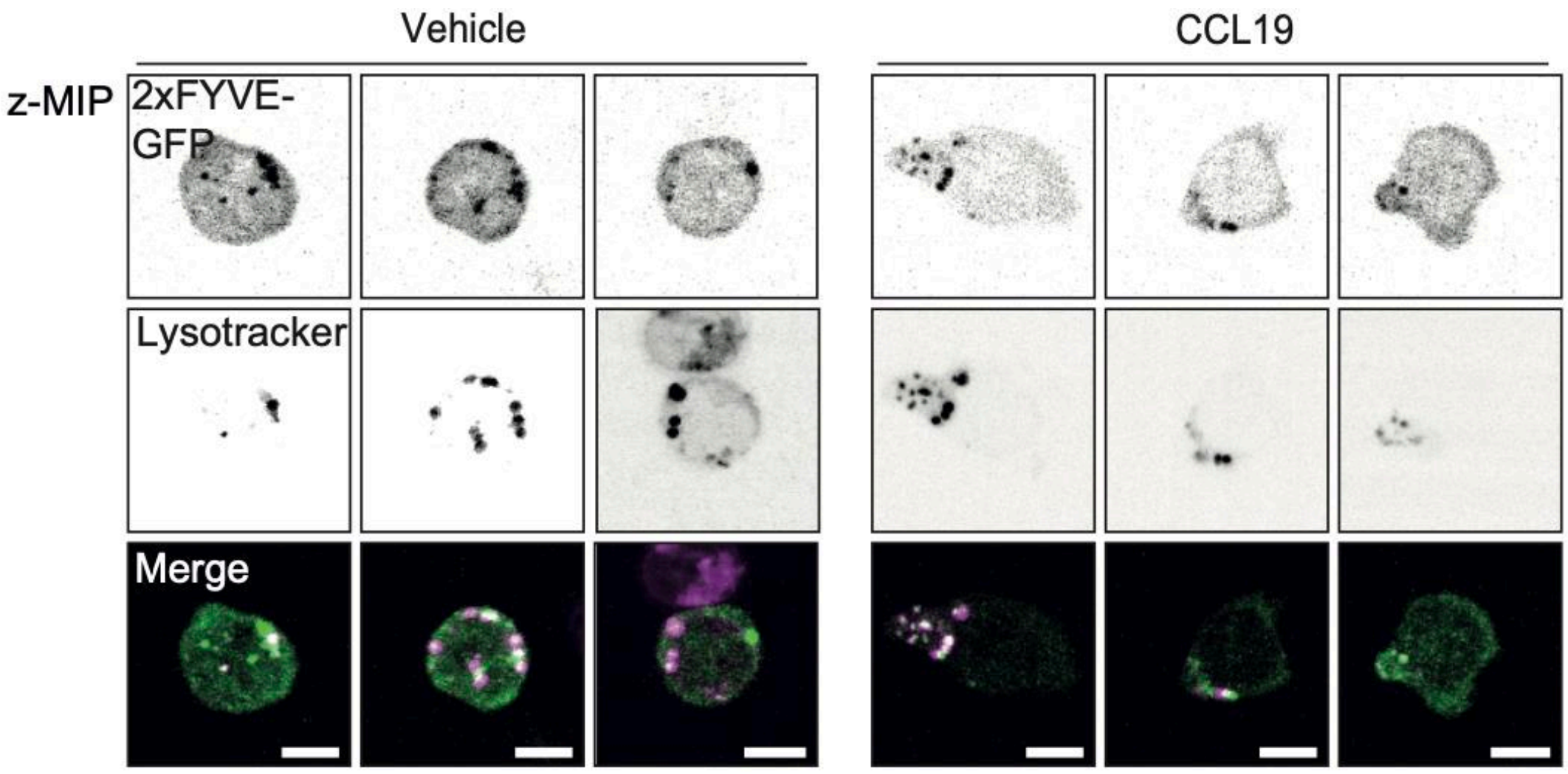

We hope that this extensive experimental effort is aligning with the Reviewers expectation.

4) The increased colocalization between Rab7 and Lamp1 measured upon T cell stimulation is not sufficient to conclude that chemokine exposure induces endo-lysosomal maturation (as stated at page 4 and 6). To demonstrate an involvement in endo-lysosomal maturation, specific experiments targeting this aspect (e.g. lysosomal degradation ability, Rab5/Rab7 conversion, etc.) should be performed. Lamp1 is present not only on lysosomes but also on late endosomes (e.g. C. S. Pillay, E. Elliott and C. Dennison, Biochem. J., 2002, 363, 417-429). This should be corrected at page 4. Furthermore, the colocalization detected between Rab7 and Lamp1 is unexpectedly low and may be a consequence of poor recognition by Rab7 antibody (antibodies against Rab proteins are notoriously very poor for detecting endogenous levels of the proteins) or low expression. The increased colocalization of Rab7 with Lamp1 upon T cell stimulation could be therefore a consequence of other factors, as for example increased expression of Rab7, rather than of endo-lysosomal maturation.

We thank the Reviewer for pointing this out. Since these data are somewhat immature – as correctly identified by our peer– and of no relevance to the biologic question we aimed to interrogate, we chose to remove it. There is also no mention anymore of lysosomal maturation in the text. Our focus was redirected toward the unbiased quantification of vesicle polarization, using the pipeline presented in the **new Figures 1B and 1C**. Consistent with our previous observations, $Rab7^{+}$ vesicles clustered upon chemokine treatment. Of note, we carefully chose a Rab7 antibody that is widely used (>100 publications) and has been knockout-validated.

5) The authors did not detect an effect on migration upon inhibition of mTOR and speculate that this could be a consequence of low mTOR activity in naïve T cells (page 4). However, naïve T cells migration has been previously reported to decrease in presence of the mTOR inhibitor rapamycin (e.g. Haas R. et al, Plos Biology, 2015). Furthermore, it has been previously shown that mTOR activates VPS34 (e.g. Munson M. J. et al, EMBO J, 2015), so this should be properly investigated or at least clarified.

The Reviewer is highlighting an important issue. In our reading of the literature, the relationship between mTOR activity and amoeboid cell migration, including T cell migration, remains somewhat inconclusive and is certainly complex. In the study referenced in our manuscript (Haas et al., 2015), the authors used overnight rapamycin treatments, which may alter chemokine receptor expression (Sinclair et al., 2008), and can have additional knock-on effects. In contrast, we used the highly selective mTORC1 inhibitor, PQR620, in short term exposure assays, which could account for differing experimental outcomes. Notably, mTOR activity has been reported to be dispensable for dendritic cell migration (Nakatani et al., 2021), aligning with our observations in T cells.

As the Reviewer correctly points out, mTOR signaling may influence VPS34 activity, but is not strictly required for its function (Munson et al., 2015). The interplay between mTOR and VPS34 is sophisticated and context-dependent: VPS34-derived PI(3)P can activate mTOR (Hong et al., 2017), while nutrient starvation inactivates mTOR and, conversely, activates VPS34 (Yuan et al., 2013). To account for the uncertainty around this topic, and given that mTOR signaling was not the focus of this study, we have restructured this section of the manuscript. The **relevant data are now included in Supplementary Figure S2I–J**, and they are discussed in the context of the potential interplay between mTOR and the VPS34–PIKfyve axis. We hope this addresses this reviewers concern, and remain very open for additional suggestions.

6) It is unclear how the experiment in Figure 1F was performed: were the cells stimulated? If not, what did it cause their polarization? Were the non-polarized cells viable? How was the analysis performed?

We apologize for insufficient clarity. The cells used for the data displayed in Figure 1F of the original submission were indeed stimulated with CCL19. In the revised manuscript, we replaced the previous Figure 1F with **new spinning-disk imaging data, now presented in Figure 2B (please see above, your Major point 3)**, with accurate labeling of the treatment conditions.

7) Page 9: the authors write that "these experiments identified the lipid kinase pathway PI-PI(3)P-PI(3,5)P2, governed by VPS34 and PIKfyve, as a modifier of endo-lysosomal turnover at the uropod of T cells". This conclusion should be toned-down as there are no data showing the involvement in endo-lysosomal turnover at the uropod. It is only shown an increase in lysotracker signal in VPS34In-1 and in Apilimod-treated naïve murine CD8+ T cells, which could be a consequence of defective pathways that have not been investigated (e.g. the transport from endosomes to lysosomes, lysosome acidification, autophagy, etc.). Furthermore, as the authors also mention in the text, it is already known that VPS34-PIKfyve have a role in endo-lysosomal homeostasis.

We agree with the Reviewer and have toned down this statement accordingly, now reading as follows: "*Together these experiments identified lipid kinase activity along the PI→PI(3)P→PI(3,5)P2 pathway, governed by VPS34 and PIKfyve at the uropod of T cells, to determine endo-lysosomal volume and velocity of migrating CD8⁺ T cells, while not affecting chemotactic directedness.*"

8) Why does the mean of the speed of human CD8+ T cell blasts migrating in CXCL12 gradient (control cells) vary so much between different experiments (e.g. in suppl. Fig. 2F control cells have a mean speed of 6-7 µm/min while in Fig. 2E the control cells have a mean of 11-12 µm/min)? Is this due to a difference in cell viability?

Thank you for raising this issue. The study of primary human cells (as opposed to e. g. clonal cells or cell lines) is inherently prone to variability in experimental outcomes. In our specific case, primary non-clonal cells were pre-activated and subsequently propagated with interleukin-2 for up to three weeks. This requirement further introduced inter-experimental variability, jointly underpinning the differences in migration speed observed in Figure 2E (**now Figure 2F**) and Figure S2F (**now Figure S2H**).

To experimentally address the reviewers' concern regarding the slower overall migration speeds in Figure S2H, we repeated this experiment with an independent batch of T cell blasts. The **results were more consistent with our previously reported speed ranges** and confirmed the same functional phenotype. **For consistency, in the revised manuscript, we have replaced the original data with these new experiments**:

Experiment displayed in original draft:

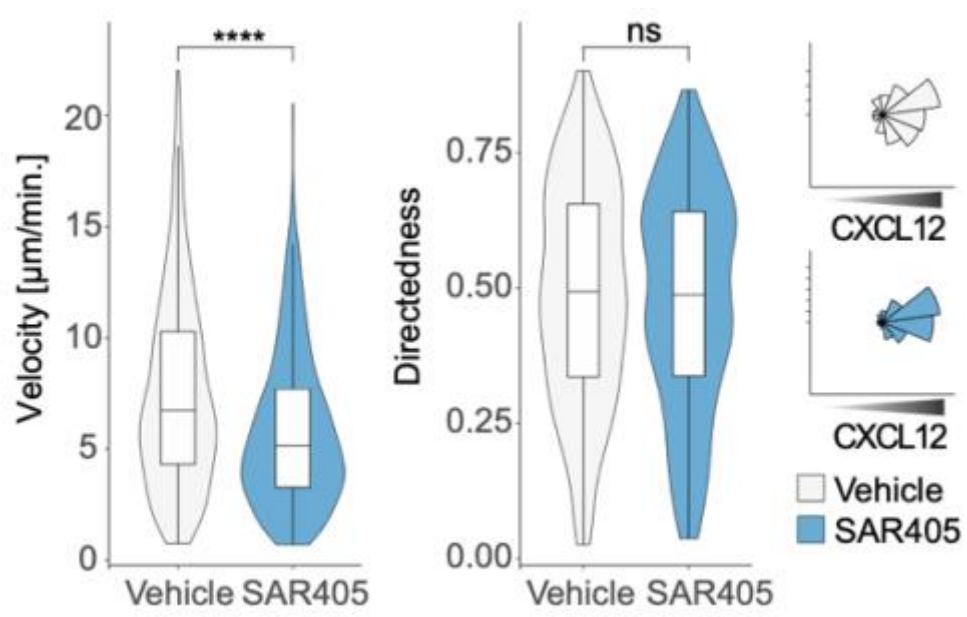

Repetition experiment:

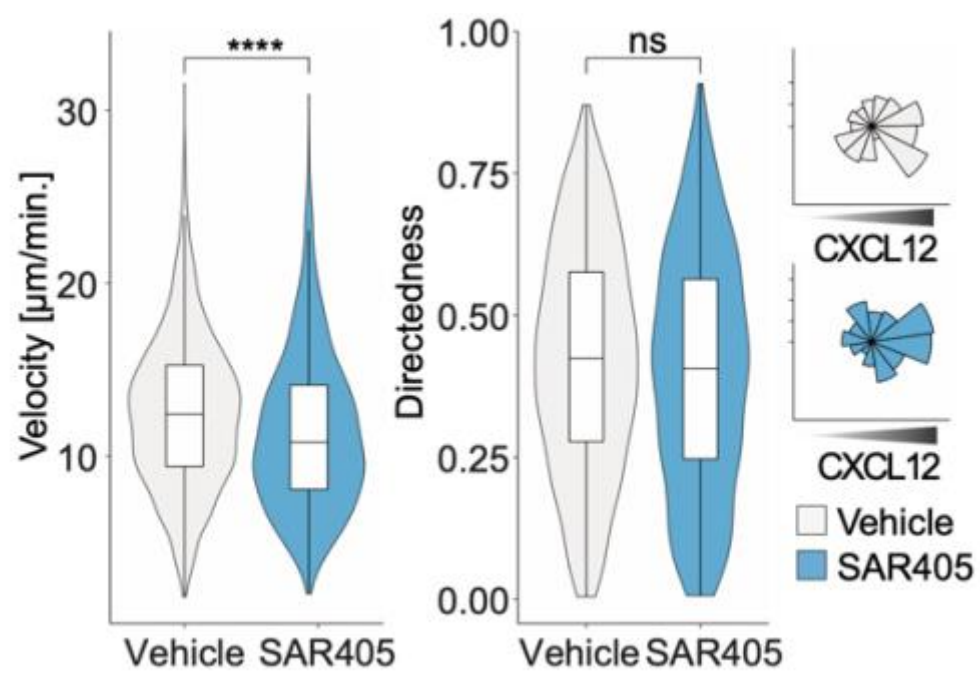

We are very happy to provide all data from all experimental repetitions, along with an estimate of inter-experimental variability, if this is felt to be useful.

9) Fig. 3F and 4F: myosin phosphorylation should be confirmed by WB.

To confirm the findings shown in Figures 3F and 4F of our original submission, CCL19-exposed murine T cell blasts were treated with vehicle, VPS34-In1, or Apilimod. We selected this cell system to obtain sufficient material for probing myosin RLC phosphorylation through Western blot analysis. Due to significant variability in signal strength, these experiments were technically challenging – which is particularly evident in the MK6-83 rescue data.

The results shown below are based on multiple biological replicates and numerous independent WBs, and were **added to the revised manuscript as Supplementary Figures S4B and S5G**:

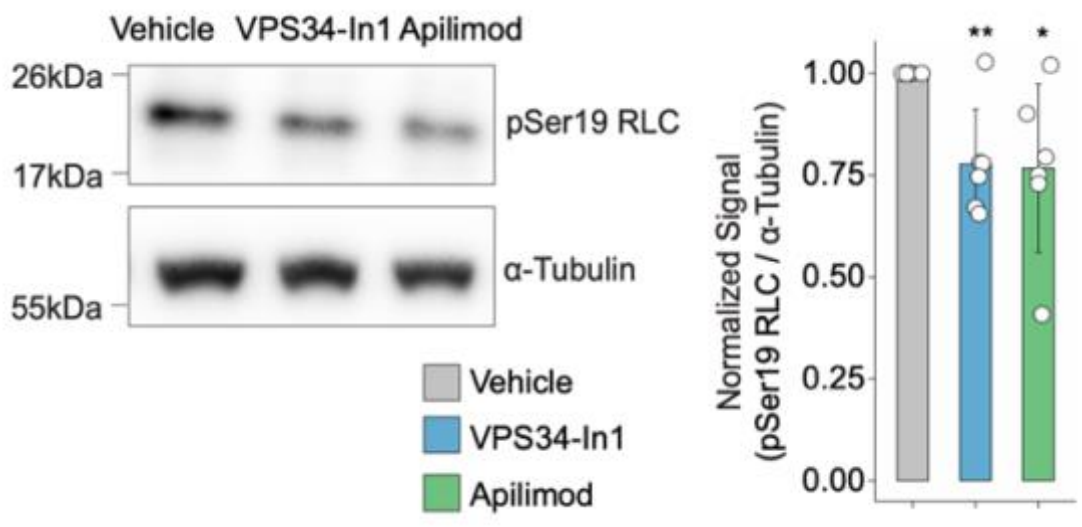

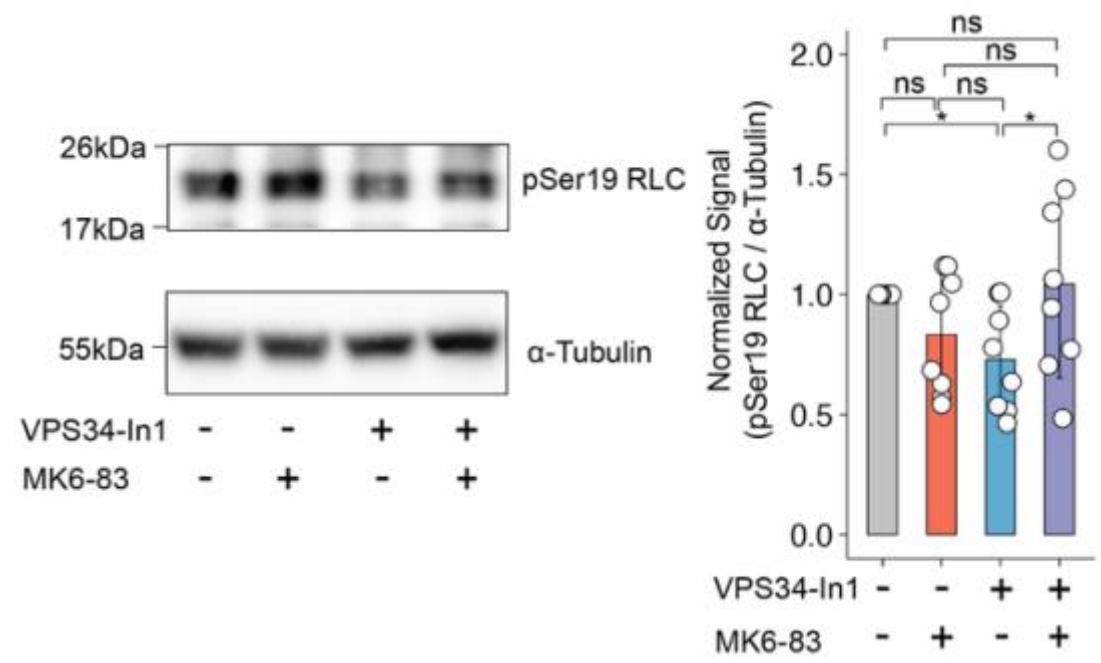

10) Fig.4A e D: Lysotracker and calcium signal do not seem to overlap in some of the images shown - which also have a poor resolution. This should be improved, as poor resolution affects colocalization analysis.

The detailed scrutiny of this Reviewer is truly appreciated, and we agree that these analyses do have limit resolution and colocalization accuracy. Please note that T cells migrate rapidly under agarose, and latency between image acquisitions, as well as the widefield microscopy mode used in these experiments, inherently limit resolution and colocalization accuracy, as the Reviewer correctly noted. To address this, **we re-analyzed the data using an orthogonal approach, manually segmenting the lysosomal region and measuring the intensity of the Ca^{2+} dye as a proxy for endo-lysosomal Ca^{2+} retention**. This analysis indicates increased endo-lysosomal Ca^{2+} levels upon VPS34 or PIKfyve inhibition, which are reversible by TRPML1 agonism. These **new data** have been incorporated in the revised manuscript as **Supplementary Figures S5A** and **S5D**:

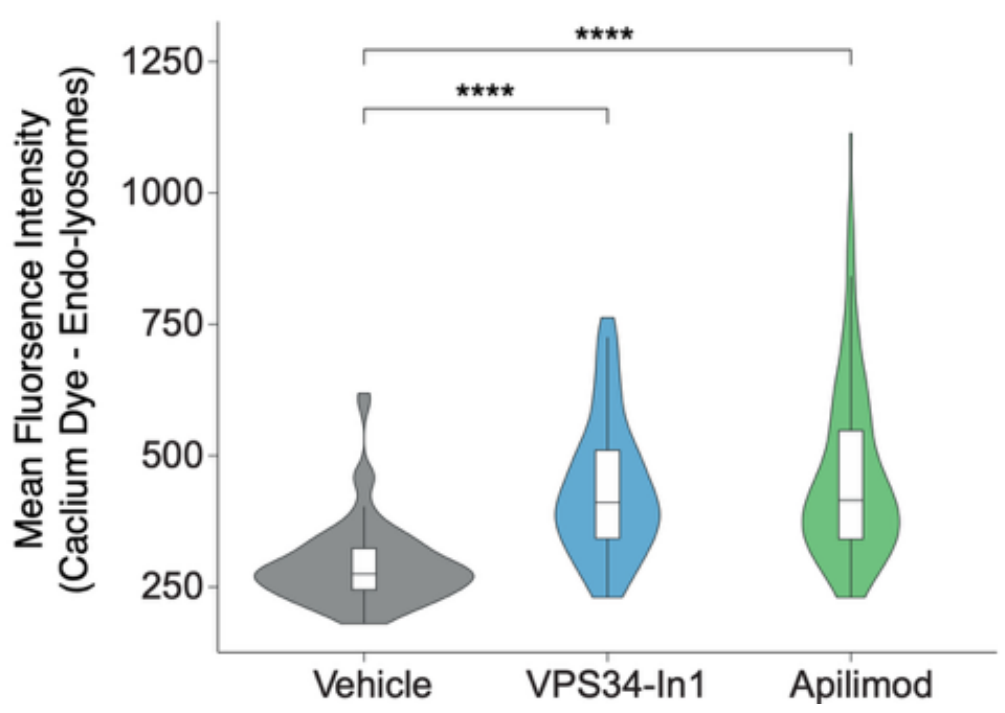

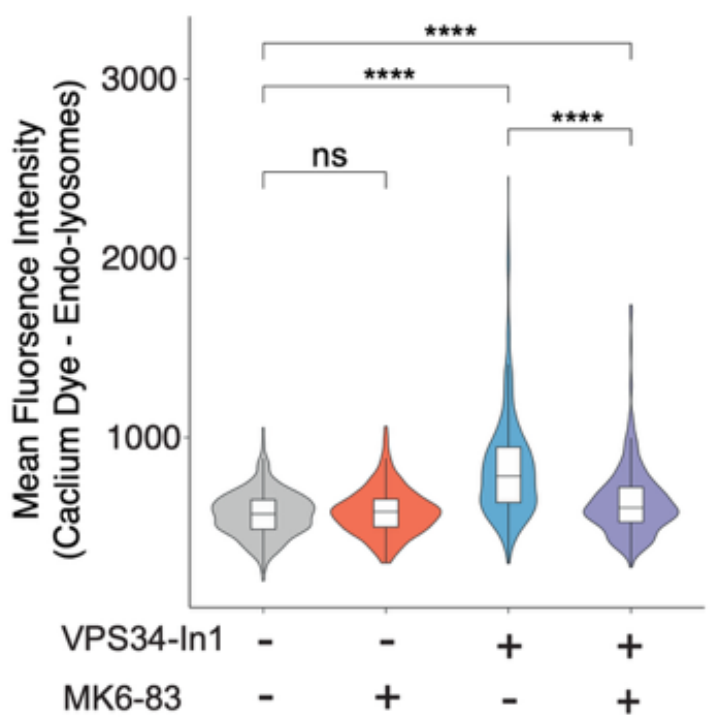

11) Fig.4B: a proper control is missing to validate that the decrease in fluorescence intensity measured over time is specifically caused by the addition of BAPTA-AM and not by photo-bleaching.

We agree and thank the Reviewer for alluding to this shortcoming. In response, applying the same approach used for the data shown in Figure 4B, we imaged vesicular Ca^{2+} following VPS34 and PIKfyve inhibition **in the absence or presence of BAPTA-AM,**

assessing an endpoint measurement. These experiments confirmed BAPTA-AM as cause of reduced signal intensity. These **additional data** have been included in the revised version of our manuscript as **Supplementary Figures S5B** and **S5C**:

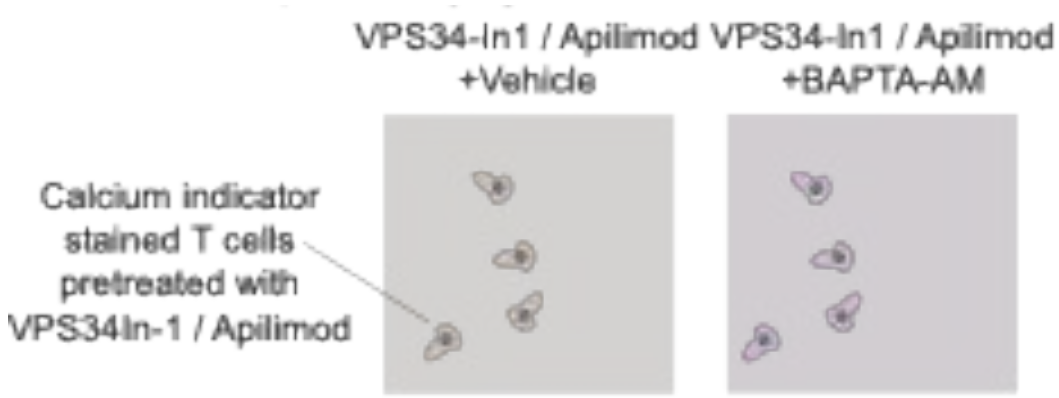

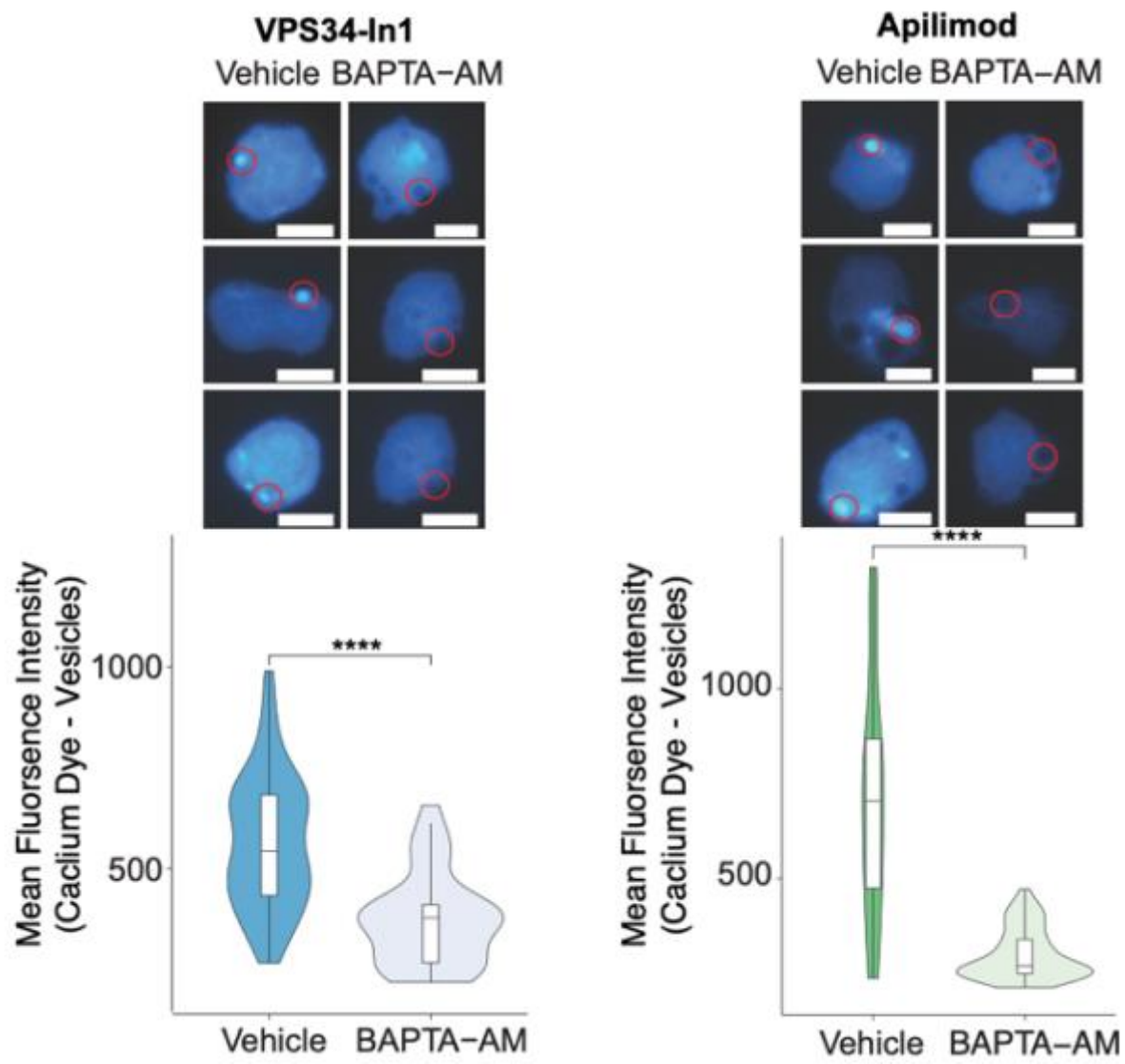

We thank the Reviewer for these important questions. To more thoroughly **probe how TRPML1 activation influences cell migration**, we monitored the **migration speed of murine naïve CD8$^+$ T cells exposed to a dose-range of the TRPML1 agonist, MK6-83.** We indeed observed that, **at 1 µM of MK6-83, T cell speed was increased** (5 µM MK6-83 was used in the rescue experiments presented in the original submission). Higher MK6-83 doses (10 µM), by contrast, significantly decreased the speed of migrating cells. This suggests a **narrow optimal range of lysosomal Ca^{2+} release that promotes migration.** These **new data have been included in the manuscript as Supplementary Figure S5F**:

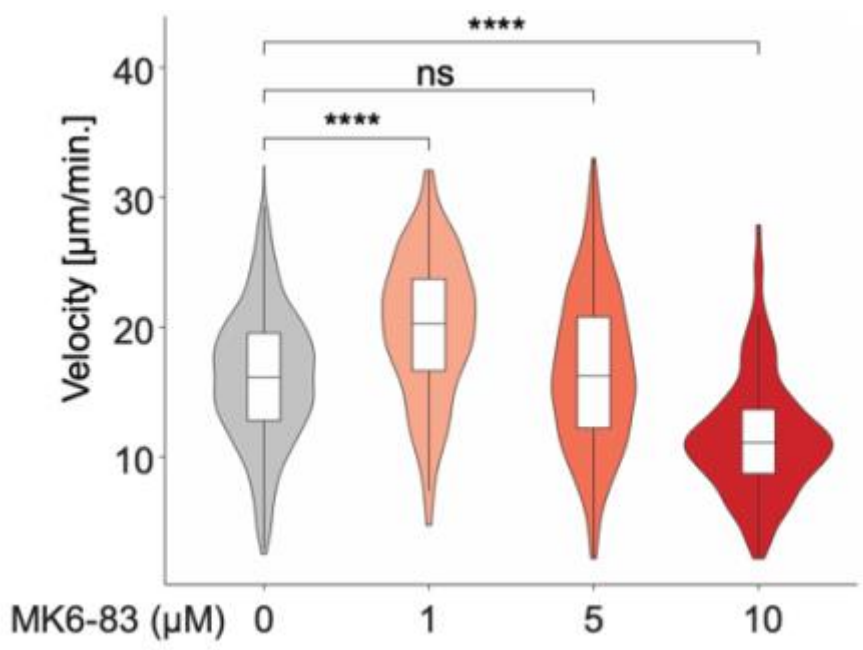

Regarding the absence of additional myosin RLC activation in cells treated with MK6-83, we can only speculate: Lysosomal Ca^{2+} represents only one of several components of the signaling network regulating RLC activation, which likely relies on multiple nodes and feedback loops, making the process non-linear. Alternatively, our method for phosphorylated RLC (pRLC) may lack sufficient sensitivity, temporal resolution, or both, since detecting pRLC is technically difficult.

To confirm that MK6-83 activates Ca^{2+} flux into the cytoplasm under conditions used in our work, we assessed Ca^{2+} levels in Calbryte-stained murine T cells by flow cytometry, following treatment with vehicle or 5 µM MK6-83 –the concentration used in all rescue experiments. MK6-83 treatment resulted in a **small but consistent increase in Ca^{2+} signal, as presented in Supplementary Figure S5E**:

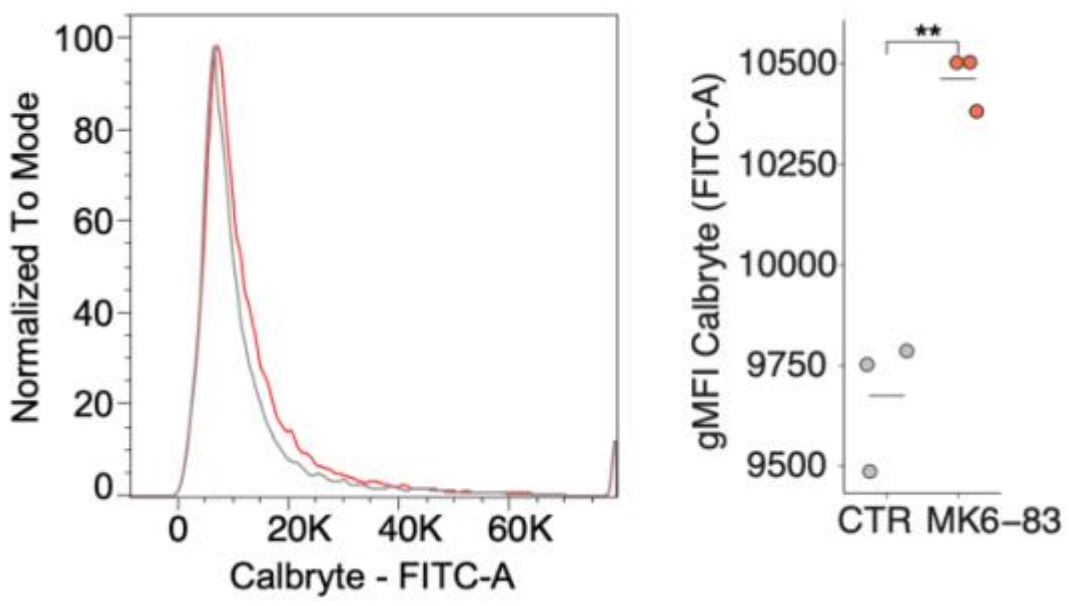

Minor points

- It is not clear how the migration experiments in Fig.1 have been performed as the under agarose assay is introduced later (next result section and Fig.2).

We apologize for this mistake that has been rectified. The migration experiments in Figure 1 were also performed using the under-agarose assay. This **assay is now introduced upon first use (i.e. with Supplementary Figure S1A)**.

- Fig.2A: It would help to include the lysotracker signal in the images.

Following this suggestion we have included images showing the lysotracker signal merged with brightfield images. This is presented in the revised **Figure 2C**:

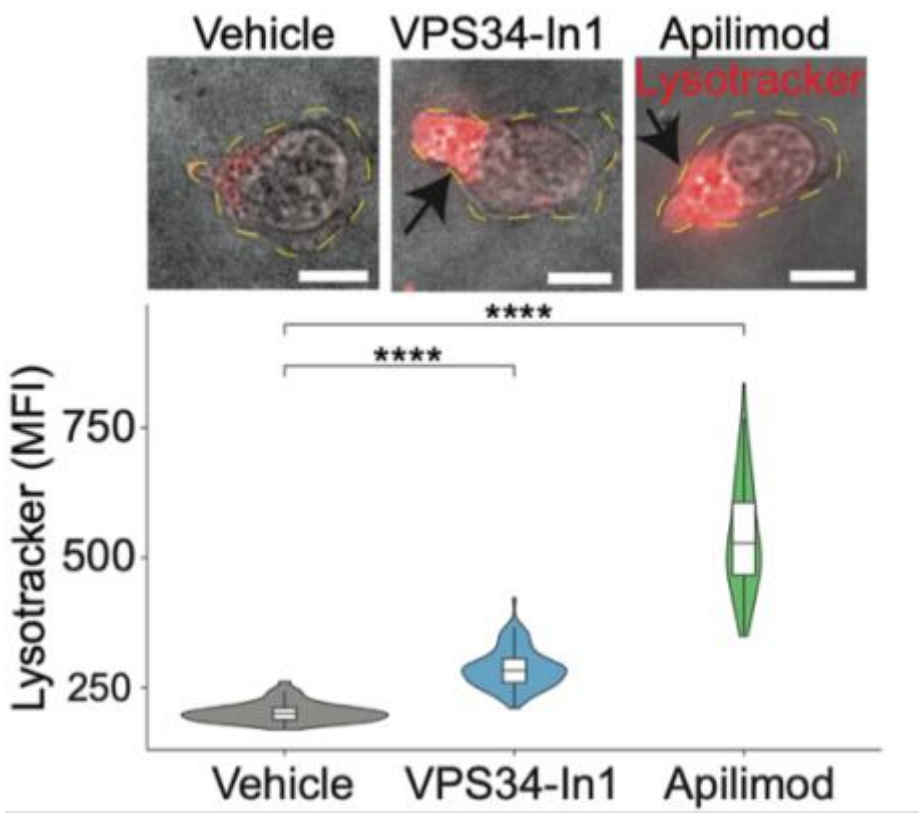

- Some spelling mistakes are present in the text (e.g. page 8:cirucluar, page 11: BATPA, etc.).

We thank the Reviewer for pointing out these errors that we now have corrected.

- Page 9: "inhibition of myosin IIA activity reduced migration speed of human CD8+ T cell blasts as well as human naïve CD8+ T cells, irrespective of upstream VPS34 activity (Fig. 3A-C)"- but it does not seem to be the case in Fig.3C where the mean speed further decreases with the addition of VPS34-ln1 in ML7 treated cells. No statistical analysis is provided between these two conditions (and data should not be pooled). Same in Fig. 3D between nocodazole treated sample and nocodazole+VPS34-ln1.

We apologize for this confusion. Please note that **no data were pooled when presenting these experiments**, **and the statistical analyses performed indicated no significant differences** in conditions with blocked *vs*. non-blocked upstream VPS34 activity. To clarify, in the revised manuscript, all statistical analyses have been added. Of note, inhibition of MLCK-dependent myosin IIa activation has previously been shown to be incomplete when using 10 µM ML-7 as used in our assays (please see data below from Solanes et al., 2015). This is likely explaining the stronger effect of VPS34 inhibition as compared to ML-7 that we observed in Figure 3C.

REDACTED: Fig. 6A, from Solanes *et al.* 2015

Reviewer #2: The manuscript by Dehio et al. describes a very interesting study made in T cells, which shows the involvement of the VPS34 and PIKfyve endolysosome-associated kinases in their migratory capacity. They found that these kinases control the speed of T cells but not their directionality. This is associated to the redistribution of lysosomes towards the T cell uropod in response of CCL19 internalization, which localizes to these vesicles. They further show that this pathway talks to the TRPML1-Myosin II axis previously identified in dendritic cells. This work comes reinforcing the concept of lysosomal signaling driving amoeboid migration, which is very important for the cell migration community to fully integrate it and move forward. Indeed, although this discovery had been recently made in dendritic cells and drosophila macrophages, the present work adds new important molecular players to the initially proposed mechanism and generalizes it to different types of amoeboid cells, thus enhancing its impact. This manuscript would therefore be a great candidate for publication in [REDACTED] once the comments and questions raised below will be addressed. It is generally very well-structured and written.

Thank you very much for this overall positive feedback.

Specific points:

1) Many results rely on inhibitors but lack genetic evidence. This is important as we all know that inhibitors are not always specific. Please provide shRNA-mediated depletions for PikFyve and TRPML1.

We agree with this critique. To address the concern, we knocked out PIKfyve in human $CD8^+$ T cell blasts using guide RNAs targeting the catalytic domain. The knockout was confirmed by sequencing the targeted genomic region, with indel percentages indicating a knockout efficiency close to 100%. Consistent with our inhibitor data, PIKfyve-KO T cells exhibited reduced migration speed but retained the ability to sense the chemokine gradient. Additionally, PIKfyve-KO cells displayed aberrant vesiculation at the uropod, a phenotype also seen when using a PIKfyve inhibitor. These **new data have been included in the manuscript as Figure 2G, and Supplementary Figures S2F,G**:

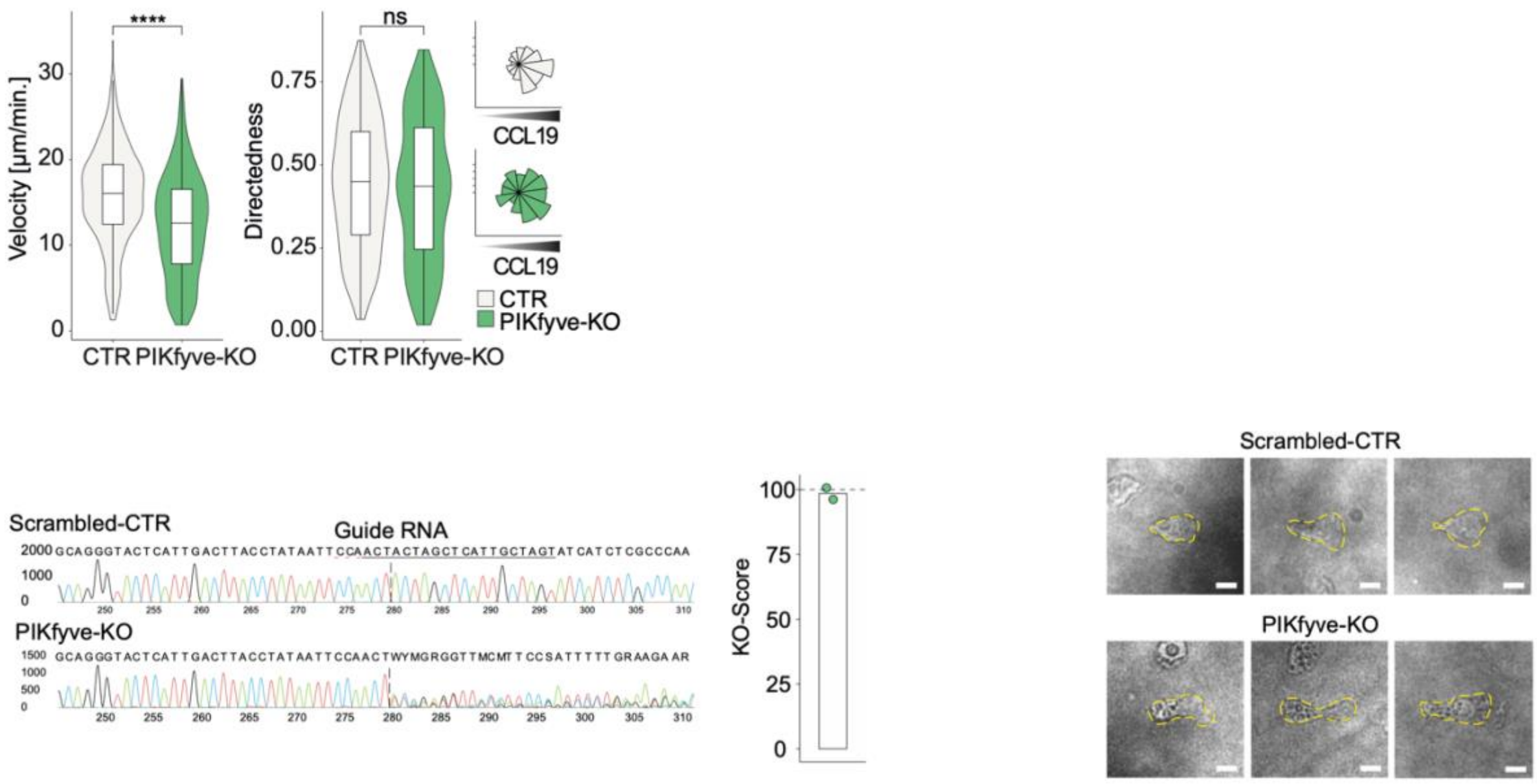

As further suggested by this Reviewer, we **also knocked down TRPML1 in human CD8$^+$ T cell blasts** via lentiviral transduction of shRNA, using a similar approach as applied for VPS34. The knockdown was verified by both Western blotting and mRNA quantification, and, **again, cells exhibited reduced migration speed. These important data have been incorporated in the revised manuscript as Figure 4G, and Supplementary Figure S5H**:

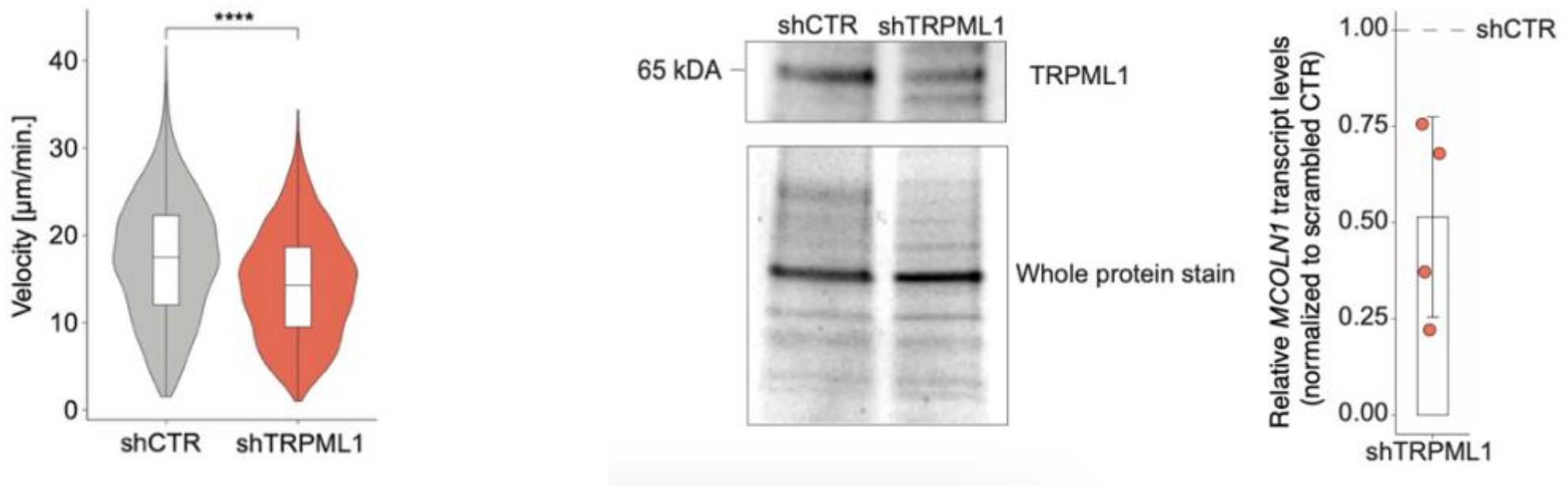

Our genetic interrogation is thus consistent with our inhibitor data, the genetic knockout of TRPML1 and PIKfyve orthologues in *Dictyostelium* cells, and previously published TRPML1 knockout studies in dendritic cells (Bretou et al., 2017).

2) TRPML1 is thought not to be highly expressed in T cells, as it is the case of mTORC1, which surprisingly was found not to have any effect when inhibited. Could the author show levels of expression in the cells they use in their experiments, for example by comparing them to the levels expressed by dendritic cells?

Following this advice, we first consulted the Human Protein Atlas (HPA). Indeed, TRPML1 is expressed at lower levels in T cells when compared to other immune cell types (please see HPA data below). However, our **Western blot analyses** from the newly added shRNA knockdown experiments (please see above) **readily detected TRPML1 protein in T cells**. Moreover, our functional data from knockdown and agonist studies confirm a functional role for TRPML1 in these cells.

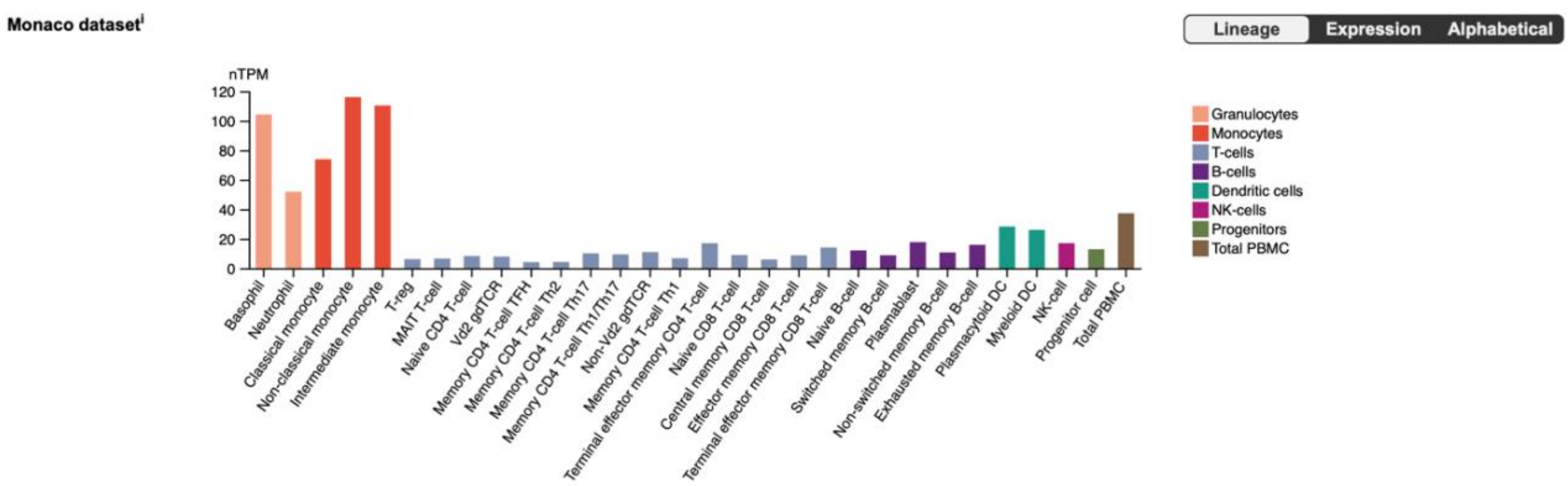

3) How heterogenous are the lysosomes that cluster at the T cell uropod? Also, Figure 1F: please provide quantification of total numbers of vesicles and statistics.

We thank the Reviewer for this relevant question. In response we have re-inspected our SEM data, which show clear heterogeneity of lysosomes at the T cell uropod. Specifically, our SEM data reveal endo-lysosomes of varying sizes and contents, which seems unsurprising, given that endo-lysosomes dynamically cycle through fusion of late endosomes with lysosomes followed by fission of lysosomes (below are examples of this heterogeneity – from Figure 1A – to illustrate this point):

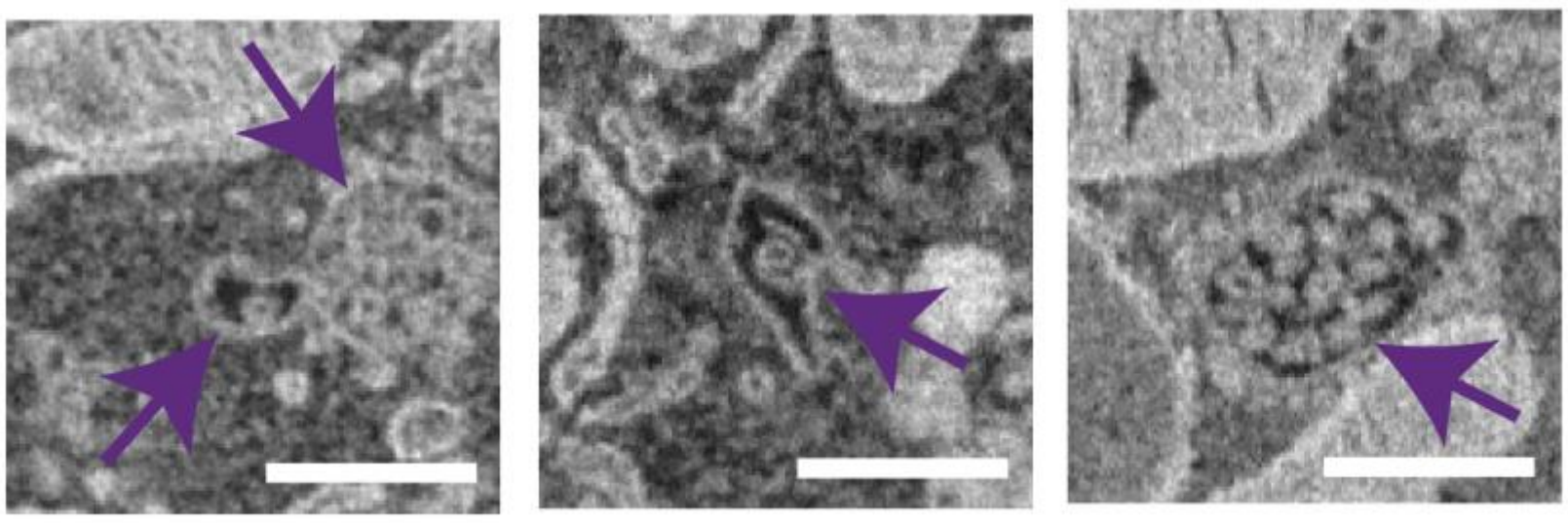

As outlined above (please see *Reviewer #1*, *Major point 3*), Figure 1F has been replaced by Figure 2B. In this figure we now have **quantified and statistically analyzed endo-lysosomes**. Please note that chemokine exposure does not appear to have a major impact on the total number of endo-lysosomes, as assessed by counting lysotracker-stained vesicles **(new Supplementary Figure S1B)**:

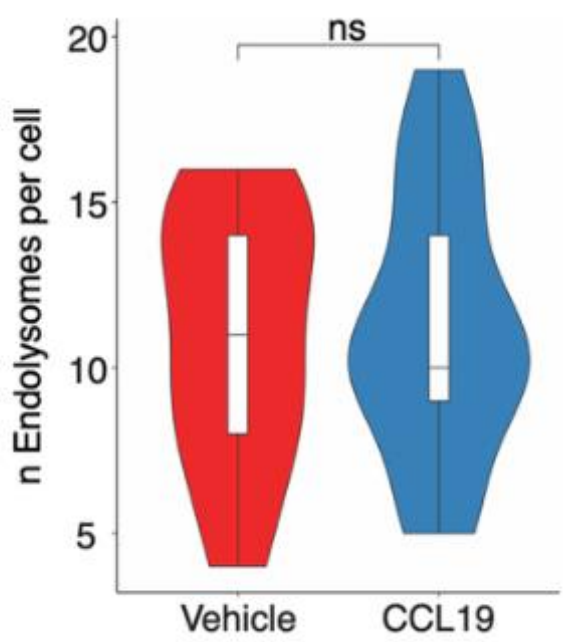

4) Does confinement under agarose gels trigger lysosome polarization to the uropod as observed in dendritic cells or is CCL19 strictly required? (**q1**) Is lysosomal signaling also involved in SDF1-induced T cell migration? (**q2**)

Thank you for these interesting questions.

***Ad* q1**: In our hands, ***naïve* CD8$^+$ T cells confined by agarose only polarize in the presence of CCL19**. This is shown in the **newly added data summarized in Figure 1B** (please see above, Reviewer #1, Major point 3). In this setting, clustering of lysosomes was only observed in CCL19 treated cells, with confinement evidenced by the x-maximum-intensity projection of cell nuclei.

***Ad* q2**: **CD8$^+$ T cell blasts polarize and migrate independent of chemokines** (Galeano Niño et al., 2020). In this context, **VPS34 activity was regulating migration speed both in presence and absence of CXCL12 (SDF1)**, as shown in **Supplementary Figures S3A,B**:

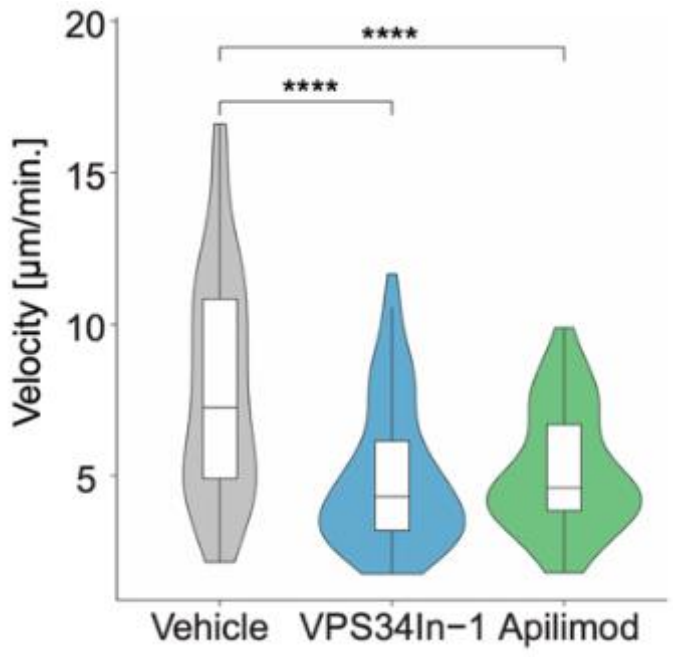

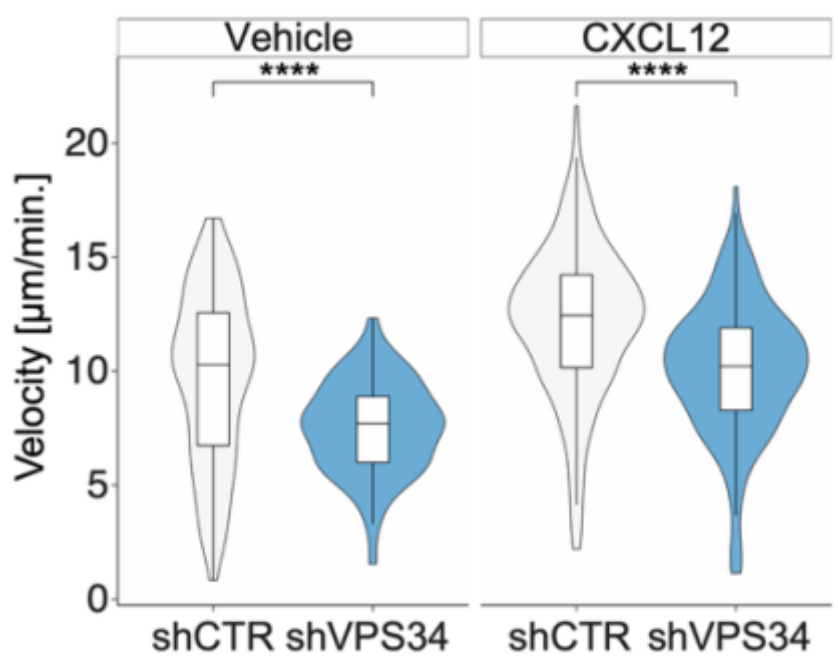

We are aware of published and, through personal communication, as yet unpublished work that suggests that polarization of confined T cells can occur independently of chemokines. Specifically this was shown for tissue-resident T cells (Ruef et al., 2023). Aligning with our data, in confined *naïve* T cells, no chemokine-independent polarization was observed also in the publication by Ruef and colleagues. This suggests that cell-intrinsic differences between naïve T cells, T cell blasts and tissue-resident T cells may shape cell-specific requirements for polarization.

5) The authors mention Rab7 in the discussion with respect to lysosome association to microtubules, however Rab7b has also been involved in tethering myosin IIA to lysosomes for activation by TRPML1. Could the authors assess whether Rab7 might also play this role in T cells?

While **Rab7b has been described as a tether of myosin II to lysosomes**, the same publication showed that **Rab7a does not bind myosin II** (Borg et al., 2014). The **myosin–Rab interaction thus seems complex in nature**. Therefore, while the Reviewer is raising an interesting point, **exploring this intricate additional biology was beyond the scope of the current study**. In response to this helpful comment we have, however, amended the discussion to include the notion of Rab7b as a potential co-modifier of the lysosomal VPS34–PIKfyve kinase system characterized in our manuscript.

6) Is the lysosomal signaling axis here identified also involved in the secretion of cytotoxic granules when CD8 T cells form a synapse with antigen presenting cells? How do lysosomes behave when T cells form synapses versus kinapses?

Thank you for this interesting thought. The topic raised is opening up broad and complex additional biologic questions. To begin exploring how degranulation (assessed by flow cytometry/Lamp1 cell surface expression) is affected by the VPS34–PIKfyve kinase system, we tested degranulation of murine CD8$^+$ T cell blasts upon stimulation with plate-bound anti-CD3 monoclonal antibodies. Inhibition of VPS34 had a strong inhibitory effect on degranulation, whereas inhibition of PIKfyve appeared to have a lesser impact (please see figure below – for Reviewer only):

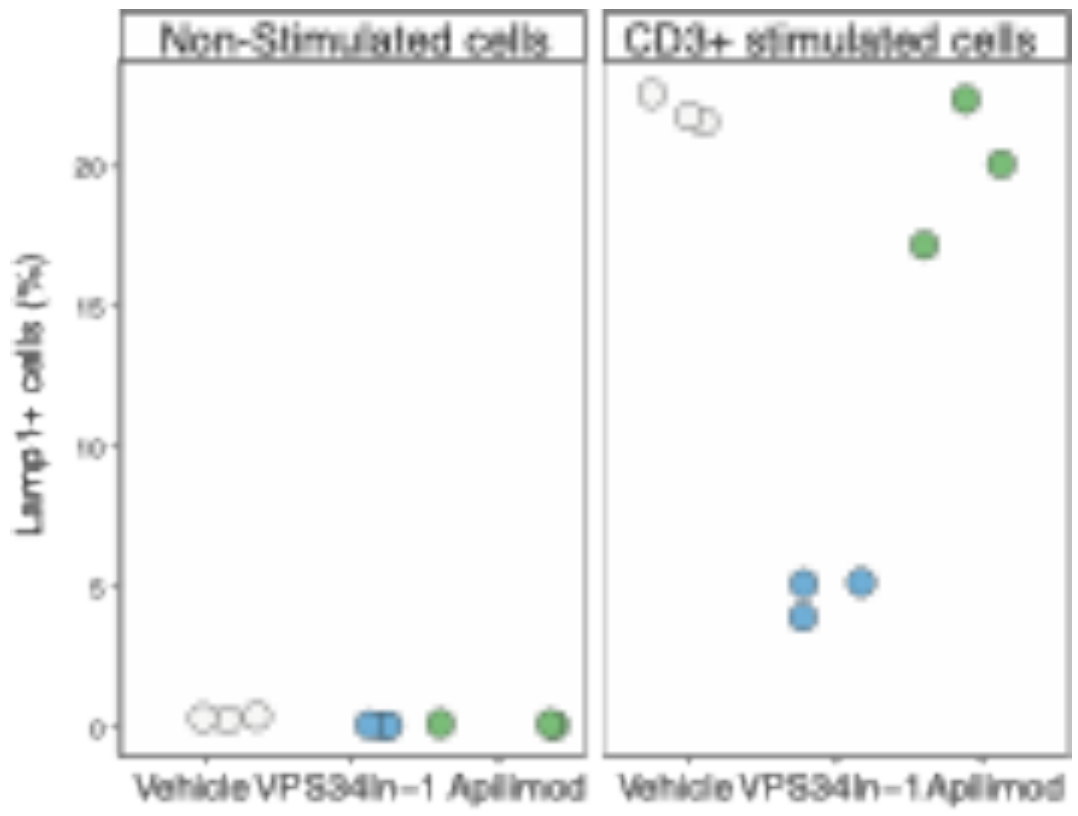

This suggests that PI(3)P-related signaling regulates degranulation, while PI(3,5)P_2 may be less involved. Notably, PIKfyve inhibition has been reported to promote exocytosis in neurosecretory cells (Osborne et al., 2008). Exocytosis of secretory granules thus likely represents one of the major differences between the immunological synapse and kinapses. Detailed investigation of how the VPS34–PIKfyve kinases contribute to the function of immune synapses was not the goal of this study. Yet, our preliminary work is placing us well to allude to this intriguing comparison (VPS34–PIKfyve kinase activity in kynapses *vs*. immune synapses) in our *Discussion* section. Please instruct accordingly.

Reviewer #3: This study demonstrates that the VPS34 and PIKfyve lipid kinases play a role in T cell migration. Activity of these kinases results in production of PI(3,5)P2, a lipid which the authors show is located on endo-lysosomes that are concentrated in the uropod of the migrating T cell. The authors show that chemokine induced migration of CD8 T cells is inhibited when cells are treated with inhibitors of VPS34 and PIKfyve or following RNAi-mediated knockdown of VPS34. Inhibition of VPS34 and PIKfyve causes reduced phosphorylation of the myosin regulatory light chain (RLC) and reduced actin retrograde flow, presumably because of reduced myosin IIA activity. Finally, the authors show that inhibition of VPS34 and PIKfyve results in increased Ca2+ in the lysosomes of migrating T cells and hypothesize that this is because PI(3,5)P2 is an activator of the TRPML1 Ca2+ channel. The authors support this idea by showing that treatment of cells with MK6-83, an activator of TRPML1 partially rescues the migration deficit induced by an inhibitor of VPS34. In summary, the study supports a model in which VPS34 and PIKfyve activity results in increased amounts of PI(3,5)P2 on endo-lysosomes which cause Ca2+ release viaTRPML1 and phosphorylation of myosin regulatory light chain and activation of myosin IIA, which is required for T cell migration. While other studies have pointed to the involvement of VPS34 and PIKfyve in cell migration, this work extends this to explain a role for PI(3,5)P2 in driving Ca2+ release via TRPML1 and subsequent myosin IIA activation.

We are pleased that this Reviewer concludes that our work adds significantly to the molecular understanding of cell migration.

Major points

1) The authors argue that inhibiting or knocking out VPS34 and PIKfyve blocks release of Ca2+ from lysosomes into the cytosol, and that since cytosolic Ca2+ is required for RLC phosphorylation, this explains why inhibition of the lipid kinases results in reduced RLC phosphorylation. If this is correct, the authors should demonstrate that cytosolic Ca2+ levels are reduced in cells treated with VPS34 and PIKfyve inhibitors, and in cells with a knockdown of these kinases.

Ca^{2+} signaling is spatiotemporally regulated by buffering organelles –such as the endoplasmic reticulum, mitochondria, and endo-lysosomes– which together are thought to form Ca^{2+} microdomains (as referenced in our *Discussion* – Atakpa et al., 2018; Burgoyne et al., 2015; Peng et al., 2020). **How Ca^{2+} flux into the cytoplasm through activation of TRPML1 by $PI(3,5)P_2$ integrates into this complex system remains unknown**. However, **lower *global* cytoplasmic Ca^{2+} levels** upon VPS34 or PIKfyve inhibition, as suggested by the Reviewer, **may not necessarily be an outcome**.

In addition to these biologic considerations, imaging subcellular Ca^{2+} is also challenging from a technical perspective (wide dynamic ranges and rapid spatio-temporal fluctuations). In our experimental system specifically, using a Ca^{2+} indicator we observed rapid accumulation of the dye in vesicles –likely lysosomes, as previously described (Trollinger et al., 2000). Unfortunately, the high vesicular signal intensity in VPS34- or PIKfyve-inhibited cells prevented us from reliable measuring non-vesicular/cytosolic Ca^{2+} levels. Several additional attempts were made to image cytosolic/microdomain selective Ca^{2+} abundance. Unfortunately, neither knockdown of VPS34 combined with Ca^{2+} sensor expression, nor T cells from GCaMP5s (Akerboom et al., 2012) mice (expressing a Ca^{2+} indictor) yielded reliable results.

Recognizing this limitation, we **propose to include a *'Limitations of the Study'* section** in our manuscript. Please instruct us accordingly.

2) Is the activity of VPS34 and PIKfyve altered by chemokine signaling? Do the levels of PI(3,5)P2 change in response to chemokine.

This is an interesting yet complex question. Our newly generated data (described in response to *Reviewer #2, Major point 4*) establish that **chemokine signaling is dispensable for regulation of T cell migration speed by VPS34 and PIKfyve**. Furthermore, the **data from Dictyostelium cells summarized in Fig. 5C-D were obtained in the absence of chemokine activation**. However, **whether VPS34 and/or PIKfyve activities are altered by chemokine signaling remains unknown**. It is plausible that chemokine receptor endocytosis and recycling increase the activity of this pathway, as the VPS34–PIKfyve axis is known to be involved in receptor recycling and ligand degradation. Direct quantification of PI(3)P and PI(3,5)P_2 –e.g., by mass spectrometry– is extremely challenging due to low abundance. Recently, a PI(3,5)P_2 reporter has become available (Vines et al., 2023). However, similar to the 2xFYVE-eGFP probe, it is useful for assessing the presence and localization of PI(3,5)P_2, but does not provide quantitative measurements. Given these considerations, we propose to highlight this interesting question as an outlook in our *Discussion*. Please instruct us accordingly.

3) Fig 4F. Why does treatment of cells with MK6-83, the TRPML1 activator, cause a decrease in phosphorylation of RLC? The authors' model predicts that phosphorylation of RLC should increase.

We apologize for insufficient clarity here. RLC phosphorylation upon MK6-83 treatment **was not statistically different** in both immunofluorescence and Western blot analyses (statistical analyses now added). Please see above reg. our discussion of the fact that RLC phosphorylation was not detected upon treatment with a TRPML1 agonist (*Reviewer #1, Major point 12*).

In many parts of the manuscript the authors provide example data, e.g. in the form of images, and use these to make conclusions. Any such conclusions must be supported by data from multiple biological repeats, across several independent experiments and requires statistical analysis to support any conclusions that are made in the text. See following points.

4) Fig. 1A. Authors state that endo-lysosomes polarize to one side of a migrating T cell but give just images from single cells to support this statement.

5) Fig 1B. Authors state that endo-lysosomes are polarized to one side of the T cell, but again provide only example images.

6) Fig. 1C. Authors state that vesicles with CCL19 polarize to the uropod. Again, only example images are provided. The same issue of example images with no repeats and

statistical validation being used to make conclusions is true for Fig. S1A, B and C, Fig S2A, Fig 2B (right panel).

This is a very valid and important critique, thank you. We now have **added more representative images, performed additional experiments (biological replicates), and added quantification with statistical testing where applicable**, as detailed above (*Reviewer #1, Major point 3*).

Briefly, we now analyzed seven cells per condition for **Figure 1A** and included statistical analysis of endo-lysosomal positioning, confirming similar organellar distributions as observed in our previous SEM experiment with murine naïve $CD8^+$ T cells (vehicle and CCL19-treated). Additionally, we quantified (using our new purpose-built, automated analysis pipeline) endo-lysosomal polarization in immunofluorescence experiments and added more representative images in **Figure 1B**. Using the same pipeline, we also analyzed endo-lysosomal movement in live cells stained with lysotracker, and quantified the lysosomal polarization and numbers in 3D by spinning disk imaging. This new data is found in **Figure 1C** and supplementary Figure 1B and replaced former Figure S1A. Further, we displayed and analyzed more cells in **Figure 1D** and added a repetition experiment. In **Figure S1C** (before Figure S1B), we now also display more representative images.

Regarding quantification of the data shown in **Figure 2B, right panel (now Figure S3C)**, we opted to measure chemokine uptake by flow cytometry. We also repeated the experiments summarized in **Figure S2A**, which confirmed our initial findings, and aligns with published data (Bago et al., 2014; Munson et al., 2015).

We are grateful to the Reviewers for tasking us with extensively revising the manuscript regarding quantification, replication and statistical interrogation, as we feel that this additional effort has significantly strengthened the study.

7) Fig 1F. We are shown an example image of 2xFYVE-eGFP labelled structures in migrating T cells. How do we know these are in the uropod? in endo-lysosomes? We are shown polarized and non-polarized cells? How were these two types of cells objectively defined? Was a measurement of polarization used? If so, what was it? What is the meaning of the column graph? Does it show more 2xFYVE-eGFP labelled structures in polarized T cells? If so, this needs statistical confirmation. Why would the number differ between polarized and non-polarized cells?

Thank you for challenging us regarding these data, and apologies for insufficient clarity. In response we have **imaged 2xFYVE-eGFP and lysotracker co-stained T cells using spinning-disk 3D microscopy (new Figure 2B)**. This revealed significant overlap between 2xFYVE-eGFP structures and endo-lysosomes. These experiments were performed in biological triplicates, establishing reproducibility (please see *Reviewer #1, Major point 3*). We refrained from attempts to quantify vesicle numbers between vehicle- and CCL19-treated cells in these experiments.

Dear Christoph,

Thank you for the submission of your manuscript and please apologize the delay in handling it. To recapitulate, your manuscript had been reviewed at another journal and you have subsequently revised it and submitted it to our journal. We have taken the anonymous referee reports into account and asked two experts in the field to act as arbitrators and to assess whether you have adequately addressed the concerns raised.

As you know, we have received the reports and further feedback from both arbitrators copied below. Arbitrator/Referee #2 is a generalist on adhesion, cytoskeleton and cell migration. Arbitrator/Referee #1 is an expert in cell migration in the context of the immune system.

Referee #1 considers your study of interest but raises concerns that you might be studying the consequences of disturbance in PI3P homeostasis rather than a specific effect of endosomal positioning. This concern challenges the conclusion that the effects on migration velocity are due to localized endo-lysosomal lipid signaling. The expert advisor suggests performing experiments knocking down ARL8b or FYCO1 to test whether these perturbations produce a similar phenotype but was also open to the option to carefully phrase and tone down the conclusions made.

You have meanwhile provided a response to the remaining concerns and I agree to the proposal to discuss the limitations and to carefully phrase conclusions made.

Before we can proceed with the official acceptance of your manuscript, I need you to format it along our guidelines. I copy the general formatting guidelines below, but in order to streamline the process, I list a few points that you will need to change below.

Please,

- submit the synopsis image (Graphical abstract) and the summary text as separate files.
- write the abstract in present tense
- use the alphabetical Harvard style for the references, and et al if there are more than 10 authors.
- submit the figures as individual high-quality files
- define the exact p-values either in the legends or the figure panel (unless the p-value is <0.0001).
- make sure to define "n" in the figure legends and whether these are independent or technical replicates.
- remove the author contributions from the manuscript text and make sure everything is up-to date in the online submission system. (We use CRediT to specify the contributions of each author in the journal submission system. You can use the free text box in our system if you wish to provide more detailed descriptions.)
- Declaration of interest is called "Disclosure and competing interests statement"
- order the manuscript sections as follows: Title page - Abstract - Introduction - Results - Discussion - Methods - Acknowledgements - Disclosure and competing interests statement - References - Figure legends - Tables and their legends (not EV tables) - Expanded View Figure legends
- Supplemental information is either Expanded View or Appendix. You have 6 Supplemental figures. These could all be Expanded View figures. The nomenclature is "Figure EV#" and the legends of these are part of the manuscrip text after the main figure legends.
- fill a Reagents and Tools table and upload it as separate file (attached)
- fill the Author Checklist (attached)
- prepare Source Data (see point 8 below). You can either submit the raw data to us (one folder per figure with subfolders for each panel) or upload it to e.g., BioStudies. In the latter case you need to provide the link to the dataset in the Data Availability section.
- provide an ORCID for the corresponding author, Christoph Hess. See point (6) below.
- make sure that the information on funding in the Acknowledgments is congruent with the information provided in the submission system.
- state the use of BioRender in the methods section as follows:
Graphics:
(some of the... OR Figure #... OR synopsis) Graphics were created with BioRender.com.

I think these are the most important points. Once you submit the revised manuscripts, our assistants will check in essence for the points listed above. In addition, we will perform a data integrity check on all figures provided, as our standard procedure. If formatted according to these guidelines, the checks should be quick and we can proceed with publication of your study.

I list below again the general formatting guidelines.

GENERAL FORMATTING GUIDELINES:

When submitting your revised manuscript, please review the submission guidelines (https://link.springer.com/journal/44319/submission-guidelines#cms-Revised-submissions) and carefully review the instructions that follow below. Failure to include requested items will delay the evaluation of your revision.

1) a .docx formatted version of the manuscript text (including legends for main figures, EV figures and tables). Please make sure that the changes are highlighted to be clearly visible.

2) individual production quality figure files as .eps, .tif, .jpg (one file per figure). See hhttps://media.springernature.com/original/springer-cms/rest/v1/content/27825798/data/v1 for more info on how to prepare your figures.

3) We replaced Supplementary Information with Expanded View (EV) Figures and Tables that are collapsible/expandable online. A maximum of 5 EV Figures can be typeset. EV Figures should be cited as 'Figure EV1, Figure EV2" etc... in the text and their respective legends should be included in the main text after the legends of regular figures.

- For the figures that you do NOT wish to display as Expanded View figures, they should be bundled together with their legends in a single PDF file called *Appendix*, which should start with a short Table of Content. Appendix figures should be referred to in the main text as: "Appendix Figure S1, Appendix Figure S2" etc. See 'Expanded View' section of the submission guidelines.

- Additional Tables/Datasets should be labeled and referred to as Table EV1, Dataset EV1, etc. Legends have to be provided in a separate tab in case of .xls files. Alternatively, the legend can be supplied as a separate text file (README) and zipped together with the Table/Dataset file.

4) a .docx formatted letter INCLUDING the reviewers' reports and your detailed point-by-point responses to their comments. As part of the EMBO Press transparent editorial process, the point-by-point response is part of the Review Process File (RPF), which will be published alongside your paper.

5) a complete author checklist, which you can download from our author guidelines. Please insert information in the checklist that is also reflected in the manuscript. The completed author checklist will also be part of the RPF.

6) Please note that all corresponding authors are required to supply an ORCID ID for their name upon submission of a revised manuscript (<https://orcid.org/>), which can be linked to your profile in the manuscript submission system.

7) Before submitting your revision, primary datasets produced in this study need to be deposited in an appropriate public database. Please remember to provide a reviewer password if the datasets are not yet public. The accession numbers and database should be listed in a formal "Data Availability" section placed after Methods. Please note that the Data Availability Section is restricted to new primary data that are part of this study. * Note - All links should resolve to a page where the data can be accessed. *

If your study has not produced novel datasets, please mention this fact in the Data Availability Section.

8) At EMBO Press we ask authors to provide source data for the main manuscript figures. You will receive a separate email with instructions for providing source data with your revised manuscript, including how to upload and organize the files.

9) Our journal also encourages inclusion of *data citations in the reference list* to directly cite datasets that were re-used and obtained from public databases. Data citations in the article text are distinct from normal bibliographical citations and should directly link to the database records from which the data can be accessed. In the main text, data citations are formatted as follows: "Data ref: Smith et al, 2001" or "Data ref: NCBI Sequence Read Archive PRJNA342805, 2017". In the Reference list, data citations must be labeled with "[DATASET]". A data reference must provide the database name, accession number/identifiers and a resolvable link to the landing page from which the data can be accessed at the end of the reference.

10) Regarding data quantification, the following points must be specified in each figure legend:

- the name of the statistical test used to generate error bars and P values,

- the number (n) of independent experiments (please specify technical or biological replicates) underlying each data point,

- the nature of the bars and error bars (s.d., s.e.m.),

- If the data are obtained from n {less than or equal to} 5, show the individual data points in addition to the SD or SEM.
- If the data are obtained from n {less than or equal to} 2, use scatter blots showing the individual data points.

Discussion of statistical methodology can be reported in the materials and methods section, but figure legends should contain a

basic description of n, P and the test applied.

- Please also include scale bars in all microscopy images.

11) The journal requires a statement specifying whether or not authors have competing interests (defined as all potential or actual interests that could be perceived to influence the presentation or interpretation of an article). In case of competing interests, this must be specified in your disclosure statement.

12) All Materials and Methods need to be described in the main text using our 'Structured Methods' format, which is required for all research articles. According to this format, the Methods section includes a Reagents and Tools Table (listing key reagents, experimental models, software and relevant equipment and including their sources and relevant identifiers) followed by a Methods and Protocols section describing the methods using a step-by-step protocol format. The aim is to facilitate adoption of the methodologies across labs. More information on how to adhere to this format as well as a downloadable template (.docx) for the Reagents and Tools Table can be found in our author guidelines.

We would also welcome the submission of cover suggestions, or motifs to be used by our Graphics Illustrator in designing a cover.

As part of the EMBO publication's Transparent Editorial Process, EMBO reports publishes online a Review Process File (RPF) to accompany accepted manuscripts. This File will be published in conjunction with your paper and will include the referee reports, your point-by-point response and all pertinent correspondence relating to the manuscript.

You are able to opt out of this by letting the editorial office know (emboreports@embo.org). If you do opt out, the Review Process File link will point to the following statement: "No Review Process File is available with this article, as the authors have chosen not to make the review process public in this case."

I look forward to seeing a revised form of your manuscript when it is ready.

Kind regards,

Martina

Martina Rembold, PhD
Senior Editor
EMBO reports

==================

Arbitrator/Referee #1:

This revised manuscript investigates the contribution of endo-lysosomal lipid kinase signaling (VPS34-PIKfyve axis) to the control of amoeboid cell migration in T cells and other amoeboid systems. The authors propose that a sequential cascade of VPS34- and PIKfyve-mediated $PI(3,5)P_2$ production regulates Ca^{2+} efflux from lysosomes through TRPML1, modulating myosin IIA activity and thereby controlling migration velocity. Overall, the study is ambitious and well presented, and the revisions since the previous review cycle are substantial and have greatly improved the manuscript.

The paper is conceptually engaging and offers a potentially important framework linking endo-lysosomal dynamics and cytoskeletal control of T cell migration. The authors' efforts to address previous reviewer concerns are evident. Substantial new imaging, pharmacological, and mechanistic data have strengthened their conclusions. The high volume of revisions and additional experiments is commendable and demonstrates responsiveness to feedback.

However, while the data are compelling, my enthusiasm remains tempered by the global effects of the pathways studied, which complicate the mechanistic interpretation. The inhibitors used target central nodes of membrane trafficking and autophagy, and therefore their observed impact on migration may reflect widespread cellular dysfunction rather than a discrete, migration-specific mechanism dependent on local Calcium release, which nevertheless contributes to migration.

My main conceptual reservation lies in the pleiotropic impact of these kinases. Inhibition of VPS34 or PIKfyve inevitably affects endocytic trafficking, autophagy flux, and lysosomal homeostasis. Hence, disruptions in migration velocity could be secondary to broader alterations in cell physiology rather than directly attributable to localized endo-lysosomal lipid signaling. This confounding factor should be more explicitly addressed in the discussion. For instance, VPS34 inhibition has been reported to influence global protein synthesis rates, which directly contribute to cytoskeletal dynamics and energy metabolism. The authors

might consider discussing and document whether reductions in biosynthetic capacity could also contribute to the observed reduction in motility.

The data do not clearly establish whether the spatial localization of lysosomes is a cause or a consequence of the migratory phenotype. While polarized lysosome distribution is observed, it remains uncertain whether this polarization actively promotes migration, or merely reflects the migration state. Further clarification or experimental approaches targeting endosomal positioning regulators could be informative. For instance, the impact of nocodazole on endosome positioning is likely and how it counteracts VPS34 inhibition is not well dissected in the paper. Given that microtubule depolymerization itself alters endosomal trafficking and cell polarity, it is difficult to interpret this interaction mechanistically without additional markers or quantitative positioning analyses, that should yield substantial insights on the importance of positioning. Where is the MTOC in the polarized T cells responding to chemokines and how MT depolymerization impacts positing and affects migration? This also alludes to the distinction between secretory granules and endo/lysosomes in the migrating cells, which was mentioned in the previous round of review and the release of which is affected by calcium signaling.

In figure S6D, the authors present a graph showing the number of proteins with a FYVE domain across species. This has no real interest and is purely speculative, unless it is used to disentangle position-dependent from metabolic effects of inhibiting VPS34, by silencing or editing regulators of endosomal positioning having direct or indirect interactions with PI3P, such as ARL8b, RILP, FYCO1, RUFY3, RAB7 or PLEKHM1. ARL8b, FYCO1 or RUFY3 are known to affect endosomal positioning and cell migration. Testing whether these manipulations phenocopy or diverge from VPS34-PIKfyve inhibition in terms of migration velocity would significantly strengthen the mechanistic argument and limit the interpretive breadth of the current dataset.

Conclusion

This work is scientifically interesting and technically sound, with major progress since the previous review. Although, I dislike asking for further experimental addition at this stage, the study's mechanistic clarity is hindered by the broad cellular roles of VPS34 and PIKfyve, which challenge the specificity of the conclusions regarding migration regulation. Clarifying whether lysosomal positioning per se drives the observed effects and distinguishing this from general defects in trafficking and metabolism, would substantially enhance the manuscript's impact.

Arbitrator/Referee #2:

Thanks for an opportunity to look at this manuscript. Please note that I've read it as a generalist rather than a technical expert. As well, I've not seen the earlier version of the manuscript.

Overall, I think this presents a good-faith effort to respond to the reviewers. Of note,
a) The authors have included quite extensive new experimental manipulations, including shRNA depletion and CRISPR/Cas9 KO in T-cells that complement their drug inhibitor studies.
b) New experiments (including FIB-SEM studies) and quantitative (re)analysis of their data.
Together, these seem very reasonable for a revision of a manuscript. Some of the other things requested by reviewers seem more suitable for a later study.

In terms of knowledge gain, this study represents a valuable advance in understanding how endolysosomal regulation contributes to cell migration. This is a long-standing question that was overshadowed by advances in elucidating how the cytoskeleton contributes to migration. Thus, it is valuable to develop this area further - especially as the current study identifies impacts on the cytoskeleton (myosin II activation and retrograde cortical flow) that help integrate these two drivers of migration.

I note that the authors have removed some material from the earlier version. Based on their response to reviewers, these seem justified. Certainly, in its current form the manuscript tells a tight, cohesive story.

In summary, this seems appropriate for EMBO Reports, both in knowledge gain and experimental rigour. Whether there are technical issues in the new experiments that might require another look by the original reviewers is a matter that I will have to leave in your hands.

====================

Feedback Arbitrator/Referee #2

I'm happy to defer to my expert colleague, as I am reading this as a generalist. Certainly, it is difficult to exclude secondary effects without approaches such as optogenetics or knocking-sideways sequestration. But this seems to me to be a separate study. And how much would more detailed studies develop the current story?

Feedback Arbitrator/Referee #1

I mostly agree with my colleague, however if one reads the manuscript it starts with endosomal positioning and ends up with Ca+ signaling and the real impact of endosomal positioning is not really dealt with? Rather the consequences of disturbance in PI3P homeostasis are studied , which by the way are completely different since one is partially blocking synthesis (VPS34i) and the other increasing PI3P levels (PIKFYVE inhibition), and nevertheless give the same results in altering migration? Thus, If we do not ask the authors a set of additional experiments testing how endo/lysosome positioning is truly impacting migration by altering specifically some key proteins with FYVE domains, the manuscript has to be rewritten for avoiding to be too definitive on the role of endo/lysosome positioning in controlling cell migration within the frame of the presented experiments.

The majority of editorial requests have been addressed by the authors.

Manuscript number: EMBOR-2025-63320V2
Title: A conserved VPS34-PIKfyve-TRPML1-Myosin II axis regulates the speed of amoeboid cell migration
Author(s): Philippe Dehio, Céline Michard, Juan Carlos Yam-Puc, Adrià-Arnau Martí i Líndez, Anett Jandke, Gunhild Unterstab, Lucien Fabre, Loic Sauteur, Marc Artinger, Daniel Legler, Michael Sixt, Thorsten Schaefer, Matthias Wymann, Klaus Okkenhaug, Thierry Soldati, Matthias Mehling, and Christoph Hess

Dear Christoph,

Thank you for your patience while we have reviewed your revised manuscript from the editorial side. Everything looks fine, except for a few points.

I am therefore writing with an 'accept in principle' decision, which means that I will be happy to accept your manuscript for publication once a few minor issues/corrections have been addressed, as follows.

- Please provide a point-by-point response to the concerns raised by referee/arbitrator #1. It will be published in our review process file and provide transparency on the changes you implemented. Which reminds me of the last comment from this expert, regarding the data shown in Figure EV6D. The referee considered these data very speculative and I wondered whether these are necessary or if at least, this caveat could be explicitly mentioned in the manuscript text.

- Please describe all your findings in the abstract in present tense. E.g., "Activity of VPS34 and PIKfyve regulates speed,..."

- Please reformat the references: et al needs to be used after 10 author names; DOIs should only be used for preprints and datasets that have not been published yet.

- The information on funding provided in the manuscript and in the online submission system must be congruent. Here we noted that the following funders are acknowledged in the manuscript but are missing in the system: Scientific Service Units (SSU) of ISTA through resources provided by the Imaging & Optics Facility (IOF) and the Lab Support Facility (LSF), AlumniMedizin Basel, and the Freiwillige Akademische Gesellschaft Basel.

- Materials and methods should be Methods.

- The manuscript should be in the following order: Title page - Abstract - Introduction - Results - Discussion - Methods - Data Availability - Acknowledgments - Disclosure Statement & Competing Interests - References - Figure Legends - (Main Tables with legends if applicable) - Expanded View Figure Legends.

- Our data editors checked the figure legends for accuracy and completeness. Please address the following points in the legends:
1) Please indicate the statistical test used for data analysis in the legend of figure 1A
2) Please note that the box plots need to be defined in terms of minima, maxima, centre, bounds of box and whiskers, and percentile in the legends of figures 1A, B, C; 2C-I, 3B, C, D, F, H, I; 4A, D, E, F, G; 5A-D; EV1 B, EV2 E, H, J; EV3 A, B; EV4 A, EV5 A, C, D, F.
3) Please note that information related to n is missing in the legends of figures 2J, K
4) Please note that the error bars are not defined in the legends of figures 2J, K; EV4 B, EV5G, H.
5) Please note that the yellow dashed borders are not defined in the legend of figure EV2 G, EV3 C. This needs to be rectified.
6) Please note that the red circles are not defined in the legend of figure EV5 C. This needs to be rectified.

- We perform a spot check of the supplied source data. I am not sure whether the image "Vehicle_2_YMIP" is the correct one? The "ZMIP" matches but this view seems different.
- Moreover, could you please upload the source data as individual folders per figure? Currently, all 5 folders are zipped into one file. Thank you.

- I kindly ask you to provide a short point-by-point response to the editorial points so that we can check the changes quickly. These should be the final formatting changes.

Once you have made these minor revisions, please use the following link to submit your corrected manuscript:

Link Not Available

If all remaining corrections have been attended to, you will then receive an official decision letter from the journal accepting your manuscript for publication in the next available issue of EMBO reports. This letter will also include details of the further steps you need to take for the prompt inclusion of your manuscript in our next available issue.

Thank you for your contribution to EMBO reports.

Kind regards,

Martina

Martina Rembold, PhD
Senior Editor
EMBO reports

Point-by-point response to editorial changes

- Please provide a point-by-point response to the concerns raised by referee/arbitrator #1. It will be published in our review process file and provide transparency on the changes you implemented. Which reminds me of the last comment from this expert, regarding the data shown in Figure EV6D. The referee considered these data very speculative and I wondered whether these are necessary or if at least, this caveat could be explicitly mentioned in the manuscript text.

We now provide a point-by-point response to the referees comments. In this process, we also removed EV6D and amended the text of the manuscript accordingly.

- Please describe all your findings in the abstract in present tense. E.g., "Activity of VPS34 and PIKfyve regulates speed,…"

The abstract is now written is present tense and updated on the submission platform as well.

- Please reformat the references: et al needs to be used after 10 author names; DOIs should only be used for preprints and datasets that have not been published yet.

The references are updated accordingly.

- The information on funding provided in the manuscript and in the online submission system must be congruent. Here we noted that the following funders are acknowledged in the manuscript but are missing in the system: Scientific Service Units (SSU) of ISTA through resources provided by the Imaging & Optics Facility (IOF) and the Lab Support Facility (LSF), AlumniMedizin Basel, and the Freiwillige Akademische Gesellschaft Basel.

The funders information is now concurrent between the text and the submission platform. Please note that the SSU from ISTA are the facilities and not funders. We added: "This research was technically supported by..." to clarify this. Also, a new funder for Christoph Hess was added.

- Materials and methods should be Methods.

We formatted the manuscript accordingly.

- The manuscript should be in the following order: Title page - Abstract - Introduction - Results - Discussion - Methods - Data Availability - Acknowledgments - Disclosure Statement & Competing Interests - References - Figure Legends - (Main Tables with legends if applicable) - Expanded View Figure Legends.

We formatted the manuscript accordingly.

- Our data editors checked the figure legends for accuracy and completeness. Please address the following points in the legends:
1) Please indicate the statistical test used for data analysis in the legend of figure 1A

We clarified this in the figure legend: "Wilcoxon rank-sum test (panels A-C)."

2) Please note that the box plots need to be defined in terms of minima, maxima, centre, bounds of box and whiskers, and percentile in the legends of figures 1A, B, C; 2C-I, 3B, C, D, F, H, I; 4A, D, E, F, G; 5A-D; EV1 B, EV2 E, H, J; EV3 A, B; EV4 A, EV5 A, C, D, F.

The box plots are now defined at the end of each figure legends: "Box plots indicate the median (central line), first and third quartiles (box bounds; 25th and 75th percentiles), and the minimum and maximum values (whiskers)."

3) Please note that information related to n is missing in the legends of figures 2J, K

The n for Figure 2J, K was already defined in the figure legend but might have been flagged as the recipient animals receiving both control and treated cells. Hence the number of recipient animals is reported.

We tried to clarify this accordingly: "**J**) Schematic and results of T cell homing analysis, comparing co-transferred of wild-type *vs.* catalytically inactive VPS34 $CD8^+$ T cells in a 1:1 ratio into recipient animals (n = 5). **K**) Comparison of vehicle *vs.* Apilimod-treated $CD8^+$ T cells in homing experiments (co-transferred cells in a 1:1 ratio into n = 6 recipient animals)."

4) Please note that the error bars are not defined in the legends of figures 2J, K; EV4 B, EV5G, H.

The error bars are now defined as "Panels J, K: Error bars = s.d.", "Panel B: Error bars = s.d.", and "Panels G, H: Error bars = s.d.".

5) Please note that the yellow dashed borders are not defined in the legend of figure EV2 G, EV3 C. This needs to be rectified.

We now describe the yellow dashed borders as: "Yellow dashed lines indicate cell outline."

6) Please note that the red circles are not defined in the legend of figure EV5 C. This needs to be rectified.

The red circles are now defined as: "Representative quantified vesicles are marked by a red circle.

- We perform a spot check of the supplied source data. I am not sure whether the image "Vehicle_2_YMIP" is the correct one? The "ZMIP" matches but this view seems different.

Thank you for pointing this out. We have carefully double checked the source images provided and the displayed ones in figure panel 1C and can confirm that we uploaded the right image (see screenshot below). As we are providing the raw two-channel tiff images the LUT might differ. We now made sure the channel color in the tiff metadata matches the displayed one.

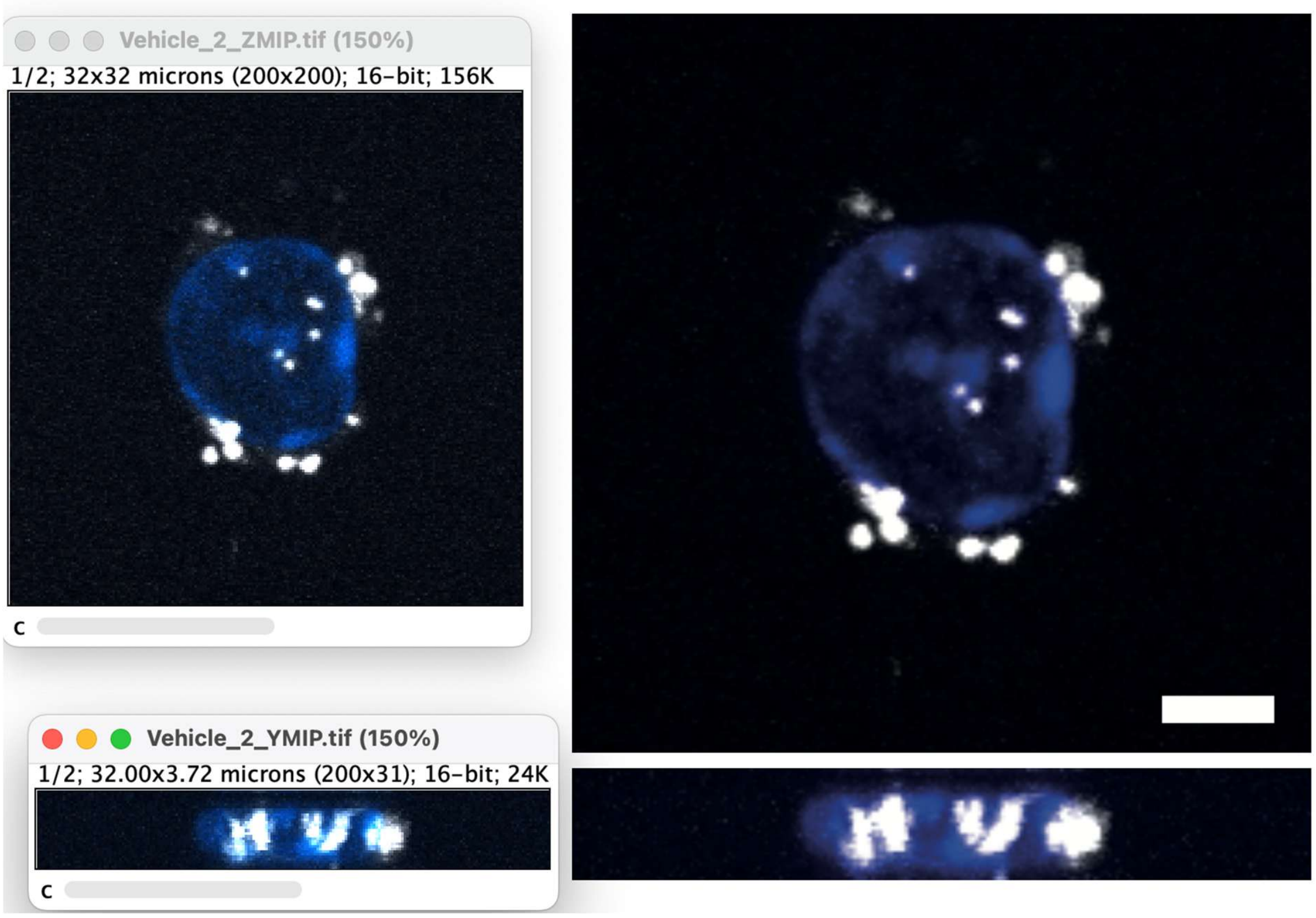

- Moreover, could you please upload the source data as individual folders per figure? Currently, all 5 folders are zipped into one file. Thank you.

We uploaded the source data as individually zipped folders.

Point-by-point response to referee reports for *Dehio et al.* (EMBOR-2025-63320-T)

Thank you very much for your message and for coordinating the review of our manuscript. We are grateful for the thoughtful evaluation by both referees. Please find a point-by-point response (in blue) to the referee comments (in *italics*)

Arbitrator/Referee #1:

This revised manuscript investigates the contribution of endo-lysosomal lipid kinase signaling (VPS34-PIKfyve axis) to the control of amoeboid cell migration in T cells and other amoeboid systems. The authors propose that a sequential cascade of VPS34- and PIKfyve-mediated $PI(3,5)P_2$ production regulates Ca^{2+} efflux from lysosomes through TRPML1, modulating myosin IIA activity and thereby controlling migration velocity. Overall, the study is ambitious and well presented, and the revisions since the previous review cycle are substantial and have greatly improved the manuscript.

We appreciate Referee #1's recognition of the scientific significance, technical soundness, and substantial progress achieved in our previous revision, as well as the constructive concerns regarding the interpretation of the presented data.

The paper is conceptually engaging and offers a potentially important framework linking endo-lysosomal dynamics and cytoskeletal control of T cell migration. The authors' efforts to address previous reviewer concerns are evident. Substantial new imaging, pharmacological, and mechanistic data have strengthened their conclusions. The high volume of revisions and additional experiments is commendable and demonstrates responsiveness to feedback.

However, while the data are compelling, my enthusiasm remains tempered by the global effects of the pathways studied, which complicate the mechanistic interpretation. The inhibitors used target central nodes of membrane trafficking and autophagy, and therefore their observed impact on migration may reflect widespread cellular dysfunction rather than a discrete, migration-specific mechanism dependent on local Calcium release, which nevertheless contributes to migration.

My main conceptual reservation lies in the pleiotropic impact of these kinases. Inhibition of VPS34 or PIKfyve inevitably affects endocytic trafficking, autophagy flux, and lysosomal homeostasis. Hence, disruptions in migration velocity could be secondary to broader alterations in cell physiology rather than directly attributable to localized endo-lysosomal lipid signaling. This confounding factor should be more explicitly addressed in the discussion. For instance, VPS34 inhibition has been reported to influence global protein synthesis rates, which directly contribute to cytoskeletal dynamics and energy metabolism. The authors might consider discussing and document whether reductions in biosynthetic capacity could also contribute to the observed reduction in motility.

We thank the referee for this important consideration that altering the VPS34–PIKfyve axis in migrating cells causes pleiotropic effects by altering PI3P levels on cell homeostasis. While, we agree with this view, we also believe that several elements of the current dataset already mitigate the concern that the migration phenotype arises solely from general cellular dysfunction. In particular, the convergence of multiple orthogonal perturbations, including VPS34 and PIKfyve inhibition, genetic manipulations, and downstream interference with the TRPML1-dependent Ca^{2+} channel, consistently produces a similar modulation of myosin II activity and migration velocity. Importantly, the rescue of VPS34 inhibition by a TRPML1 agonist, as well as the rescue by nocodazole treatment, which increases myosin IIA activity, clearly demonstrates a specific effect of the proposed signaling axis. This convergence argues that the observed phenotype reflects a specific signaling pathway rather than a nonspecific consequence of impaired trafficking or metabolism.

To address the referee's concern, we amended the discussion of the manuscript to the possible role pleiotropic effects on the presented data:

"We now show that the VPS34–PIKfyve axis links Ca^{2+} efflux from endo-lysosomes via TRPML1 to activation of myosin IIA. It should be noted, however, that PI(3)P levels and VPS34 activity have broad cellular functions including vesicular homeostasis, autophagy, and energy metabolism (Courreges et al., 2024; Jaber et al., 2016; Yang et al., 2021; Yuan et al., 2013). It is thus conceivable that, beyond the herein proposed signaling axis, yet to defined effects arising from disruption of PI(3)P–PI(3,5)P_2 homeostasis further impact amoeboid cell migration."

The data do not clearly establish whether the spatial localization of lysosomes is a cause or a consequence of the migratory phenotype. While polarized lysosome distribution is observed, it remains uncertain whether this polarization actively promotes migration, or merely reflects the migration state. Further clarification or experimental approaches targeting endosomal positioning regulators could be informative. For instance, the impact of nocodazole on endosome positioning is likely and how it counteracts VPS34 inhibition is not well dissected in the paper. Given that microtubule depolymerization itself alters endosomal trafficking and cell polarity, it is difficult to interpret this interaction mechanistically without additional markers or quantitative positioning analyses, that should yield substantial insights on the importance of positioning. Where is the MTOC in the polarized T cells responding to chemokines and how MT depolymerization impacts positing and affects migration? This also alludes to the distinction between secretory granules and endo/lysosomes in the migrating cells, which was mentioned in the previous round of review and the release of which is affected by calcium signaling.

The concern regarding establishing clear causality between the VPS34–PIKfyve axis, its positioning at the uropod, and migration is certainly valid. Importantly, the main conclusion from our study is that endo-lysosomal lipid signaling contributes to the regulation of amoeboid migration velocity through the VPS34–PIKfyve–TRPML1–myosin II pathway. Although we describe localization of this pathway to the uropod of migrating T cells, we have carefully

revised the manuscript to avoid implying that this specific spatial positioning of the endo-lysosome is itself causative for the observed phenotype.
Microtubules (MT) depolymerization by Nocodazole is well established to enhance myosin light chain activation through RhoA pathway activation, as described in the manuscript. We therefore used this known bypass of myosin light chain activation to rescue VPS34-dependent migratory defect. Increase of T cell migration velocity upon MT depolymerization has also been reported in previous studies and is consistent with our findings. The MTOC in these cells is likewise positioned at the uropod. While the interplay between MTs, the MTOC and vesicle positioning during amoeboid migration is certainly interesting, it is not central of this study and is therefore discussed only (please see below).
As the referee raised the issue of secretory granules and endo-lysosomes, we would like to emphasize that the majority of our data were obtained using naïve $CD8^{+}$ T cells, which are quiescent cells with minimal basal secretory activity.

In figure S6D, the authors present a graph showing the number of proteins with a FYVE domain across species.

As suggested, figure S6D and the associated text has now been removed from the revised manuscript.

This has no real interest and is purely speculative, unless it is used to disentangle position-dependent from metabolic effects of inhibiting VPS34, by silencing or editing regulators of endosomal positioning having direct or indirect interactions with PI3P, such as ARL8b, RILP, FYCO1, RUFY3, RAB7 or PLEKHM1. ARL8b, FYCO1 or RUFY3 are known to affect endosomal positioning and cell migration. Testing whether these manipulations phenocopy or diverge from VPS34-PIKfyve inhibition in terms of migration velocity would significantly strengthen the mechanistic argument and limit the interpretive breadth of the current dataset.

The referee suggested perturbing endosomal positioning regulators such as ARL8b or FYCO1, and we agree that such experiments might provide interesting insight into lysosome positioning mechanisms in ameboid cell migration. However, these proteins themselves regulate multiple aspects of endosomal trafficking and organelle dynamics and are redundantly expressed in T cells. Also, these targets primarily mediate the tethering of endo-lysosomes to microtubules via kinesin for plus-end-directed transport toward the cell periphery. Therefore, their perturbation would not necessarily revert the observed positioning effects, and redundancy might yield inconclusive results. For these reasons, we consider a thorough dissection of positioning regulators would likely constitute a substantial follow-up study beyond the scope of the current manuscript.

As we agree on the interesting nature of such experiments, we strengthened the discussion to reflect this important aspect:

“In an analogous manner, endo-lysosomes connected to microtubules by Arl8B or RILP/Rab7 have been shown to be re-positioned within cells depending on nutrient availability (Korolchuk et al., 2011; Scerra et al., 2022). Rab7b emerges as a potential co-modifier of the VPS34–PIKfyve axis in regulating amoeboid cell migration by tethering myosin II to lysosomes, thereby enabling its activation through TRPML1 (Borg et al., 2014; Vestre et al., 2021). Manipulating these adaptor proteins will help clarify how microtubule-dependent organelle positioning confines signaling to the uropod, and how this spatial regulation relates to amoeboid migration.”

Conclusion

This work is scientifically interesting and technically sound, with major progress since the previous review. Although, I dislike asking for further experimental addition at this stage, the study's mechanistic clarity is hindered by the broad cellular roles of VPS34 and PIKfyve, which challenge the specificity of the conclusions regarding migration regulation. Clarifying whether lysosomal positioning per se drives the observed effects and distinguishing this from general defects in trafficking and metabolism, would substantially enhance the manuscript's impact.

Arbitrator/Referee #2:

Thanks for an opportunity to look at this manuscript. Please note that I've read it as a generalist rather than a technical expert. As well, I've not seen the earlier version of the manuscript.

Overall, I think this presents a good-faith effort to respond to the reviewers. Of note,
a) The authors have included quite extensive new experimental manipulations, including shRNA depletion and CRISPR/Cas9 KO in T-cells that complement their drug inhibitor studies.
b) New experiments (including FIB-SEM studies) and quantitative (re)analysis of their data. Together, these seem very reasonable for a revision of a manuscript. Some of the other things requested by reviewers seem more suitable for a later study.

In terms of knowledge gain, this study represents a valuable advance in understanding how endolysosomal regulation contributes to cell migration. This is a long-standing question that was overshadowed by advances in elucidating how the cytoskeleton contributes to migration. Thus, it is valuable to develop this area further - especially as the current study identifies impacts on the cytoskeleton (myosin II activation and retrograde cortical flow) that help integrate these two drivers of migration.

I note that the authors have removed some material from the earlier version. Based on their response to reviewers, these seem justified. Certainly, in its current form the manuscript tells a tight, cohesive story.

In summary, this seems appropriate for EMBO Reports, both in knowledge gain and

experimental rigour. Whether there are technical issues in the new experiments that might require another look by the original reviewers is a matter that I will have to leave in your hands.

We are encouraged that Referee #2 considers the study suitable for publication in *EMBO Reports* and recognizes the substantial additional experiments and analyses included in this revision.

Prof. Christoph Hess
University of Basel
Department of Biomedicine
Switzerland

Dear Christoph,

Thank you for implementing the final minor changes. I am very pleased to accept your manuscript for publication in the next available issue of EMBO reports. Thank you for your contribution to our journal.

Your manuscript will be processed for publication by EMBO Press. It will be copy edited and you will receive page proofs prior to publication. Please note that you will be contacted by Springer Nature Author Services to complete licensing and payment information.

You may qualify for financial assistance for your publication charges - either via a Springer Nature fully open access agreement or an EMBO initiative. Check your eligibility: https://link.springer.com/journal/44319/how-to-publish-with-us

Should you be planning a Press Release on your article, please notify the production team via this form in order to coordinate publication and release dates: https://forms.cloud.microsoft/e/eH4A8JVP6g

If you have any questions, please do not hesitate to contact the Editorial Office. Thank you for your contribution to EMBO Reports.

Kind regards,

Martina

Martina Rembold, PhD
Senior Editor
EMBO reports

>>> Please note that it is EMBO Reports policy for the transcript of the editorial process (containing referee reports and your response letter) to be published as an online supplement to each paper. If you do NOT want this, you will need to inform the Editorial Office via email immediately. More information is available here: https://link.springer.com/partners/embo-press/editorial-policies#Peer%20review

EMBO Press Author Checklist

Corresponding Author Name: Christoph Hess	
Journal Submitted to: EMBO Reports	
Manuscript Number: EMBOR-2025-63320-T	

USEFUL LINKS FOR COMPLETING THIS FORM
The EMBO Journal - Author Guidelines
EMBO Reports - Author Guidelines
Molecular Systems Biology - Author Guidelines
EMBO Molecular Medicine - Author Guidelines

Reporting Checklist for Life Science Articles (updated January
This checklist is adapted from Materials Design Analysis Reporting (MDAR) Checklist for Authors. MDAR establishes a minimum set of requirements in transparent reporting in the life sciences (see Statement of Task: 10.31222/osf.io/9sm4x). Please follow the journal's guidelines in preparing your
Please note that a copy of this checklist will be published alongside your article.

Abridged guidelines for figures
1. Data
The data shown in figures should satisfy the following conditions:

- the data were obtained and processed according to the field's best practice and are presented to reflect the results of the experiments in an accurate and unbiased manner.
- ideally, figure panels should include only measurements that are directly comparable to each other and obtained with the same assay.
- plots include clearly labeled error bars for independent experiments and sample sizes. Unless justified, error bars should not be shown for technical
- if n<5, the individual data points from each experiment should be plotted. Any statistical test employed should be justified.
- Source Data should be included to report the data underlying figures according to the guidelines set out in the authorship guidelines on Data

2. Captions
Each figure caption should contain the following information, for each panel where they are relevant:

- a specification of the experimental system investigated (eg cell line, species name).
- the assay(s) and method(s) used to carry out the reported observations and measurements.
- an explicit mention of the biological and chemical entity(ies) that are being measured.
- an explicit mention of the biological and chemical entity(ies) that are altered/varied/perturbed in a controlled manner.
- the exact sample size (n) for each experimental group/condition, given as a number, not a range;
- a description of the sample collection allowing the reader to understand whether the samples represent technical or biological replicates (including how many animals, litters, cultures, etc.).
- a statement of how many times the experiment shown was independently replicated in the laboratory.
- definitions of statistical methods and measures:
  - common tests, such as t-test (please specify whether paired vs. unpaired), simple χ2 tests, Wilcoxon and Mann-Whitney tests, can be unambiguously identified by name only, but more complex techniques should be described in the methods section;
  - are tests one-sided or two-sided?
  - are there adjustments for multiple comparisons?
  - exact statistical test results, e.g., P values = x but not P values < x;
  - definition of 'center values' as median or average;
  - definition of error bars as s.d. or s.e.m.

Please complete ALL of the questions below.
Select "Not Applicable" only when the requested information is not relevant for your study.

Materials

Newly Created Materials	Information included in the manuscript?	In which section is the information available? (Reagents and Tools Table, Materials and Methods, Figures, Data Availability Section)
New materials and reagents need to be available; do any restrictions apply?	Not Applicable	

Antibodies	Information included in the manuscript?	In which section is the information available? (Reagents and Tools Table, Materials and Methods, Figures, Data Availability Section)
For **antibodies** provide the following information:<br>- Commercial antibodies: RRID (if possible) or supplier name, catalogue number and or/clone number<br>- Non-commercial: RRID or citation	Yes	Reagents and Tools Table, Materials and Methods

DNA and RNA sequences	Information included in the manuscript?	In which section is the information available? (Reagents and Tools Table, Materials and Methods, Figures, Data Availability Section)
**Short novel DNA or RNA including primers, probes**: provide the sequences.	Yes	Reagents and Tools Table, Materials and Methods

Cell materials	Information included in the manuscript?	In which section is the information available? (Reagents and Tools Table, Materials and Methods, Figures, Data Availability Section)
**Cell lines:** Provide species information, strain. Provide accession number in repository **OR** supplier name, catalog number, clone number, and/**OR** RRID.	Yes	Reagents and Tools Table, Material and Methods
**Primary cultures:** Provide species, strain, sex of origin, genetic modification status.	Yes	Material and Methods
Report if the cell lines were recently **authenticated** (e.g., by STR profiling) and tested for mycoplasma contamination.	Yes	Material and Methods

Experimental animals	Information included in the manuscript?	In which section is the information available? (Reagents and Tools Table, Materials and Methods, Figures, Data Availability Section)
**Laboratory animals or Model organisms:** Provide species, strain, sex, age, genetic modification status. Provide accession number in repository **OR** supplier name, catalog number, clone number, **OR** RRID.	Yes	Material and Methods
**Animal observed in or captured from the field:** Provide species, sex, and age where possible.	Not Applicable	
Please detail **housing and husbandry conditions**.	Yes	Material and Methods

Plants and microbes	Information included in the manuscript?	In which section is the information available? (Reagents and Tools Table, Materials and Methods, Figures, Data Availability Section)
**Plants:** provide species and strain, ecotype and cultivar where relevant, unique accession number if available, and source (including location for collected wild specimens).	Not Applicable	
**Microbes:** provide species and strain, unique accession number if available, and source.	Yes	Table 4

Human research participants	Information included in the manuscript?	In which section is the information available? (Reagents and Tools Table, Materials and Methods, Figures, Data Availability Section)
If collected and within the bounds of privacy constraints report on age, sex and gender or ethnicity for all study participants.	Not Applicable	

Core facilities	Information included in the manuscript?	In which section is the information available? (Reagents and Tools Table, Materials and Methods, Figures, Data Availability Section)
If your work benefited from core facilities, was their service mentioned in the acknowledgments section?	Yes	Acknowledgments

Design

Study protocol	Information included in the manuscript?	In which section is the information available? (Reagents and Tools Table, Materials and Methods, Figures, Data Availability Section)
If study protocol has been **pre-registered, provide DOI in the manuscript**. For clinical trials, provide the trial registration number **OR** cite DOI.	Not Applicable	
Report the **clinical trial registration number** (at ClinicalTrials.gov or equivalent), where applicable.	Not Applicable	

Laboratory protocol	Information included in the manuscript?	In which section is the information available? (Reagents and Tools Table, Materials and Methods, Figures, Data Availability Section)
Provide DOI OR other citation details if **external detailed step-by-step protocols** are available.	Not Applicable	

Experimental study design and statistics	Information included in the manuscript?	In which section is the information available? (Reagents and Tools Table, Materials and Methods, Figures, Data Availability Section)
Include a statement about **sample size** estimate even if no statistical methods were used.	Yes	Material and Methods
Were any steps taken to minimize the effects of subjective bias when allocating animals/samples to treatment (e.g. **randomization procedure**)? If yes, have they been described?	Not Applicable	
Include a statement about **blinding** even if no blinding was done.	Yes	Material and Methods
Describe **inclusion/exclusion criteria** if samples or animals were excluded from the analysis. Were the criteria pre-established?<br><br>If sample or data points were omitted from analysis, report if this was due to attrition or intentional exclusion and provide justification.	Yes	Material and Methods
For every figure, are **statistical tests** justified as appropriate? Do the data meet the assumptions of the tests (e.g., normal distribution)? Describe any methods used to assess it. Is there an estimate of variation within each group of data? Is the variance similar between the groups that are being statistically compared?	Yes	Material and Methods

Sample definition and in-laboratory replication	Information included in the manuscript?	In which section is the information available? (Reagents and Tools Table, Materials and Methods, Figures, Data Availability Section)
In the figure legends: state number of times the experiment was **replicated** in laboratory.	Yes	Figure legends
In the figure legends: define whether data describe **technical or biological replicates**.	Yes	Figure legends

Ethics

Ethics	Information included in the manuscript?	In which section is the information available? (Reagents and Tools Table, Materials and Methods, Figures, Data Availability Section)
Studies involving **human participants**: State details of **authority granting ethics approval** (IRB or equivalent committee(s), provide reference number for approval.	Not Applicable	
Studies involving **human participants**: Include a statement confirming that **informed consent** was obtained from all subjects and that the experiments conformed to the principles set out in the WMA Declaration of Helsinki and the Department of Health and Human Services Belmont Report.	Not Applicable	
Studies involving **human participants:** For publication of **patient photos**, include a statement confirming that consent to publish was obtained.	Not Applicable	
Studies involving experimental **animals**: State details of **authority granting ethics approval** (IRB or equivalent committee(s), provide reference number for approval. Include a statement of compliance with ethical regulations.	Yes	Material and Methods
Studies involving **specimen and field samples:** State if relevant **permits** obtained, provide details of authority approving study; if none were required, explain why.	Not Applicable	

Dual Use Research of Concern (DURC)	Information included in the manuscript?	In which section is the information available? (Reagents and Tools Table, Materials and Methods, Figures, Data Availability Section)
Could your study fall under dual use research restrictions? Please check biosecurity documents and list of **select agents and toxins** (CDC): https://www.selectagents.gov/sat/list.htm	Not Applicable	
If you used a select agent, is the security level of the lab appropriate and reported in the manuscript?	Not Applicable	
If a study is subject to dual use research of concern regulations, is the name of the **authority granting approval and reference number** for the regulatory approval provided in the manuscript?	Not Applicable	

Reporting

The MDAR framework recommends adoption of discipline-specific guidelines, established and endorsed through community initiatives. Journals have their own policy about requiring specific guidelines and recommendations to complement MDAR.

Adherence to community standards	Information included in the manuscript?	In which section is the information available? (Reagents and Tools Table, Materials and Methods, Figures, Data Availability Section)
State if relevant guidelines or checklists (e.g., **ICMJE, MIBBI, ARRIVE, PRISMA**) have been followed or provided.	Not Applicable	
For **tumor marker prognostic studies,** we recommend that you follow the **REMARK** reporting guidelines (see link list at top right). See author guidelines, under 'Reporting Guidelines'. Please confirm you have followed these guidelines.	Not Applicable	
For **phase II and III randomized controlled trials**, please refer to the **CONSORT** flow diagram (see link list at top right) and submit the CONSORT checklist (see link list at top right) with your submission. See author guidelines, under 'Reporting Guidelines'. Please confirm you have submitted this list.	Not Applicable	

Data Availability

Data availability	Information included in the manuscript?	In which section is the information available? (Reagents and Tools Table, Materials and Methods, Figures, Data Availability Section)
Have **primary datasets** been deposited according to the journal's guidelines (see 'Data Deposition' section) and the respective accession numbers provided in the Data Availability Section?	Not Applicable	
Were **human clinical and genomic datasets** deposited in a public access-controlled repository in accordance to ethical obligations to the patients and to the applicable consent agreement?	Not Applicable	
Are **computational models** that are central and integral to a study available without restrictions in a machine-readable form? Were the relevant accession numbers or links provided?	Not Applicable	
If publicly available data were reused, provide the respective **data citations in the reference list**.	Yes	Reference and materials and methods
