# Supplementary material for: A conserved VPS34-PIKfyve-TRPML1-myosin II axis regulates the speed of amoeboid cell migration
Source: EMBO Rep. 2026 Jul 7;27(16):4721–50. doi: 10.1038/s44319-026-00861-x (PMC13503823; doi:10.1038/s44319-026-00861-x)
Supplement: Supplementary file 2 [file 44319_2026_861_MOESM7_ESM.pdf]

Expanded View Figures

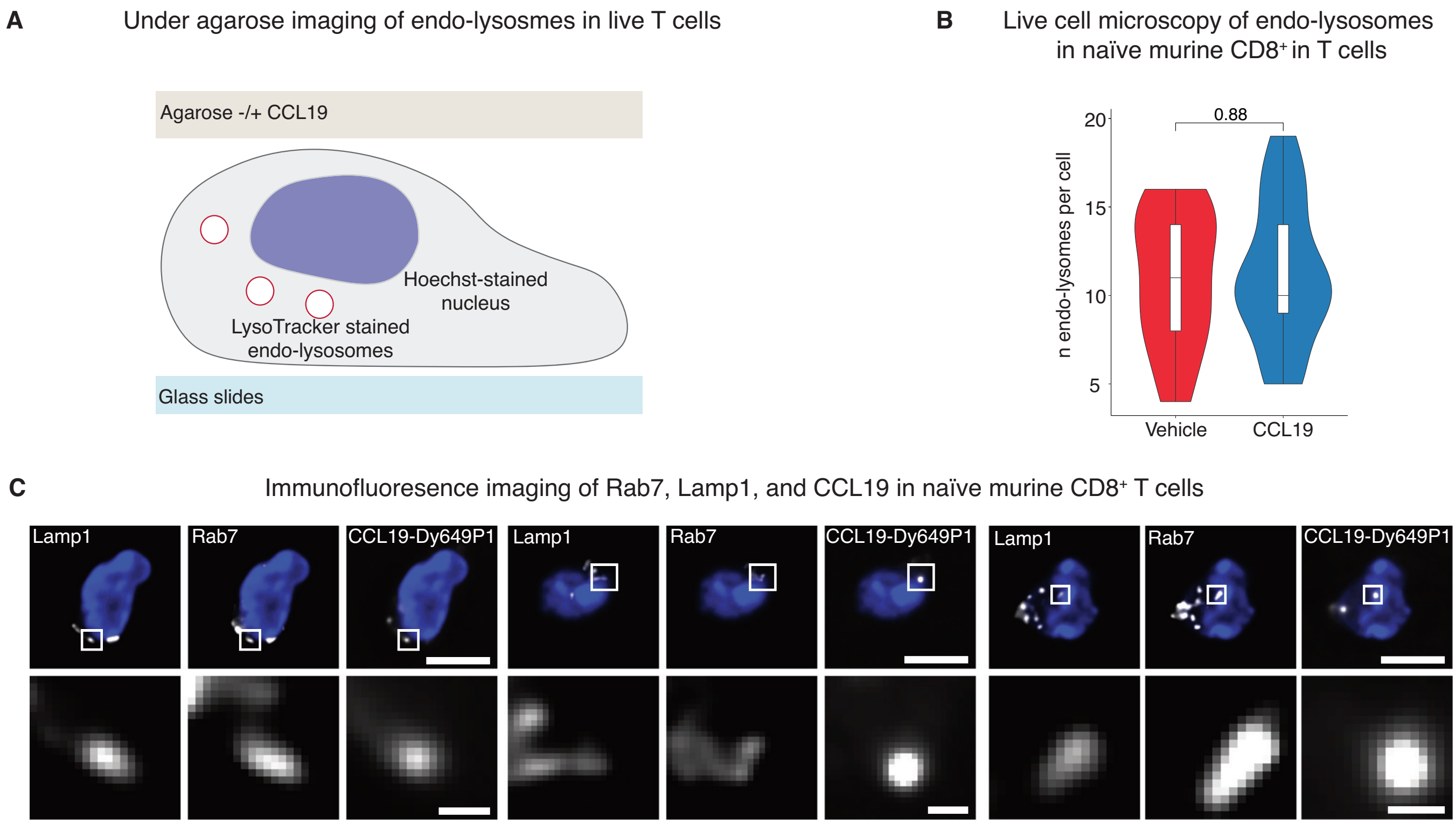

Figure EV1. Endo-lysosomes localize to the uropod of polarized T cells.

(**A**) Schematic of the under-agarose assay used to image endo-lysosomal polarization in live T cells. (**B**) Quantification of endo-lysosome numbers in naive murine CD8$^+$ T cells treated with vehicle ($n = 16$) or CCL19 ($n = 25$). (**C**) Representative fluorescence images of a naive murine CD8$^+$ T cell stained for Lamp1, Rab7 and exposed to CCL19-Dy649P1 (white). Top panels: merge with DAPI (blue; scale bars = 5 µm); box indicates region of interest magnified in bottom panels (scale bar = 500 nm). (**B**) Representative results of three independent experiments. Wilcoxon rank-sum test (**A**, **B**). Box plots indicate the median (central line), first and third quartiles (box bounds; 25th and 75th percentiles), and the minimum and maximum values (whiskers).

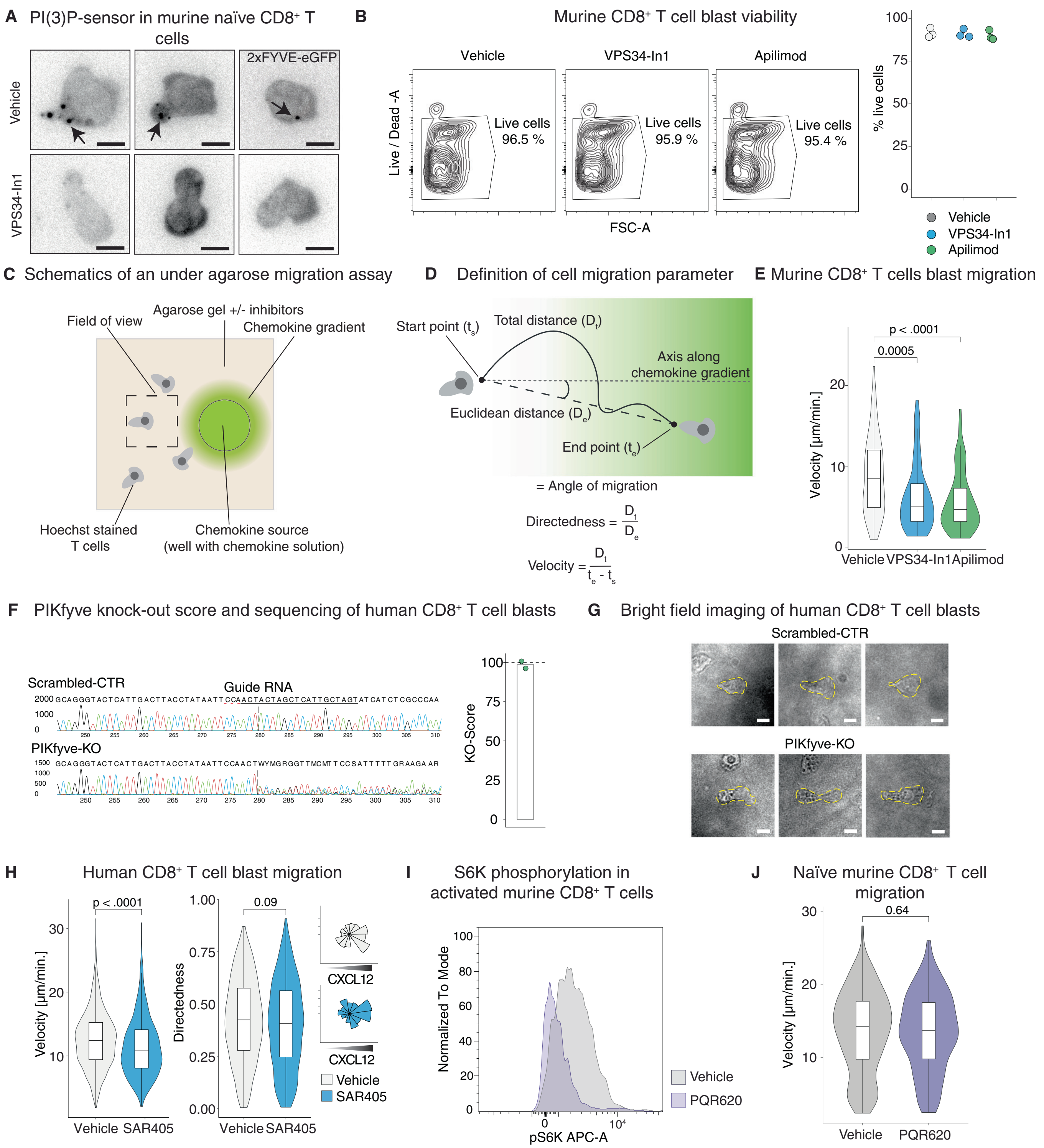
A PI(3)P-sensor in murine naïve CD8+ T cells
2xFYVE-eGFP
Vehicle
VPS34-In1
B Murine CD8+ T cell blast viability
Vehicle
VPS34-In1
Apilimod
Live / Dead -A
Live cells 96.5 %
Live cells 95.9 %
Live cells 95.4 %
FSC-A
% live cells
100
75
50
25
0
Vehicle
VPS34-In1
Apilimod
C Schematics of an under agarose migration assay
Field of view
Agarose gel +/- inhibitors
Chemokine gradient
Hoechst stained T cells
Chemokine source (well with chemokine solution)
D Definition of cell migration parameter
Start point (ts)
Total distance (Dt)
Axis along chemokine gradient
Euclidean distance (De)
End point (te)
= Angle of migration
Directedness = Dt / De
Velocity = Dt / te - ts
E Murine CD8+ T cells blast migration
p < .0001
0.0005
Velocity [µm/min.]
20
10
0
Vehicle
VPS34-In1
Apilimod
F PIKfyve knock-out score and sequencing of human CD8+ T cell blasts
Scrambled-CTR
Guide RNA
PIKfyve-KO
KO-Score
100
75
50
25
0
G Bright field imaging of human CD8+ T cell blasts
Scrambled-CTR
PIKfyve-KO
H Human CD8+ T cell blast migration
p < .0001
0.09
Velocity [µm/min.]
30
20
10
Directedness
1.00
0.75
0.50
0.25
0.00
CXCL12
CXCL12
Vehicle
SAR405
Vehicle SAR405
Vehicle SAR405
I S6K phosphorylation in activated murine CD8+ T cells
Normalized To Mode
100
80
60
40
20
0
pS6K APC-A
Vehicle
PQR620
J Naïve murine CD8+ T cell migration
0.64
Velocity [µm/min.]
30
20
10
Vehicle
PQR620

 © The Author(s)

Figure EV2. T cell velocity, but not directedness, is controlled by VPS34 and PIKfyve.

(**A**) Naive murine CD8$^+$ T cells transfected with the 2xFYVE–eGFP PI(3)P sensor, placed in a CCL19 gradient, and treated with vehicle or VPS34-In1. Arrows indicate PI(3)P$^+$ vesicles. Scale bar = 5 µm. (**B**) Left panel: representative live/dead staining plots (flow cytometry) of murine CD8$^+$ T cell blasts treated with vehicle, VPS34-In1, or Apilimod for 6 h. Right panel: percentage of live cells from three biological replicates. (**C**) Schematic (top view) of a T cell under-agarose migration assay. (**D**) Definitions of cell migration metrics used in this study. (**E**) Migration speed of murine CD8$^+$ T cell blasts in a CCL19 gradient, treated with vehicle control ($n = 66$), VPS34-In1 ($n = 81$), or Apilimod ($n = 79$). (**F**) Left: representative bulk Sanger sequencing of control *vs*. PIKfyve-KO human CD8$^+$ T cell blasts of the region flanking the first guide RNA (gRNA). The gRNA sequence is underlined, the cutting side indicated by a dashed vertical line. Right: knockout score ($n = 2$). (**G**) Bright-field images of control and PIKfyve-KO human CD8$^+$ T cells placed in a CCL19 gradient. Yellow dashed lines indicate cell outline. Scale bar = 10 µm. (**H**) Velocity and directedness of human CD8$^+$ T cell blasts migrating in a CXCL12 gradient, treated with the VPS34 inhibitor SAR405 (velocity and angles: $n = 1838$, directedness: $n = 694$) or vehicle control (velocity and angles: $n = 1400$, directedness: $n = 594$). (**I**) Representative histogram (flow cytometry) of pS6K expression among murine CD8$^+$ T cells activated for 24 h with CD3 and CD28 targeting antibodies and treated with the mTOR inhibitor PQR620 (purple) *vs*. vehicle control (grey). (**J**) Velocity of naive murine CD8$^+$ T cells in a CCL19 gradient, following treatment with vehicle ($n = 272$) or the mTOR inhibitor PQR602 ($n = 232$). (**A**, **B**, **G–J**) Representative of ≥3 independent experiments; (**I**) representative of two independent experiments. Wilcoxon rank-sum test (**E**, **H**, **J**). Box plots indicate the median (central line), first and third quartiles (box bounds; 25th and 75th percentiles), and the minimum and maximum values (whiskers).

© The Author(s)

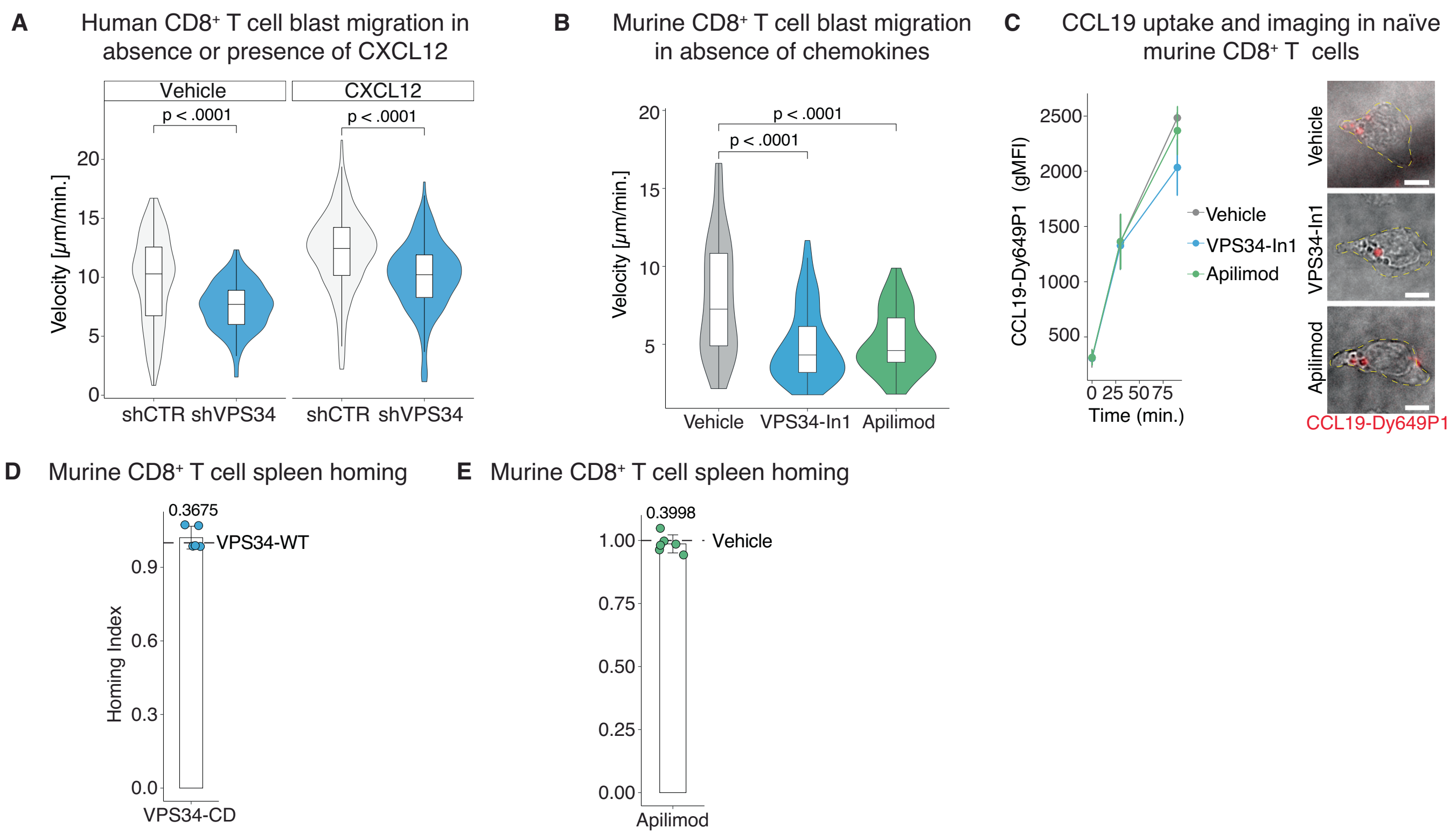

Figure EV3. Chemokine signaling is dispensable for VPS34–PIKfyve-mediated regulation of T cell migration speed.

(**A**) Migration speed in the absence or presence of a CXCL12 gradient, of human CD8$^+$ T cell blasts expressing scrambled control shRNA (shCTR; vehicle: $n = 196$; CXCL12: $n = 326$) or VPS34 knockdown (shVPS34; vehicle: $n = 62$; CXCL12: $n = 210$). (**B**) Migration speed of murine CD8$^+$ T cell blasts treated with vehicle ($n = 90$), VPS34-In1 ($n = 76$), or Apilimod ($n = 46$) in the absence of chemokines. (**C**) Left panel: Time course analysis of CCL19-Dy649P1 uptake in naive murine CD8$^+$ T cells (flow cytometry, geometric mean intensity (gMFI) and standard deviation of three biological replicates). Right panel: Representative bright field images merged with CCL19-Dy649P1 signal (red) of naive murine CD8$^+$ T cells migrating in a fluorescent CCL19 gradient, treated with vehicle control, VPS34-In1, or Apilimod. Yellow dashed lines indicate cell outline. Scale bar = 5 µm. (**D**) Spleen homing index (normalized to each internal control) of murine CD8$^+$ T cells per recipient mice ($n = 5$) expressing the wild-type or catalytic dead (CD) VPS34, and (**E**) spleen homing index (normalized to each internal control, $n = 6$ recipient mice) of vehicle or Apilimod-treated cells. (**A–C**) Representative of three experiments; (**D**, **E**) representative of two experiments. Wilcoxon rank-sum test (**A**, **B**) and paired *t* test (**D**, **E**). (**D**, **E**) Error bars = s.d. Box plots indicate the median (central line), first and third quartiles (box bounds; 25th and 75th percentiles), and the minimum and maximum values (whiskers).

 © The Author(s)

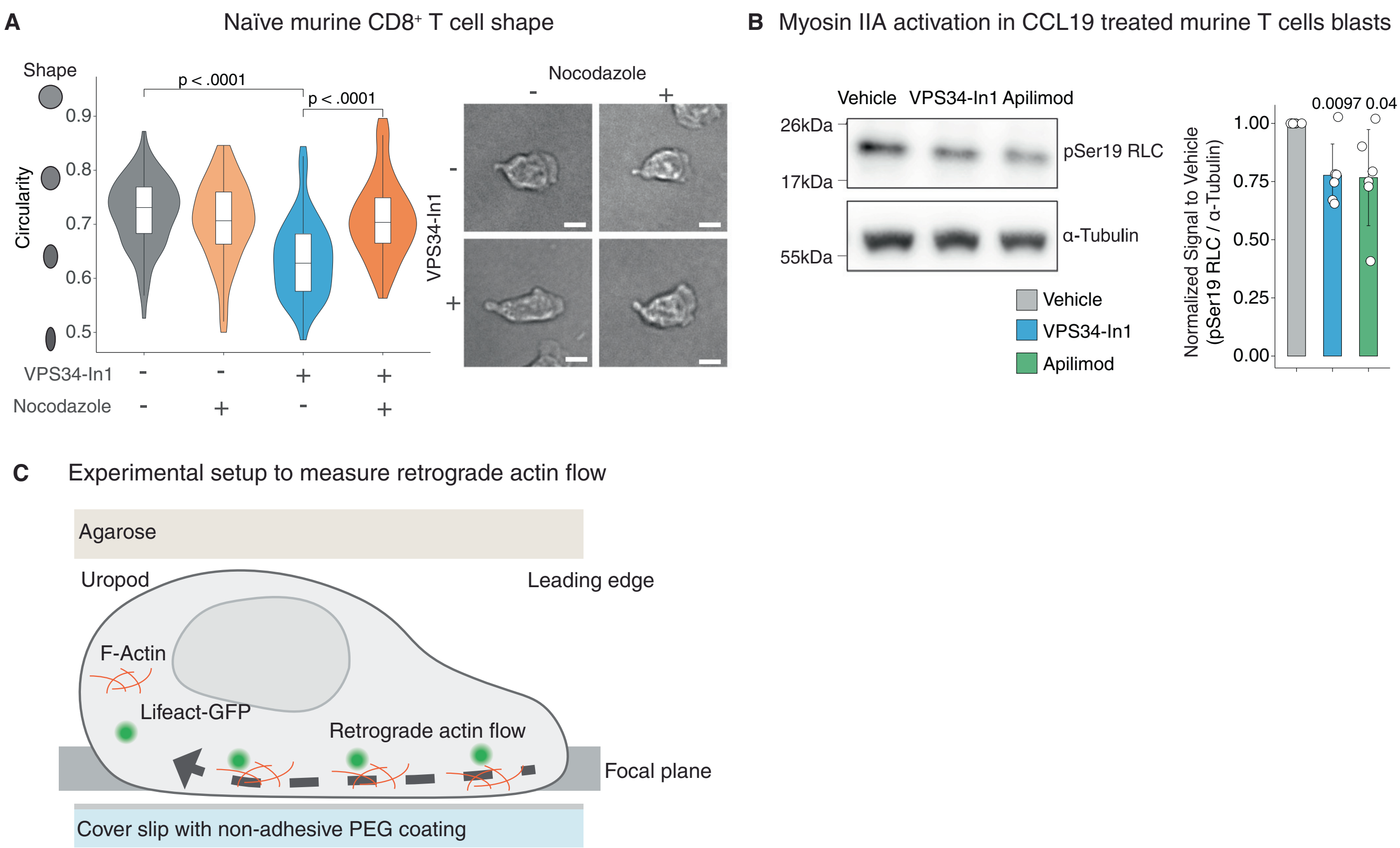

Figure EV4. VPS34 and PIKfyve regulate retrograde actin flow and myosin IIA activation.

(**A**) Left panel: Circularity of naive CD8+ T cells placed in a CCL19 gradient treated with vehicle control ($n = 81$), Nocodazole ($n = 70$), VPS34-In1 ($n = 85$), and VPS34-In1 plus Nocodazole ($n = 56$). Right panels: representative bright field images. Scale bar: 5 µm. (**B**) Left panels: representative Western blots probing for RLC Ser19 phosphorylation and α-tubulin (loading control) in T cell blasts treated with CCL19 and vehicle, VPS34-In1, or Apilimod. Right panel: quantification of six biological replicates, normalized to α-tubulin and vehicle. (**C**) Experimental setup for assessing retrograde actin flow in murine T cell blasts expressing the Lifeact-GFP reporter (to visualize F-actin dynamics). (**A**) Displays representative data of three independent experiments. ART ANOVA for (**A**). Paired *t* test for (**B**). (**B**) Error bars = s.d. Box plots indicate the median (central line), first and third quartiles (box bounds; 25th and 75th percentiles), and the minimum and maximum values (whiskers).

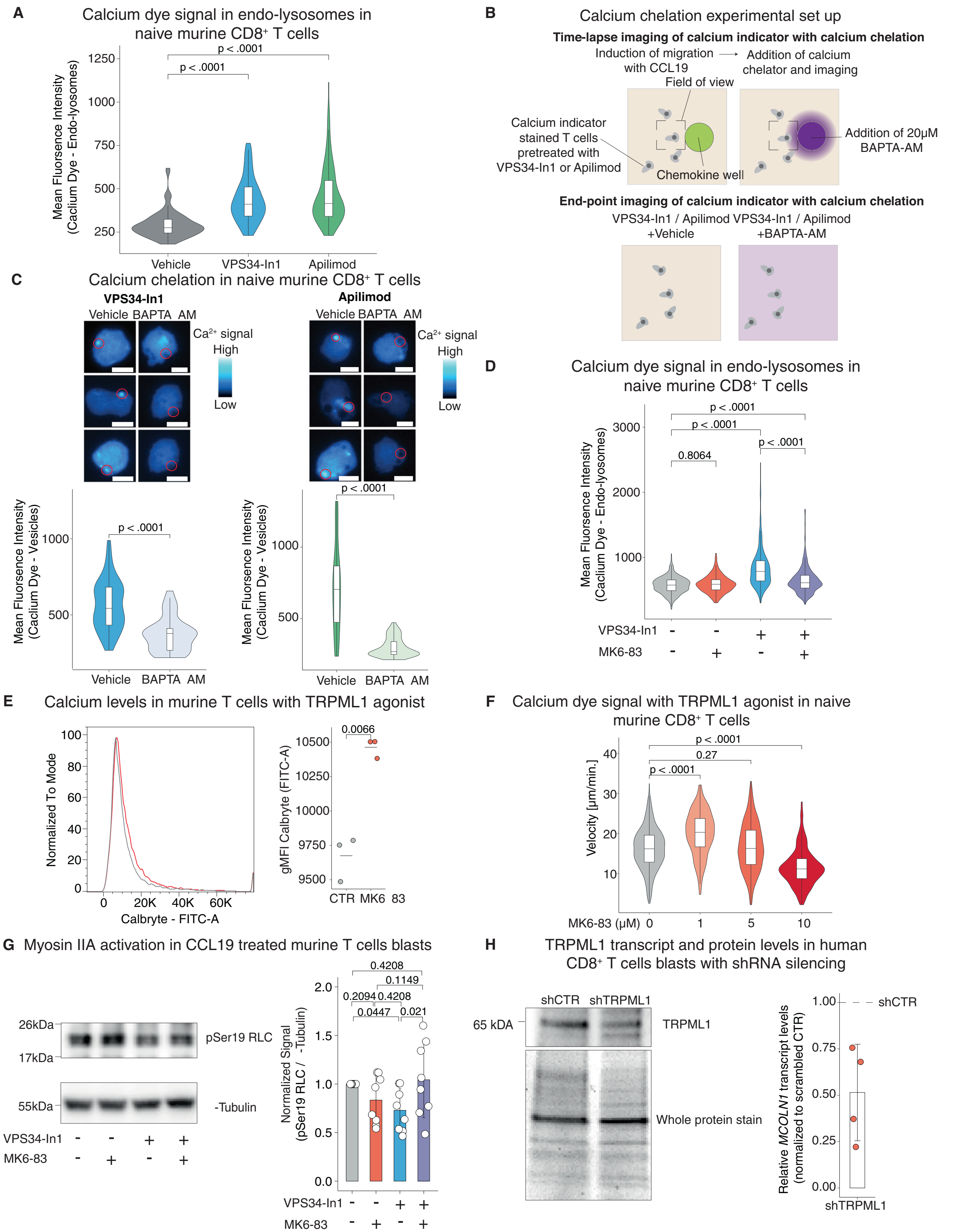
A
Calcium dye signal in endo-lysosomes in naive murine CD8+ T cells
Mean Fluorescence Intensity (Caclium Dye - Endo-lysosomes)
1250
1000
750
500
250
p < .0001
p < .0001
Vehicle
VPS34-In1
Apilimod
B
Calcium chelation experimental set up
Time-lapse imaging of calcium indicator with calcium chelation
Induction of migration with CCL19
Addition of calcium chelator and imaging
Field of view
Calcium indicator stained T cells pretreated with VPS34-In1 or Apilimod
Chemokine well
Addition of 20μM BAPTA-AM
End-point imaging of calcium indicator with calcium chelation
VPS34-In1 / Apilimod +Vehicle
VPS34-In1 / Apilimod +BAPTA-AM
C
Calcium chelation in naive murine CD8+ T cells
VPS34-In1
Apilimod
Vehicle BAPTA AM
Ca2+ signal
High
Low
Mean Fluorescence Intensity (Caclium Dye - Vesicles)
1000
500
p < .0001
Vehicle
BAPTA AM
D
Calcium dye signal in endo-lysosomes in naive murine CD8+ T cells
Mean Fluorescence Intensity (Caclium Dye - Endo-lysosomes)
3000
2000
1000
p < .0001
p < .0001
p < .0001
0.8064
VPS34-In1 - - + +
MK6-83 - + - +
E
Calcium levels in murine T cells with TRPML1 agonist
Normalized To Mode
100
80
60
40
20
0
0 20K 40K 60K
Calbryte - FITC-A
gMFI Calbryte (FITC-A)
10500
10250
10000
9750
9500
0.0066
CTR MK6 83
F
Calcium dye signal with TRPML1 agonist in naive murine CD8+ T cells
Velocity [μm/min.]
40
30
20
10
p < .0001
0.27
p < .0001
MK6-83 (μM) 0 1 5 10
G
Myosin IIA activation in CCL19 treated murine T cells blasts
26kDa
17kDa
55kDa
pSer19 RLC
-Tubulin
VPS34-In1 - - + +
MK6-83 - + - +
Normalized Signal (pSer19 RLC / -Tubulin)
2.0
1.5
1.0
0.5
0.0
0.4208
0.1149
0.2094
0.4208
0.0447
0.021
H
TRPML1 transcript and protein levels in human CD8+ T cells blasts with shRNA silencing
shCTR
shTRPML1
65 kDA
TRPML1
Whole protein stain
Relative MCOLN1 transcript levels (normalized to scrambled CTR)
1.00
0.75
0.50
0.25
0.00
shCTR
shTRPML1

 © The Author(s)

Figure EV5. VPS34 and PIKfyve regulate migration velocity of T cells via lysosomal Ca^{2+}.

(**A**) Quantification of calcium dye MFI per endo-lysosome in naive murine $CD8^+$ T cells migrating in a CCL19 gradient, treated with vehicle ($n = 82$ vesicles in 28 cells), VPS34-In1 ($n = 75$ vesicles in 19 cells), or Apilimod ($n = 152$ vesicles in 39 cells). (**B**) Experimental setup for calcium chelation: naive murine $CD8^+$ T cells stained with Calbryte were treated with VPS34-In1 or Apilimod and placed under agarose. For time-lapse imaging, cells migrated in a CCL19 gradient, then medium was replaced with BAPTA-AM-containing medium. For endpoint imaging, cells were incubated for 30 min at 37 °C with or without VPS34-In1, Apilimod, and/or BAPTA-AM. (**C**) Upper panels: representative images of Calbryte-stained naive murine $CD8^+$ T cells with or without VPS34-In1, Apilimod, and/or BAPTA-AM. Lower panels: quantification of calcium dye MFI per endo-lysosome for VPS34-In1 ($n = 72$ vesicles in 32 cells), VPS34-In1 + BAPTA-AM ($n = 41$ vesicles in 18 cells), Apilimod ($n = 25$ vesicles in 10 cells), or Apilimod plus BAPTA-AM ($n = 64$ vesicles in 23 cells). Representative quantified vesicles are marked by a red circle. Scale bar = 5 µm. (**D**) Quantification of calcium dye MFI per endo-lysosome in naive murine $CD8^+$ T cells migrating in a CCL19 gradient, treated with vehicle ($n = 335$ vesicles in 93 cells), MK6-83 ($n = 194$ vesicles in 65 cells), VPS34-In1 ($n = 518$ vesicles in 108 cells), or VPS34-In1 plus MK6-83 ($n = 450$ vesicles in 121 cells). (**E**) Left panel: Representative histogram of cytosolic calcium levels as captured by flow cytometry in murine T cells stained with Ca^{2+} dye and treated with MK6-83 (5 µM) or vehicle. Right panel: Summary of $n = 3$ technical replicates. (**F**) Migration velocity of naive murine $CD8^+$ T cells placed in a CCL19 gradient, treated with vehicle ($n = 646$), or increasing concentrations of MK6-83 (1 µM: $n = 173$; 5 µM: $n = 487$; 10 µM: $n = 262$). (**G**) Left panel: representative Western blots probing for RLC Ser19 phosphorylation (top) and α-tubulin (bottom; loading control) in T cell blasts treated with CCL19 and vehicle, VPS34-In1, and/or MK6-83. Right panel: quantification of eight biological replicates normalized to α-tubulin and vehicle. (**H**) Left panel: representative Western blots probing for TRPML1 (*top*) and whole protein stain (bottom) in human $CD8^+$ T cells transduced with scrambled control shRNA (shCTR) or TRPML1-targeting shRNA (shTRPML1). Right panel: relative *MCOLN1* transcript levels from four biological replicates, normalized to SDHA and scrambled control. (**A**, **C**, **D**, **F**) Representative of ≥3 independent experiments. Two-way ANOVA with Fisher's least-squares test (**G**), *t* test (**E**), Wilcoxon rank-sum test (**A**, **C**), ART ANOVA (**D**). (**G**, **H**) Error bars = s.d. Box plots indicate the median (central line), first and third quartiles (box bounds; 25th and 75th percentiles), and the minimum and maximum values (whiskers).

© The Author(s)

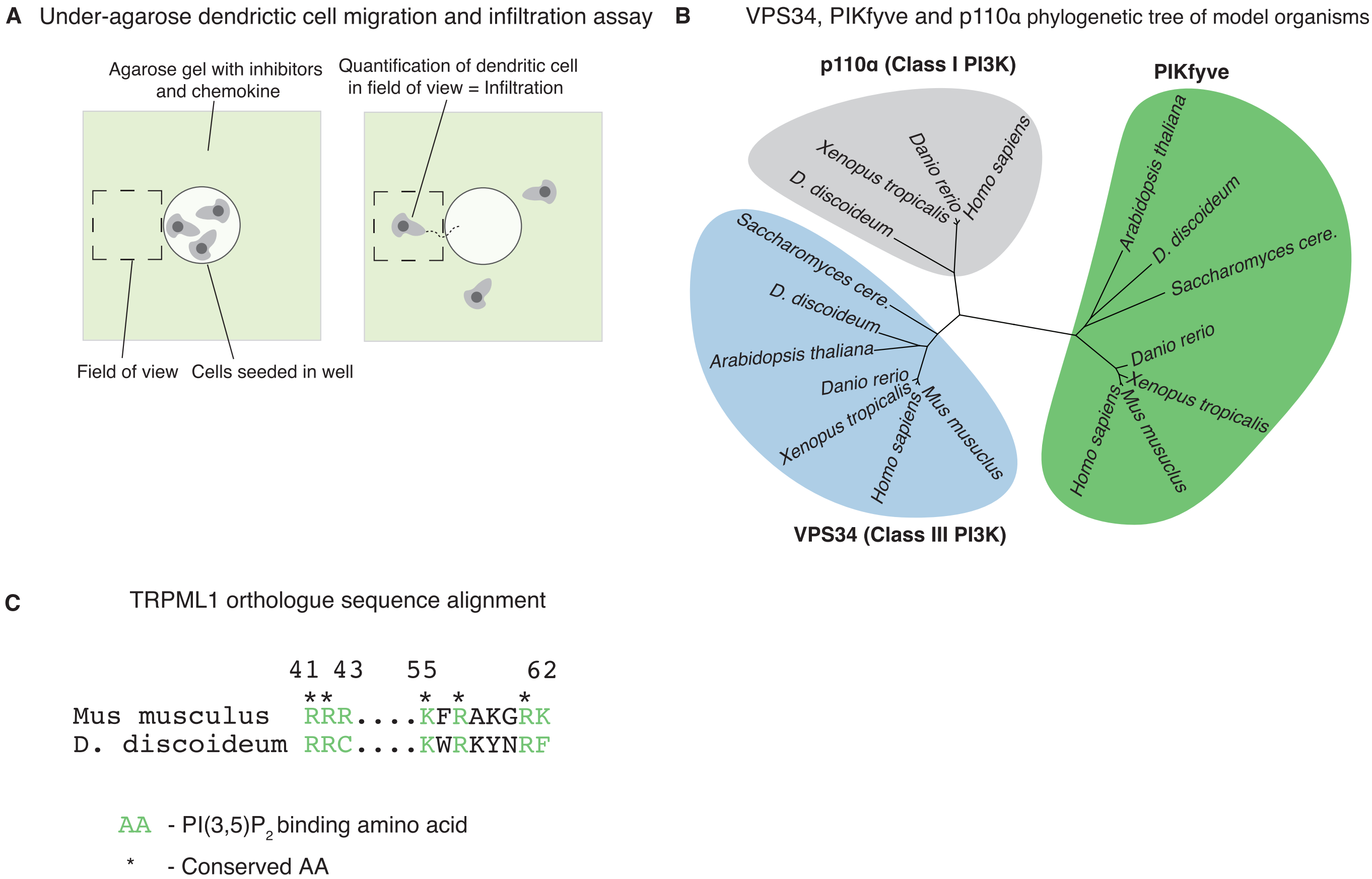

Figure EV6. Regulation of amoeboid migration is a conserved function of the VPS34–PIKfyve axis.

(**A**) Schematics of the migration and infiltration assay of DCs: Cells were added to the well in the agarose (containing inhibitors and CCL19, left panel). DCs crawled under the gel (= infiltration) and the number of cells in the field of view was quantified (right panel). (**B**) Phylogenetic analysis of the amino acid (AA) sequences of VPS34, PIKFYVE, and p110α (as an example for Class I PI3K). (**C**) Alignment of the PI(3,5)P_2-binding protein region from *Mus musculus* TRPML1 and the *D. discoideum* orthologue. PI(3,5)P_2-binding AAs are highlighted in green, conserved AAs indicated by an asterisk.

 © The Author(s)
